# Author Correction: Genetically encoded discovery of perfluoroaryl macrocycles that bind to albumin and exhibit extended circulation in vivo

**DOI:** 10.1038/s41467-023-43517-3

**Published:** 2023-11-21

**Authors:** Jeffrey Y. K. Wong, Arunika I. Ekanayake, Serhii Kharchenko, Steven E. Kirberger, Ryan Qiu, Payam Kelich, Susmita Sarkar, Jiaqian Li, Kleinberg X. Fernandez, Edgar R. Alvizo-Paez, Jiayuan Miao, Shiva Kalhor-Monfared, J. Dwyer John, Hongsuk Kang, Hwanho Choi, John M. Nuss, John C. Vederas, Yu-Shan Lin, Matthew S. Macauley, Lela Vukovic, William C. K. Pomerantz, Ratmir Derda

**Affiliations:** 1 Department of Chemistry, University of Alberta, Edmonton, AB T6G 2G2 Canada; 2 Department of Chemistry, University of Minnesota, Minneapolis, MN 55455 USA; 3 Department of Chemistry and Biochemistry, University of Texas at El Paso, El Paso, TX 79968 USA; 4 Department of Chemistry, Tufts University, Medford, MA 02155 USA; 5 Ferring Research Institute, San Diego, CA 92121 USA; 6 Quantum Intelligence Corp., 31F, One IFC, 10 Gukjegeumyung-ro, Yeongdeungpo-gu-Seoul, Republic of Korea; 7 Department of Medical Microbiology and Immunology, University of Alberta, Edmonton, AB T6G 2E1 Canada

**Keywords:** Chemical modification, Screening, Peptides

Correction to: *Nature Communications* 10.1038/s41467-023-41427-y, published online 13 September 2023

The original version of this Article contained an error in the spelling of the author name Jiaqian Li, which was previously incorrectly spelled as Jianqian Li. This error has now been corrected in both the PDF and HTML versions of the Article.

