# Supplementary material for: Genetically encoded discovery of perfluoroaryl macrocycles that bind to albumin and exhibit extended circulation in vivo
Source: Nat Commun. 2023 Sep 13;14:5654. doi: 10.1038/s41467-023-41427-y (PMC10499988; doi:10.1038/s41467-023-41427-y)
Supplement: Supplementary file 1 — Supplementary Information [file 41467_2023_41427_MOESM1_ESM.pdf]

Supplementary information for

Genetically Encoded Discovery of Perfluoroaryl Macrocycles that Bind to Albumin and Exhibit Extended Circulation in vivo

Jeffrey Y.K. Wong,[1†] Arunika Ekanayake,[1†] Serhii Kharchenko,[1] Steven E. Kirberger,[2] Ryan Qiu,[1] Payam Kelich[3], Susmita Sarkar[1], Jianqian Li,[2] Kleinberg Fernandez,[1] Edgar R. Alvizo-Paez[1], Jiayuan Miao,[4] Shiva Kalhor-Monfared[1], John, J. Dwyer[5], Hongsuk Kang,[6] Hwanho Choi,[6] John M. Nuss[5], John C. Vederas[1], Yu-Shan Lin[4], Matthew S Macauley,[1] Lela Vukovic[3], William C.K. Pomerantz[2], and Ratmir Derda[1‡]

1. Department of Chemistry, University of Alberta, Edmonton, AB T6G 2G2, Canada
2. Department of Chemistry, University of Minnesota, Minneapolis, MN 55455, USA
3. Department of Chemistry and Biochemistry, University of Texas at El Paso, El Paso, TX 79968, U.S.A.
4. Department of Chemistry, Tufts University, Medford, MA 02155, USA
5. Ferring Research Institute, San Diego, CA 92121, USA.
6. Quantum Intelligence Corp., Seoul, Republic of Korea

† These authors contributed equally

‡ Corresponding author: ratmir@ualberta.ca

Table of contents

Supplementary Methods 7
1. List of abbreviations: 7
2. Biochemistry methods 8
3. Preparation of SXCX$_3$C phage-displayed library 8
4. Panning strategy 1: panning on plate 8
5. Panning strategy 2: panning on plate and in solution 9
Supplementary Fig. 1. First panning campaign of **OFS**-phage library against the HSA 10
Supplementary Fig. 2. Second panning campaign of **OFS-**phage library against the HSA .. 11
Supplementary Fig. 3. Quantification of modification of SXC$_3$C libraries by **DFS**. 12
Supplementary Fig. 4. Phage recovery from the second selection campaign of round 3. 13
6. Panning strategy 3: panning in solution 13
Supplementary Fig. 5. A heat map of 85 hits discovered from the third panning campaign. 14
7. General protocol for protein biotinylation 15
8. PCR amplification protocol for Illumina deep sequencing 15
Supplementary Fig. 6. PCR amplification protocol for Illumina deep sequencing. 16
9. Illumina sequencing of samples before and after panning 16
Supplementary Table 1: URL for 1st panning campaign deep sequencing results 17
Supplementary Table 2: URL for 2nd panning campaign deep sequencing results 17
Supplementary Table 3: URL for 3rd panning campaign deep sequencing results 17
10. General chemistry methods 18
11. Peptide synthesis 18
12. General protocol for cyclization with decafluorodiphenylsulfone 18
13. General protocol for cyclization with pentaflurophenyl-sulfide 18
14. General protocol for cyclization with hexafluorobenzene (HFB) 18
15. General protocol for cyclization with decafluorobiphenyl (DFB) 18
16. Synthesis of N-terminally PEG$_6$ labeled peptides 19
17. Synthesis of N-terminally Bodipy labeled peptides 19
18. Cysteine alkylation of peptides with iodoacetamide (IAA) 19
19. Formation of disulfide bridges on peptides 19
20. Cyclization of peptides with MBX 19

21. Apelin analogue synthesis **19**

22. N_3-PEG_6-Apelin analogue synthesis **20**

23. PFS-Apelin analogue synthesis[4] **20**

24. ^{19}F NMR binding experiment **21**

Supplementary Table 4: A typical experiment involving titration with HSA.. 21

25. Fluorescence polarization binding assay **21**

26. Isothermal titration calorimetry (ITC) binding assay **22**

27. *In vivo* pharmacokinetic experiment **22**

28. LC–MS analysis for pharmacokinetics **22**

29. Docking Calculations **23**

30. Docking simulations for free energy calculations of 14c and alanine scans **24**

Supplementary Fig. 7. DFS stapled peptide reacted with GSH over 3 hours.. 26

Supplementary Fig. 8. Stability of the **PFS** stapled peptides 27

Supplementary Table 5: Summary of the selected peptide sequences.. 28

Supplementary Fig. 9. Ramachandran plot of the cyclic peptide backbone for **14b** and **14c** 29

Supplementary Fig. 10. Summary of the ^{19}F NMR binding measurement.. 30

Supplementary Fig. 11. ^{19}F NMR measurement of binding of **9b-17b** and **9c-17c** to HSA.. 31

Supplementary Fig. 12. Binding of **14c** and **17c** to human and rat serum albumin.. 32

Supplementary Fig. 13. ITC experiment with 4 mM peptide and 0.4 mM albumin in PBS . 33

Supplementary Fig. 14. ITC experiment with 1 mM peptide and 0.1 mM albumin in PBS. 34

Supplementary Fig. 15. ITC experiment with 1 mM peptide and 0.1 mM albumin in PBS. 35

Supplementary Fig. 16. FP binding assay between albumin and peptide-BODIPY. 36

Supplementary Fig. 17. Binding of **PFS**-SICRFFC with C- or N-term BODIPY to HSA. 37

Supplementary Fig. 18. Comparison of **DFS** and **PFS**-modified peptides.. 38

Supplementary Fig. 19. ^{19}F NMR comparison of BODIPY labeled and unlabeled peptides 38

Supplementary Fig. 20. Competition of **14c** and albumin binders measured by ^{19}F NMR... 39

Supplementary Fig. 21. HSA binding sites of known ligands.. 40

Supplementary Fig. 22. Docking calculations for **PFS**-SICRFFCGGG (**14c**) and HSA. 41

Supplementary Fig. 23. Docking scores of **14c** and HSA binding sites of fatty acids. 42

Supplementary Table 6: Binding scores of **14c** to HSA all fatty acid binding sites 43

Supplementary Table 7: Ligands that overlap with **14c** at the FA1 IB binding site. 43

Supplementary Table 8: Amino acids that contact **14c** and Hemin in HSA IB (1O9X)....... 44

Supplementary Fig. 24. Superposition of calculated **14c**-HSA complex and crystal structures of HSA binding to four different ligands in FA1 IB site. 45

Supplementary Fig. 25. The pulling force profiles of SMD simulations.............................. 46

Supplementary Fig. 26. PMFs calculation of 14c in different binding sites.......................... 46

Supplementary Fig. 27. Hydrogen bond analyses for **14c** in different binding sites............. 47

Supplementary Fig. 29. Analysis of distances between **14c** and different binding pockets.. 48

Supplementary Table 9: λ-schedule used in FEP calculations. 49

Supplementary Fig. 30. Detection of macrocycles using mass spectrometry........................ 50

Supplementary Fig. 31. Standard curves for selected ions of SA-21, **14k-j-m-l-c**, and **17c**. 51

Supplementary Fig. 32. Standard curves for selected ions of **21-25c**, **14g-i**, **20c-h-n**, **26c**... 52

Supplementary Fig. 33. Pharmacokinetic studies for **26c**, **17c** and SA-21........................... 53

Supplementary Table 10: Summary of in vivo experiments.. 53

31. MATLAB script for DE analysis 54

32. Summary of synthesis 61

Supplementary Fig. 34. Synthesis summary of **9b DFS-**STCHDITCGGKKK 61

Supplementary Fig. 35. Synthesis summary of **9c PFS**-STCHDITCGGKKK 62

Supplementary Fig. 36. Synthesis summary of **10b DFS**-STCHYIGCGGKKK.................. 63

Supplementary Fig. 37. Synthesis summary of **10c PFS**-STCHYIGCGGKKK................... 64

Supplementary Fig. 38. Synthesis summary of **11c PFS**-STCHANCGGG.......................... 65

Supplementary Fig. 39. Synthesis summary of **12b DFS**-STCHANGCGGKKK 66

Supplementary Fig. 40. Synthesis summary of **12c PFS**-STCHANGCGGKKK 67

Supplementary Fig. 41. Synthesis summary of **13b DFS**-STCHTIYCGGKKK 68

Supplementary Fig. 42. Synthesis summary of **13c PFS**-STCHTIYCGGKKK 69

Supplementary Fig. 43. Synthesis summary of **14b DFS**-SICRFFCGGG............................ 70

Supplementary Fig. 44. Synthesis summary of **14c PFS**-SICRFFCGGG............................. 71

Supplementary Fig. 45. Synthesis summary of **15b DFS**-SFCPMFGGG............................. 72

Supplementary Fig. 46. Synthesis summary of **15c PFS**-SFCPMFGGG 73

Supplementary Fig. 47. Synthesis summary of **16b DFS**-SLCKRECGGG.......................... 74

Supplementary Fig. 48. Synthesis summary of **16c PFS**-SLCKRECGGG........................... 75

Supplementary Fig. 49. Synthesis summary of **17b DFS**-STCQGECGGG 76

Supplementary Fig. 50. Synthesis summary of **17c PFS**-STCQGECGGG 77

Supplementary Fig. 51. Synthesis summary of **PFS-**SICRFFGGG-BODIPY (**18d**)............ 78

Supplementary Fig. 52. Synthesis summary of BODIPY-**PFS**-SICRFFGGG (**14e**) 79

Supplementary Fig. 53. Synthesis summary of **PFS**-SICRFFGGG-PEG4 (**14i**)................. 80
Supplementary Fig. 54. Synthesis summary of **PFS**-STCQGECGGK-BODIPY (**19d**)....... 81
Supplementary Fig. 55. Synthesis summary of HFB-SICRFFGGG (**14j**) 82
Supplementary Fig. 56. Synthesis summary of DFB-SICRFFGGG (**14k**) 83
Supplementary Fig. 57. Synthesis summary of IAA-SICRFFGGG (**14l**) 84
Supplementary Fig. 58. Synthesis summary of MBX-SICRFFGGG (**14m**)......................... 85
Supplementary Fig. 59. Synthesis summary of PFS-AICRFFGGG (**21c**) 86
Supplementary Fig. 60. Synthesis summary of PFS-SACRFFGGG (**22c**) 87
Supplementary Fig. 61. Synthesis summary of PFS-SICAFFGGG (**23c**)............................ 88
Supplementary Fig. 62. Synthesis summary of PFS-SICRAFGGG (**24c**) 89
Supplementary Fig. 63. Synthesis summary of PFS-SICRFAGGG (**25c**) 90
Supplementary Fig. 64. Synthesis summary of SICRFFCGGGZ (**20a**)............................... 91
Supplementary Fig. 65. Synthesis summary of **PFS**-SICRFFGGGZ (**20c**)......................... 92
Supplementary Fig. 66. Synthesis summary of Np7-**PFS**-SICRFFGGG (**14g**)................... 93
Supplementary Fig. 67. Synthesis summary of **PFS**-SICRFFGGGZp4 (**20h**)...................... 94
Supplementary Fig. 68. Synthesis summary of **PFS**-SICRFFCGGGZa (**20n**) 95
Supplementary Fig. 69. STCHDITCGGKKK (**9a**) ^{1}H NMR Spectra.................................. 96
Supplementary Fig. 70. STCHYIGCGGKKK (**10a**) ^{1}H NMR Spectra................................ 96
Supplementary Fig. 71. STCHANCGGKKK (**12a**) ^{1}H NMR Spectra................................. 97
Supplementary Fig. 72. STCHTIYCGGKKK (**13a**) ^{1}H NMR Spectra................................ 97
Supplementary Fig. 73. SICRFFCGGG (**14a**) ^{1}H NMR Spectra 98
Supplementary Fig. 74. SLCKRECGGG (**16a**) ^{1}H NMR Spectra 98
Supplementary Fig. 75. STCQGECGGG (**17a**) ^{1}H NMR Spectra 99
Supplementary Fig. 76. **DFS**-STCHDITCGGKKK (**9b**) ^{1}H NMR Spectra.......................... 99
Supplementary Fig. 77. **DFS**-STCHYIGCGGKKK (**10b**) ^{1}H NMR Spectra 100
Supplementary Fig. 78. **DFS**-STCHANCGGKKK (**12b**) ^{1}H NMR Spectra....................... 100
Supplementary Fig. 79. **DFS**-STCHTIYCGGKKK (**13b**)^{1}H NMR Spectra....................... 101
Supplementary Fig. 80. **PFS**-STCHDITCGGKKK (**9c**) ^{1}H NMR Spectra......................... 101
Supplementary Fig. 81. **PFS**-STCHTIYCGGKKK (**10c**) 1H NMR Spectra 102
Supplementary Fig. 82. **PFS**-STCHANCGGKKK (**12c**) ^{1}H NMR Spectra....................... 102
Supplementary Fig. 83. **PFS**-STCHYIGCGGKKK (**13c**) ^{1}H NMR Spectra 103
Supplementary Fig. 84. **PFS-**SICRFFCGGG (**14c**) ^{1}H NMR Spectra 103

Supplementary Fig. 85. PFS-SICRFFCGGG (**14c**) ^{1}H NMR Spectra 104
Supplementary Fig. 86. PFS-SFCPMFCGGG (**15c**) ^{1}H NMR Spectra 104
Supplementary Fig. 87. PFS-SLCKRECGGG (**16c**) 1H NMR Spectra 105
Supplementary Fig. 88. PFS-SACRFFCGGG (**21c**) ^{1}H NMR Spectra............................... 105
Supplementary Fig. 89. PFS-SICAFFCGGG (**22c**) ^{1}H NMR Spectra................................ 106
Supplementary Fig. 90. PFS-SICRAFCGGG (**23c**) ^{1}H NMR Spectra 106
Supplementary Fig. 91. PFS-SICRFACGGG (**24c**) ^{1}H NMR Spectra 107
Supplementary Fig. 92. HFB-SICRFFCGGG (**12j**) ^{1}H NMR Spectra 107
Supplementary Fig. 93. DFB-SICRFFCGGG (**12k**) 1H NMR Spectra............................. 108
Supplementary Fig. 94. IA-SICRFFCGGG (**14l**) ^{1}H NMR Spectra 108
Supplementary Fig. 95. MBX-SICRFFCGGG (**14m**) ^{1}H NMR Spectra 109
Supplementary Fig. 96. Np7-**PFS**-SICRFFCGGG (**14g**) ^{1}H NMR Spectra........................ 110
Supplementary Fig. 97. PFS-SICRFFCGGGZp4 (**20h**) 1H NMR Spectra........................ 111
Supplementary Fig. 98. PFS-SICRFFCGGGZa (**20n**) ^{1}H NMR Spectra 112
Supplementary References... 112

Supplementary Methods

1. List of abbreviations:

ACN	Acetonitrile
BIA	biotin-PEG2-iodoacetamide
Boc	*tert*-butyloxycarbonyl
BODIPY	4,4-difluoro-4-bora-3a,4a-diaza-s-indacene
BSH	biotin-thiol
Da.	dalton(s)
DFB	decafluorobiphenyl
DFS	decafluorodiphenylsulfone
DCM	dichloromethane
DMF	N, N-Dimethylformamide
ESI	electrospray ionization
eq.	equivalent(s)
EDT	1,2-ethanedithiol
h	hour(s)
HFB	hexafluorobenzene
HSA	human serum albumin
HPLC	high performance liquid chromatography
HRMS	high-resolution mass spectrometry
IAA	iodoacetamide
LCMS	liquid chromatography mass spectrometry
MBX	1,3-Bis(bromomethyl)benzene
min	minute
MHz	megahertz
MsCl	methanesulfonyl chloride
mL	milliliter(s)
mM	millimolar
min	minute(s)
mmol	millimolar
PBS	phosphate buffered saline
PCR	polymerase chain reaction
PFS	pentafluorophenylsulfide
ppm	parts per million
rt	room temperature
RSA	rat serum albumin
sec	second(s) (time)

t-Bu-OH	tert-Butyl alcohol
TCEP	tris(2-carboxyethyl)phosphine)
THPTA	tris(3-hydroxypropyltriazolylmethyl)amine
TIS	triisopropylsilane
TFA	trifluoroacetic acid
Tris	tris(hydroxymethyl)aminomethane
v/v	volume/volume
Z	L-propargylglycine

2. Biochemistry methods

HSA was purchased from Sigma-Aldrich (cat# A4327-1G), as were Protein A (Sigma Aldrich, cat# P6031-1MG), and Concanavalin A (cat# C2010-100MG). The proteins were immobilized on a high binding plate (Corning, ref# 3369) with 100 µL of HSA or Protein A (100 µg/mL). The wells were washed 6 times with 200 µL of 1×PBS-T+0.1% tween 20, at pH 7.4 prior to phage incubation. The proteins were biotinylated in section **7**. Prior to capture proteins, the magnetic streptavidin beads (Promega, cat# Z5482) were washed with PBS. All proteins were captures with 20 µL magnetic streptavidin beads.

3. Preparation of SXCX$_3$C phage-displayed library

The procedures have been adopted and modified as previously described in two publications that produced the M13-displayed SXCXXXC library[1] and M13-SDB vector[2]. In short, the vector SB4 QFT*LHQ was digested with Kpn I HF (NEB cat# R3142S) and Eag I HF (NEB cat# R3505S). A primer/template pair consisting of primer 5'-AT GGC GCC CGG CCG AAC CTC CAC C-3' and template 5'-CC CGG GTA CCT TTC TAT TCT CAC TCT TCT X TGT XXX TGT GGT GGA GGT TCG GCC GGG CGC TTG ATT-3' with 'X' representing a trinucleotide formed by annealing. The primer/template was then extended using Klenow DNA polymerase (NEB) according to the manufacturer's instructions. The insert fragment was then digested with Kpn1 HF and Eag1 HF, gel purified, and ligated into the cut vector. The ligation products were then transformed into electrocompetent *E. coli* cells, and the transformants were grown overnight on *E. coli* TG1 to allow for phage production. Phage cultures were then centrifuged to remove cells and debris, and then the phage was precipitated by PEG precipitation (5% PEG 0.5 M NaCl). Other SDB vectors have been processed identically. We sequenced the naïve libraries by Illumina sequencing, and the naïve library of SXCX$_n$C (n=3-5) composition is publicly available at the following link: https://48hd.cloud/file/1470. SXCX$_4$C and SXCX$_5$C libraries were prepared as described in previously reported protocols.[3]

4. Panning strategy 1: panning on plate

Round 1-3: The following protocol was repeated 3 times: In Protein A coated wells, 100 µL of 2×10^9 PFU/mL **DFS** modified library or unmodified library was incubated for O/N at 4 °C to remove unspecific binders. The **DFS** modified library supernatant was then transferred to wells with HSA and incubated for 1.5 h at RT. In parallel, **DFS** modified library supernatants were also incubated with Protein A, and unmodified libraries were

incubated with HSA and protein A as a negative controls. After panning, all wells were washed 10 times with PBST (Phosphate buffered saline + 0.1% Tween). The phage particles remaining in the wells were eluted with 200 µL of glycine elution buffer (200 mM Glycine-HCl pH 2.2, 0.1% BSA) for 9 min. The elution buffer was transferred into a new 1.7 mL microcentrifuge tube containing 20 µL of neutralization buffer (1 M Tris-HCl, pH 9.1). The recovered phage solution was amplified for the next round of panning and PCR amplified for deep sequencing.

5. Panning strategy 2: panning on plate and in solution

Round 1: In Protein A coated wells, 100 µL of 2×10^9 PFU/mL **DFS** modified library or unmodified library was incubated for O/N at 4 °C to remove unspecific binders. The **DFS** modified library supernatant was then transferred to wells with HSA and incubated for 1.5 h at RT. In parallel, **DFS** modified library supernatants were also incubated with Protein A, and unmodified libraries were incubated with HSA and protein A as a negative controls. After incubation, all wells were washed 10 times with PBST. The phage particles remaining in the wells were eluted with 200 µL of glycine elution buffer (200 mM Glycine-HCl pH 2.2, 0.1% BSA) for 9 min. The elution buffer was transferred into a new 1.7 mL microcentrifuge tube containing 20 µL of neutralization buffer (1 M Tris-HCl, pH 9.1). The recovered phage solution was amplified for the next round of panning and PCR amplified for deep sequencing.

Round 2: A suspension of 50 µL of magnetic streptavidin beads was transferred to a 1.7 mL centrifuge tube and washed with 1 mL of PBST. The beads were then resuspended in 1 mL of blocking buffer (PBS+2% milk) and incubated for 1 h at 4 °C. In parallel, 200 µL (2×10^9 PFU/mL) of **DFS** modified phage and unmodified phage library were incubated for 1 h at RT with pre-blocked beads to remove any beads binders. The beads were immobilized with a magnetic rack and the supernatant was transferred to a new 1.7 mL centrifuge tube for panning. A solution of 100 µL of depleted **DFS** modified phage library was combined with 10 µg of biotinylated HSA, increased the volume to 200 µL with PBS, and incubated for 1 h at RT. In parallel, 100 µL of the depleted **DFS** modified phage was combined with 10 µg of biotinylated protein A, increased the volume to 200 µL with PBS and incubated for 1 h at RT. A solution of 100 µL of unmodified phage library that was depleted against Streptavidin beads was combined with 10 µg of biotinylated protein A and HSA, increased the volume to 200 µL with PBS and incubated for 1 hour at RT. After incubation, 50 µL of blocked streptavidin beads were added to the samples. Then, the beads were captured with a magnetic rack and washed 10 times with 1 mL of 1×PBST. The bound phage particles were eluted with 200 µL of glycine elution buffer (200 mM Glycine-HCl pH 2.2, 0.1% BSA) for 9 min. The elution buffer was transferred into a new 1.7 mL microcentrifuge tube and neutralized with 20 µL of 1 M Tris-HCl (pH 9.1). The recovered phage solution was amplified for the next round of panning and PCR amplified for deep sequencing.

Round 3: In Protein A coated wells, 100 µL of 2×10^9 PFU/mL **DFS** modified library or unmodified library was incubated for 1 h at 4 °C to remove unspecific bindings. The **DFS** modified library supernatant was then transferred to wells with HSA and incubated for 1.5 h at RT. In parallel, depleted **DFS** modified library supernatants were also incubated with Protein A. Unmodified libraries were incubated with HSA and protein A as negative

controls. After incubation, all wells were washed 10 times with 1×PBST. The remaining phage particles were eluted with 200 µL of glycine elution buffer (200 mM Glycine-HCl pH 2.2, 0.1% BSA) for 9 min. The elution buffer was transferred into a new 1.7 mL microcentrifuge containing 20 µL of neutralization buffer (1 M Tris-HCl, pH 9.1). The recovered phage solution was amplified for the next round of biopanning and PCR amplified for deep sequencing.

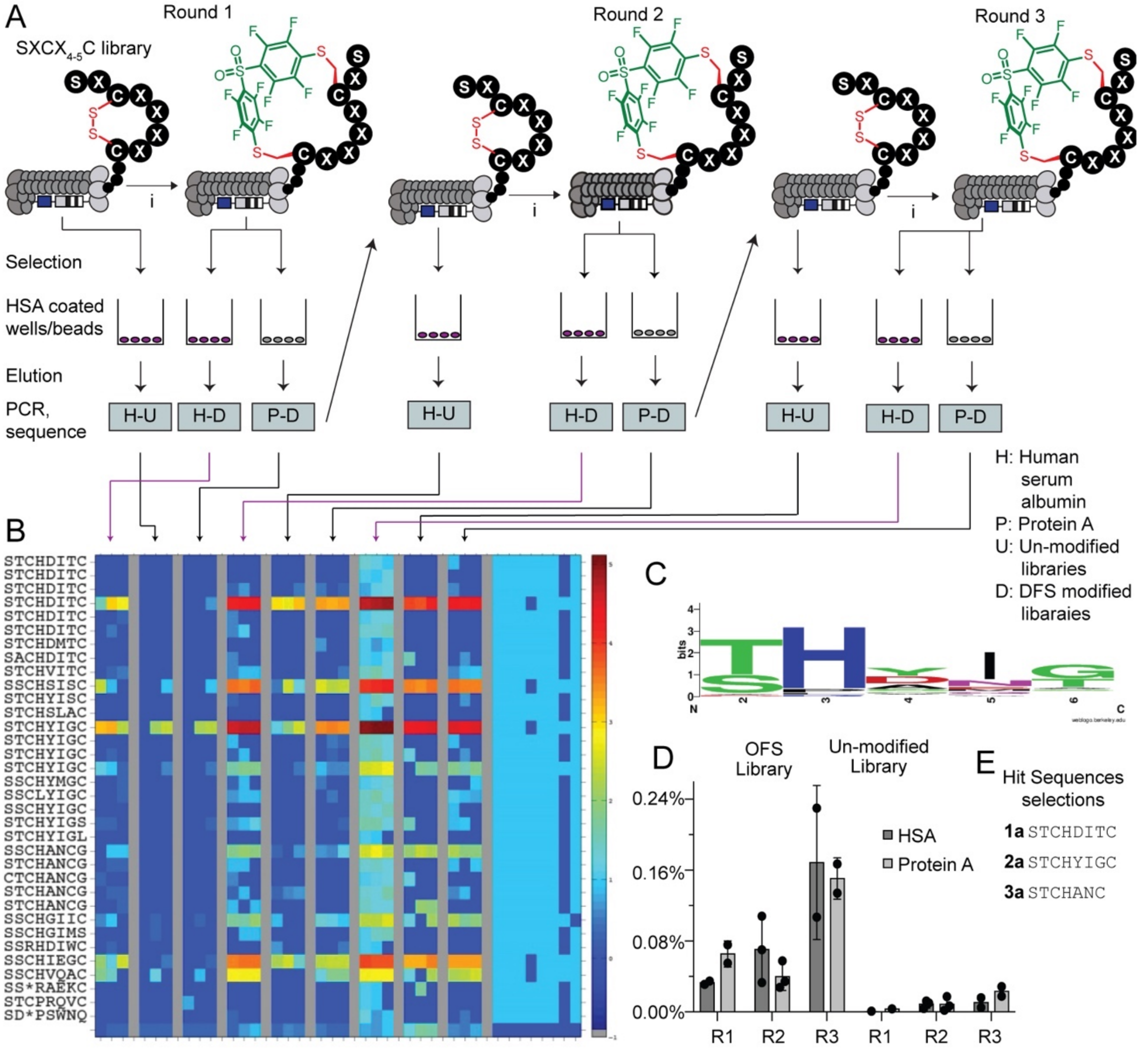

Supplementary Fig. 1. First panning campaign of **OFS**-phage library against the HSA (A) A scheme of three rounds panning against HSA and negative controls (B) The top 39 sequences from differential enrichment (DE) results (C) LOGO analysis plot of the enriched sequences. (D) Percentage of the phage recovery after each round of bio-panning. (E) Selected sequences for chemical synthesis of the macrocycles.

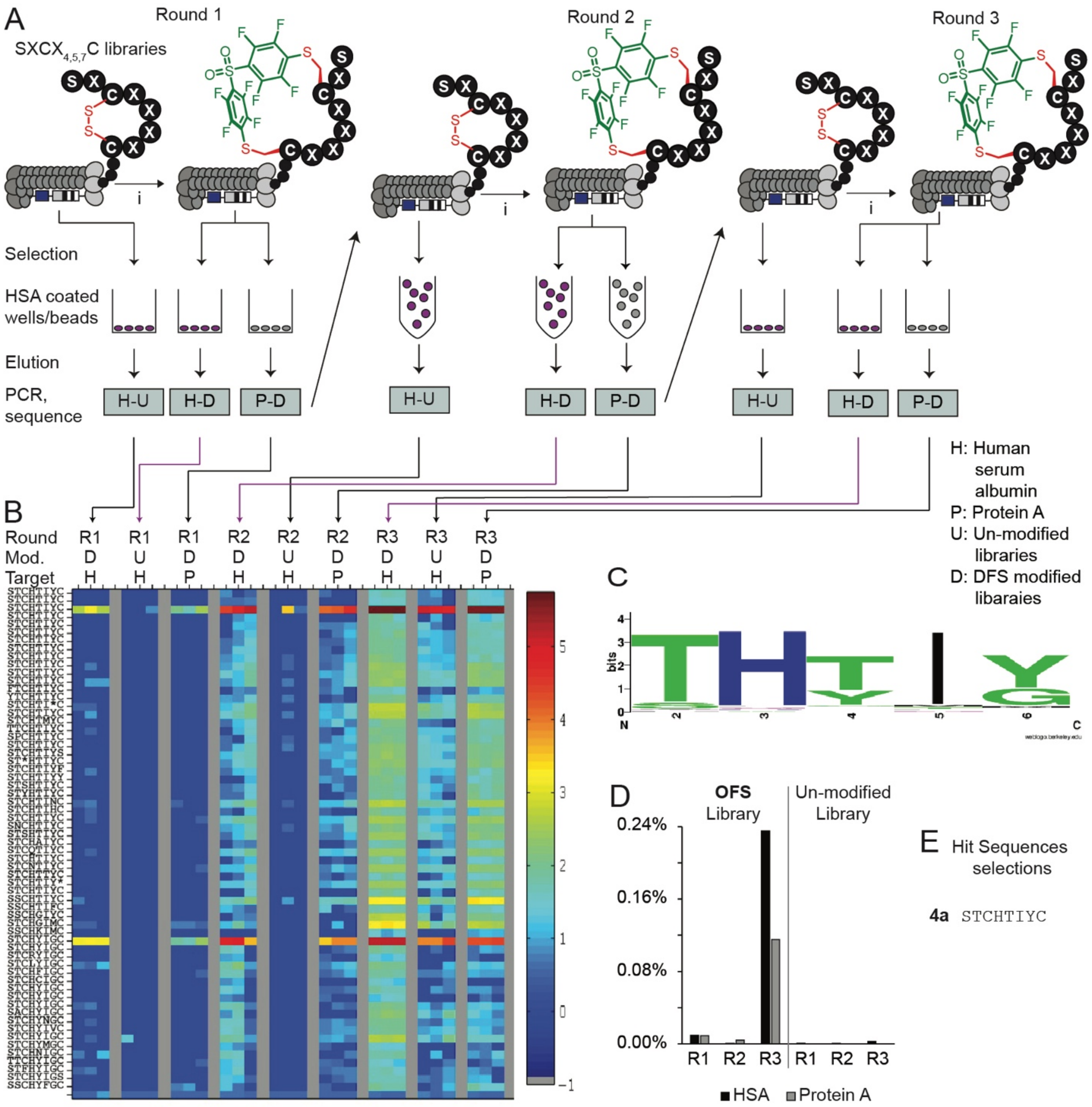

Supplementary Fig. 2. Second panning campaign of **OFS-**phage library against the HSA (A) Scheme of a three-rounds panning against HSA and the negative control (B) The top 65 sequences from differential enrichment results. (C) LOGO analysis plot of the enriched sequences (D) Percentage of the phage recovery after each round of bio-panning (E) Selected sequences for validation synthesized into macrocycles

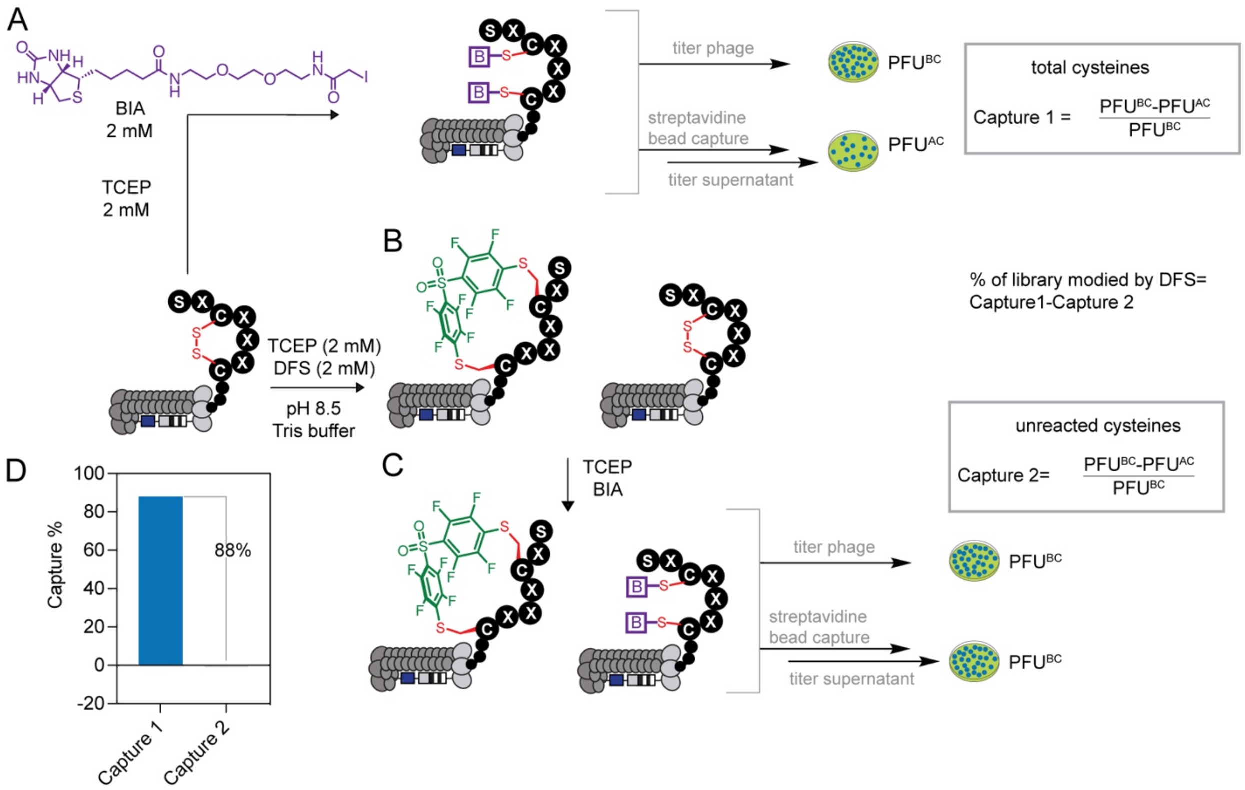

Supplementary Fig. 3. Quantification of modification of SXC$_3$C libraries by **DFS**. (A) M13-phage displayed disulfide library was reduced with TCEP and the exposed cysteine thiols were modified with biotin-peg iodoacetamide (BIA). The streptavidin capture (capture 1) of the biotinylated library reveals the percentage of available cysteines (B) M13-phage displayed thiol library was modified with **DFS** (C) Left over cysteines were monitored by a second biotinylation with BIA followed by a streptavidin capture (capture 2) (D) The difference between capture 1 and 2 reveals the percentage of library modified by DFS.

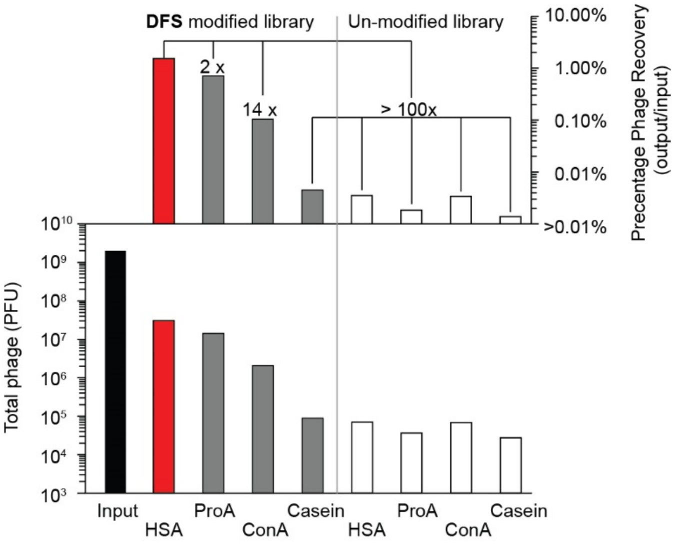

Supplementary Fig. 4. Phage recovery from the second selection campaign of round 3.
Input of Round 3 was bound to different proteins on protein-coated plates and recovery was calculated as PFU in output / PFU in input. phage displayed thiol library was modified with DFS

6. Panning strategy 3: panning in solution

This strategy was used for one round of panning. Magnetic streptavidin beads were blocked with blocking phage (note: blocking phage is a modified M13KE phage containing no sequences recognized by PCR primers; thus, it is not amplifiable with the PCR protocol used for phage libraries: see section S7) overnight and washed 3 times with 1 mL of PBS. **DFS** modified phage library (2×10^{11} pfu/mL) and blocking phage (2×10^{12} pfu/mL) were mixed in a 1:10 ratio and incubated with blocked streptavidin beads for 30 min to deplete beads binders. The depleted DFS-modified phage library was incubated with 5 μg of biotinylated HSA and T4GP-His_6 in a total volume 100 μL in PBS with 2% milk for 30 min at RT. In parallel, the depleted DFS-modified phage library was incubated with 5 μg of ConA-Bio in a total volume 100 μL in PBS with 2% milk for 30 min at RT. After incubation, 25 μL of blocked magnetic streptavidin beads were added to each mixture and incubated for 20 min at RT. The beads were captured with a magnetic rack and were washed nine times with 1 mL of PBS. The beads were then resuspended in 1 mL PBS and incubated for 30 min. The beads were captured with a magnetic rack, the supernatant was discarded, and the beads were resuspended with 60 μL mixture containing 1:1 hexane and DNAse-free water and shaken for 15 min (1500 RPM). The samples were heated to 55 °C for 10 min or until hexane completely evaporated. The remaining aqueous layer was collected, and PCR amplified for deep sequencing by Illumina Next seq.

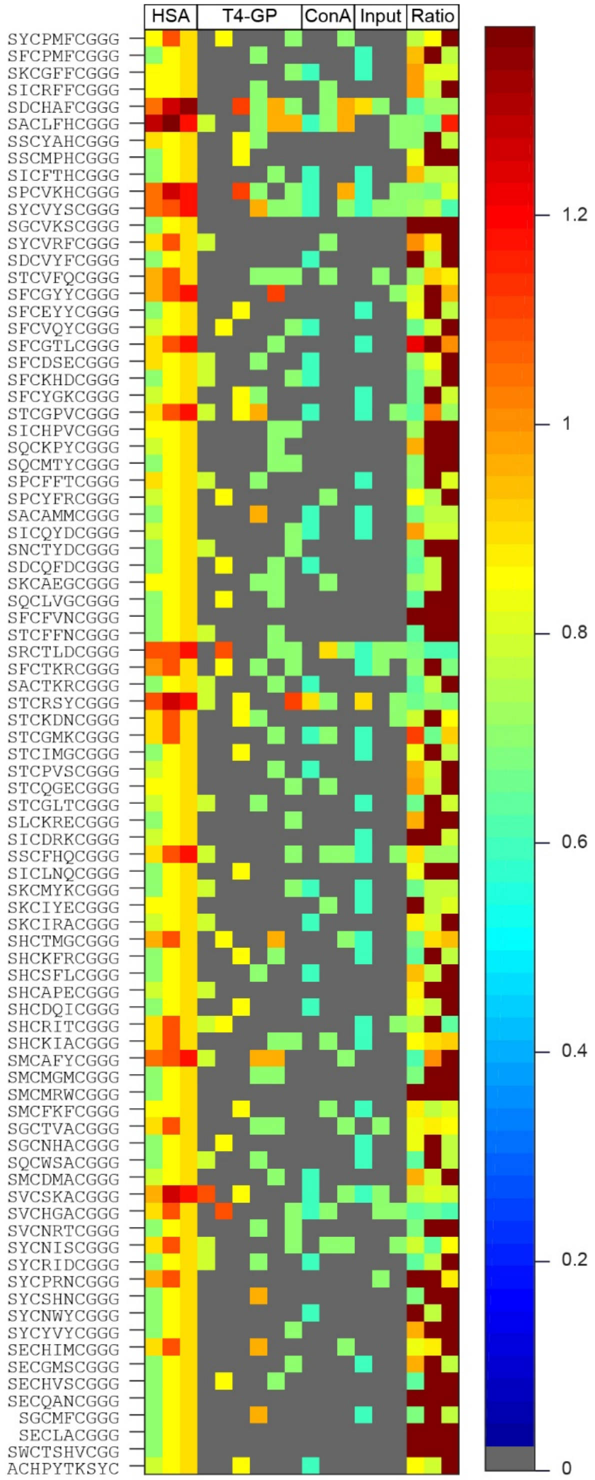

Supplementary Fig. 5. A heat map of 85 hits discovered from the third panning campaign. The sequences were enriched greater or equal to 4-fold (R>3, p=0.05) when compared to T4-GP and ConA.

7. General protocol for protein biotinylation

Protein was dissolved to 1 mg/mL in PBS at pH 7.4. 5-fold molar excess of EZ-Link Sulfo-NHS-Biotin (Thermofisher, cat# 21217) were added to the protein solution. The reaction mixture was incubated O/N at 4 °C. The next day, the protein was dialyzed 3 times in 4 L of PBS at pH 7.4. The biotinylated protein was captured with magnetic streptavidin beads to confirm biotinylation.

8. PCR amplification protocol for Illumina deep sequencing

25 µL of eluted or amplified phage solution was used as a template for PCR with a total volume of 50 µL. A typical 50 µL PCR mixture contains:

10 µL	5× Phusion buffer
10 µL	10 mM dNTPs
0.5 µL	Phusion® High-Fidelity DNA Polymerase (NEB, cat# M0530S)
2.5 µL	Forward primer 3'-CAAGCAGAAGACGGCATACGAGATCGGTCTCGGCATTC CTGCTGAACCGCTCTTCCGATCTXXXXCCTTTCTATTCTCACTCT-5', 10 µM
2.5 µL	Reverse primer 3'-AATGATACGGCGACCACCGAGATCTACACTCTTTCCC TACACGACGCTCTTCCGATCTXXXXACAGTTTCGGCCGA-5', 10 µM*
25 µL	Template solution (phage DNA)
8.5 µL	Nuclease free water

Thermocycler was performed using the following setting:

a) 95 °C for 30 sec
b) 95 °C for 30 sec
c) 60.5 °C for 15 sec
d) 72 °C for 30 sec
e) Repeat steps b) to d) 25 times
f) 72 °C for 5 min
g) hold at 4°C

*In 4 and 5 XXXX are forward and reverse multiplexing barcodes sequences. See Supplementary Fig. 5 for details.

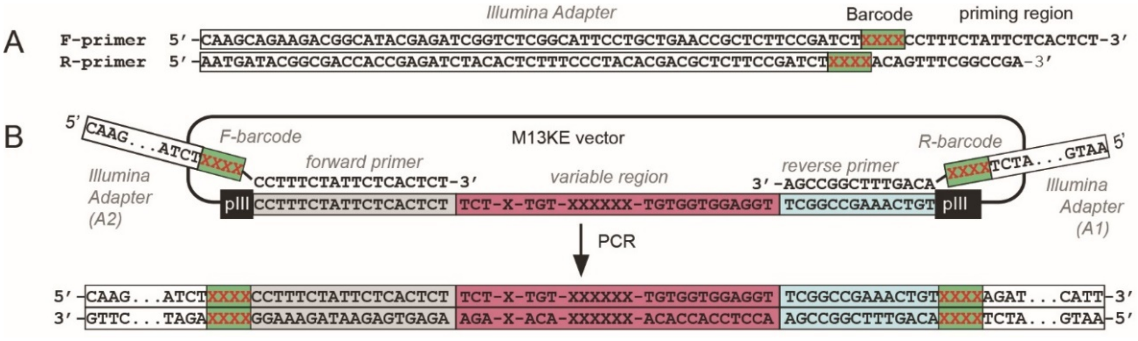

Supplementary Fig. 6. PCR amplification protocol for Illumina deep sequencing. (A) Primers used for amplifying phage DNA. XXXX denotes 4-nucleotide-long barcodes used to trace multiple samples in an Illumina sequencing experiment. (B) Generation of PCR product. Alignment of forward and reverse primers to 18-bp and 14-bp sequences flanking the variable region at the N-terminus of the pIII gene in M13KE vector, respectively.

9. Illumina sequencing of samples before and after panning

The DNA was produced by PCR as described in general PCR amplification protocol for Illumina deep sequencing with one exception: in amplification of libraries before panning (input), the template volume (phage solution) was 2 μL. All products were quantified by 2% (w/v) agarose gel in Tris-Borate-EDTA buffer at 100 volts for ~35 min using a low molecular weight DNA ladder as standard (NEB, cat# N3233S). PCR products that contain different indexing barcodes were pooled, allowing 10 ng of each product in the mixture. The mixture was purified by eGel, quantified by quBit and sequenced using the Illumina NextSeq paired-end 500/550 High Output Kit v2.5 (2×75 Cycles). Data were automatically uploaded to BaseSpace™ Sequence Hub. Processing of the data is described in section processing of Illumina data.

Processing of Illumina data

The Gzip compressed FASTQ files were downloaded from BaseSpace™ Sequence Hub. The files were converted into tables of DNA sequences and their counts per experiment. Briefly, FASTQ files were parsed based on unique multiplexing barcodes within the reads discarding any reads that contained a low-quality score. Mapping the forward (F) and reverse (R) barcoding regions, mapping of F and R priming regions allowing no more than one base substitution each and F-R read alignment allowing no mismatches between F and R reads yielded DNA sequences located between the priming regions as described in previous publications.[2] The files with DNA reads, raw counts, and mapped peptide modifications were uploaded to http://48hd.cloud/ server. Each experiment has a unique alphanumeric name and unique static URL in Tables S1-3.

For differential enrichment analysis, the test and control files were combined into one table and the file was processed using DE script described in section 31 below.

Supplementary Table 1: URL for 1st panning campaign deep sequencing results

	Modification	HSA	Protein A
R1	**DFS**	https://48hd.cloud/file/213	https://48hd.cloud/file/214
	none	https://48hd.cloud/file/217	https://48hd.cloud/file/218
R2	**DFS**	https://48hd.cloud/file/213	https://48hd.cloud/file/214
	none	https://48hd.cloud/file/217	https://48hd.cloud/file/218
R3	**DFS**	https://48hd.cloud/file/213	https://48hd.cloud/file/214
	none	https://48hd.cloud/file/217	https://48hd.cloud/file/218

Supplementary Table 2: URL for 2nd panning campaign deep sequencing results

	Modification	HSA	Protein A
R1	**DFS**	https://48hd.cloud/file/236	https://48hd.cloud/file/237
	none	https://48hd.cloud/file/421	N/A
R2	**DFS**	https://48hd.cloud/file/236	https://48hd.cloud/file/237
	none	https://48hd.cloud/file/421	N/A
R3	**DFS**	https://48hd.cloud/file/236	https://48hd.cloud/file/237
	none	https://48hd.cloud/file/421	N/A

Supplementary Table 3: URL for 3rd panning campaign deep sequencing results

	HSA	T4-GP	ConA
Input + **DFS**	https://48hd.cloud/file/799	https://48hd.cloud/file/799	https://48hd.cloud/file/799
Elution	https://48hd.cloud/file/798	https://48hd.cloud/file/797	https://48hd.cloud/file/796

10. General chemistry methods

LC–MS analysis of peptide modifications was obtained on Agilent Technologies 6130 LC–MS. A gradient of solvent A (MQ water) and solvent B (MeCN/H_2O 95/5) was run at a flow rate of 0.5 mL/min (0-4.0 min 5% B; 4.0-5.0 min 5%→60% B; 5.0-5.5 min 60%→100% B; 5.5-7.5 100% B, 7.5-11 min 100%→5% B).

11. Peptide synthesis

Peptides were synthesized on a PreludeX peptide synthesizer (Gyros Protein Technolgoes) by standard Fmoc solid chemistry using Rink Amide AM resin. Exception: in peptides with C-terminal propargyl-glycine **5h**, the first amino-acid was loaded manualy. Fmoc-protected amino acids, HBTU, Rink Amide AM resin were purchased from ChemPrep, Wellington FL USA. Peptides were cleaved from the resin by using a TFA/EDT/TIPS/Water (89.9/2.28/4.54/2.28 v/v) or TFA/Thioanisole/1,2-ethanedithiol/Anisole (90/5/3/2 v/v) for peptides with C-terminal propargyl-glycine. Cleaved peptiders were precipitated and washed with ice-cold diethyl ether, and further purified by HPLC and lyophilized into the product.

12. General protocol for cyclization with decafluorodiphenylsulfone

Procedure was analogous to previously published methods[4,5]. In short, linear peptide (10 mM) was dissolved in 50% acetonitrile and Tris buffer (50 mM Tris-HCl, pH 8.5), then 2 equivalents of **DFS** in 50% acetonitrile and Tris buffer (50 mM Tris-HCl, pH 8.5) was added to the mixture. The mixture was vortexed for 30 sec, incubated for 2 h at room temperature, purified by HPLC, and lyophilized to yield product as a powder.

13. General protocol for cyclization with pentaflurophenyl-sulfide

Procedure was analogous to previously published methods[4,5]. In short, linear peptide (10 mM) was dissolved in 50 mM Tris in DMF or in 50 mM $NaHCO_3$ in ACN:H_2O (for **1c**-**27c**), then 2 equivalents of **PFS** were added to the mixture. The mixture was vortexed for 30 sec and allow to react for 2-4 h at RT. The reaction mixture was purified by HPLC and lyophilized to yield the product.

14. General protocol for cyclization with hexafluorobenzene (HFB)

Procedure was analogous to previously published methods[4,5]. In short, linear peptide **14a** (10 mM) was dissolved in 50 mM Tris in DMF or in 50 mM $NaHCO_3$ in ACN:H_2O, then 1 equivalents of **HFB** were added to the mixture. The mixture was vortexed for 30 sec and allow to react for 24 h at RT. The reaction mixture was purified by HPLC and lyophilized to yield the product.

15. General protocol for cyclization with decafluorobiphenyl (DFB)

Procedure was analogous to previously published methods[4,5]. In short, linear peptide **14a** (10 mM) was dissolved in 50 mM Tris in DMF or in 50 mM $NaHCO_3$ in ACN:H_2O, then 1 equivalents of **DFB** were added to the mixture. The mixture was vortexed for 30 sec

and allow to react for 24 h at RT. The reaction mixture was purified by HPLC and lyophilized to yield the product.

16. Synthesis of N-terminally PEG_6 labeled peptides

PFS stapled peptides were dissolved in 1×PBS, 50% acetonitrile, then 1.5 eq of PEG_6-NHS ester (100 mg/mL, genereted *in situ*) was added to the solution. The mixture was incubated for O/N at room temperature. The reaction mixture was purified by HPLC and lyophilized to yield the product.

17. Synthesis of N-terminally Bodipy labeled peptides

N-terminal Fmoc-protected **PFS** stapled peptides were dissolved in 1:1 (v/v) PBS: acetonitrile, then 1.5 eq of BODIPY-NHS ester (100 mg/mL DMSO) was added to the solution. The mixture was incubated for O/N at room temperature. Pipperidine was added to a final concentration of 20% v/v for 30 min to deprotect the N-terminus Fmoc protecting group. The reaction mixture was purified by HPLC and lyophilized to yield the product.

18. Cysteine alkylation of peptides with iodoacetamide (IAA)

A linear peptide (0.005 mmol for 14a or 0.009 mmol for 21a) was dissolved in 50 mM Tris in ACN: H2O, then iodoacetamide (1.7mg, 2 eq, 0.009 mmol, 100mM stock solution for 14a or 3.3 mg, 2 eq, 0.018 mmol, 100 mM stock solution for 21a) was added to the mixture. The mixture was vortexed for 2 min and allowed to react for 4 h at rt. The reaction mixture was purified by HPLC and lyophilized to yield 14d (3.4 mg, 57%) and 21d (3.5 mg, 30%).

19. Formation of disulfide bridges on peptides

In a 50-mL polypropylene centrifuge tube, linear peptide (0.005 mmol for **14a** or 0.009 mmol for **21a**) was dissolved in DMSO (8 mL), and to this, PBS (32 mL) was added. The reaction mixture was agitated on a shaker for 48 h at rt. After 48 h, the crude disulfide bridge-bearing peptide was purified by HPLC and lyophilized to yield **14d** (3.4 mg, 24%). and **21d** (3.5 mg, 12%).

20. Cyclization of peptides with MBX

Pure linear peptide (0.005 mmol for **14a** or 0.009 mmol for **21a**) was dissolved in H_2O/MeCN (1:1, 1 mg peptide/mL) in a 15-mL centrifuge tube and to it a solution of MBX (1.2 equiv, 100 mM stock solution in MeCN) was added. Tris-HCl buffer (500 µL, 500 mM, pH 8.5, final concentration of Tris-HCl buffer was 50 mM) was added into the tube. The mixture was vortexed for 30 s and then shaken at rt for 2 h. After 2 h, the reaction mixture was purified by HPLC and lyophilized to yield **14e** 3.4 mg (yield 55%) and **21e** 3.5 mg (yield 30%).

21. Apelin analogue synthesis

The synthesis and characterization of NMe17A2 have been previously described.1–3 Briefly, the synthesis of this peptide was carried out using 2-chlorotritylchloride resin (0.8

mmol/g loading), and manually incorporated the following amino acids (1.1 eq compared to resin loading) in the following order: Fmoc-BrF-OH, Fmoc-Aib-OH, Fmoc-Nle-OH, Fmoc-Pro-OH, Fmoc-Gly-OH, Fmoc-Lys(Boc)-OH, Fmoc-His(Trt)-OH, Fmoc-Ser(tBu)-OH, Fmoc-NMeLeu-OH, Fmoc-Arg(Pbf)-OH, Fmoc-Pro-OH, Fmoc-Arg(PbF)-OH, Fmoc-Gln(Trt)-OH, Fmoc-Arg(PbF)-OH, Fmoc-Arg(PbF)-OH, Fmoc-Phe-OH, and Fmoc-Lys(Boc)-OH. Completeness of each coupling step was checked with Matrix Assisted Laser Desorption/Ionization (MALDI) coupled with Time of Flight-Mass Spectrometry (TOF-MS).

22. N_3-PEG_6-Apelin analogue synthesis

The Fmoc-deprotected apelin peptide on resin (50.0 mg, 0.01 mmol) was suspended in 5 mL of DMF, and 76.0 mg of N_3-PEG_6-COOH (1.0 eq to NMe17A2 peptide), 27.0 mg HOBt (1.0 eq to NMe17A2 peptide), and 62 μL DIC (1.0 eq to NMe17A2 peptide) were subsequently added. The reaction was bubbled at room temperature for 24 h. The solution was filtered to collect the resin beads, and the peptide was subsequently cleaved from the resin with 95:2.5:2.5 TFA/TIPS/H_2O.

23. PFS-Apelin analogue synthesis[4]

The N3-PEG6-NMe17A2 (2.00 mg, 4.30 x 10-1 mM final concentration) and RFF macrocycle **20c** (2.00 mg, 7.60 x 10-1 mM final concentration) peptides were dissolved in a 1.8 mL solution of 1:4:4 t-BuOH/H2O/DMF. Separately, 12.6 μL of 100 mM THPTA ligand (Tris(2-hydroxypropyltriazoylmethyl)amine) in DMF (1.6 equiv to N3-PEG6-NMe17A2) and 8.0 μL of 100 mM CuSO4 (from CuSO4•5H2O) in water (1 eq to N3-PEG6-NMe17A2) were combined and then added to the peptide solution. Lastly, 39.1 μL of 100 mM aminoguanidine•HCl in water (5 equiv to N3-PEG6-NMe17A2) and 60.6 μL of 100 mM sodium ascorbate in water (8 equiv to N3-PEG6-NMe17A2) were added to the reaction mixture. The reaction was stirred at room temperature for 4 hours (completion confirmed by HPLC, MALDI, and Q-TOF).

24. ^{19}F NMR binding experiment

NMR experiments at the University of Minnesota were performed on a Bruker Avance III HD with a Prodigy TCI cryoprobe (2100:1 S/N for ^{19}F). **PFS**-peptides were tested as 20 μM solutions in experiments were performed with a fluorinated peptide concentration of 20 μM in 50 mM phosphate, 100 mM NaCl and 26.5 μM 2,2,2-trifluoroethanol, pH 7.4 with varying concentrations of rat or human serum albumin (from 0-160 μM). Parameters used for each experiment are as follows: 750 scans, acquisition time of 0.05 s, relaxation delay of 0.7 s, spectrum centered at -135 ppm with a sweep width of 20 ppm. A reference spectrum observing 2,2,2-trifluoroethanol was acquired for each sample with the following parameters: 16 scans, acquisition time of 0.5 s, relaxation delay of 1 s, spectrum centered at -75 with a sweep width of 10 ppm. The observed chemical shift of the reference was deducted from -77.75 ppm, and this difference was applied to the peptide spectrum.

Supplementary Table 4: A typical experiment involving titration with HSA.

Component	HSA	**PFS**-peptide	TFE	D_2O	PBS
Stock (μM)	2000	200	26.5	n.a	n.a
	0	20	2	25	423
	2.5	20	2	25	420.5
	5	20	2	25	418
	10	20	2	25	413
	20	20	2	25	403
	40	20	2	25	383

n.a: Not applicable

This series would produce a titration with 20 μM **PFS**-peptide held constant and the HSA varying in a two-fold fashion: 0, 10, 20, 40, 80,160 μM. TFE refers to 2,2,2-trifluoroethanol as a 1/1000 dilution (approx. 26.5 μM), and PBS components were 50 mM phosphate, 100 mM NaCl, pH 7.4.

NMR experiments conducted at the University of Alberta (Supplementary Fig. 10-11) were performed on an Agilent MR400 with an OneNMR™ Probe (690:1 S/N for ^{19}F). **PFS** and **OFS** peptides were tested as 50 μM solutions in PBS, 2.5% DMSO, and 10% D_2O with 100 μM HSA. Parameters used for each experiment are as follows: 2048 scans, acquisition time of 0.15s, relaxation delay of 0.85s, spectrum was centered between **PFS** and **OFS** fluorine peaks and with a sweep width of 207 ppm. Peptide spectra were referenced to TFA at -76.55 ppm.

25. Fluorescence polarization binding assay

Black 384 well plates (PerkinElmer, cat# 6007270) were used to measure all binding assays. The fluorescent-labeled peptides were dissolved to 10 mM in DMSO and diluted to 20 μM in DMSO for use. Each well contained 19 μL of HSA in PBSwith the range of final concentration from 190 μM to 15 nM. 1 μL of 20 μM of fluorescentlly labeled peptide was added to the wells to a final concentration of 1 μM. Each measuring point was made in duplicate. Before measuring, the plate was centrifuged 500×g for 5 min at RT, incubated for 10 min, and shaken for 5 min in the dark. The measurement was performed in Cytation5 Cell Imaging Multi-Mode Reader from BioTek. The data were analyzed and processed in OrginLab.

26. Isothermal titration calorimetry (ITC) binding assay

Titration experiments were performed using a Microcal VP-ITC instrument. Pepties were disolved in PBS pH 7.4 to a final concentration of 400 μM . In the case where ligands have poor solubility in the buffer, up to 5% (v/v) DMF was used. The HSA solution was prepared with the identical buffer as the peptides to a final concentration of 40 μM. All solutions were degassed with MicroCal ThermoVac. All the titration in this study was carried out at 37 °C with stirring at 300 rpm. An initial injection of 2 μL followed by a total of 41 injections of 10 μL peptide solution was added over the interval of 4 min into the HSA solution. The data were evaluated using the MicroCal™ Origin™ Version 5.0. The heat signals were fitted to "one set of sites" or "two sets of sites" models to obtain the binding enthalpy, affinity, and stoichiometry estimates.

27. *In vivo* pharmacokinetic experiment

All the procedures and experiments involving animals were carried out using a protocol approved by the Health Sciences Laboratory Animal Services (HSLAS), University of Alberta. The protocol was approved as per the Canadian Council on Animal Care (CCAC) guidelines. Approved protocol # AUP00002467. All mice (Strain: C57BL/6J) were maintained in pathogen-free conditions at the University of Alberta breeding facility. Housed on ventilated caging (Tecniplast) ; Temperature: 21 +/- 2 degrees Celcius; Humidity: 30-70% rH ; Light Cycle: 12L:12D. Caging: 1 cm of aspen chip, 1 cotton Nestlet, 1 Bed'r'nest, 1 polycarbonate tube; Water: ad lib, deionized UV filtered water; Food: ad lib, irradiated Lab Diet 50LD Rodent Chow (4.5% fat). All mice were maintained in pathogen-free conditions at the University of Alberta breeding facility. Peptide mixtures of 100 μM were prepared in PBS. Mice were administered with 200 μL of the peptide mixture solution with tail veil injection. A series of 6 blood samples were collected at time points from 2 min up to 240 min. Samples were collected in tubes that contained sodium citrate as an anticoagulant and then centrifuged at 5 min at 2,000×g to collect the blood plasma. 10 μL of plasma portion were transferred into a tube containing 40 μL of 8:2 acetonitrile/water to precipitate proteins. The samples were centrifuged at max speed for 10 min at 4 °C. Supernatants were then transferred to new tubes and subjected to analysis by LC-MS.

28. LC–MS analysis for pharmacokinetics

LC–MS studies of the stability of peptides in mice were performed in Hewlett Packard 1100 series instrument using a Phenomenex Jupiter C4 protein column (300 Å, 2x50 mm, 0.3 mL/min, A: 0.1% formic acid in water, B: 0.1% formic acid in acetonitrile (0 min 2% B, 0-10 min 2%-70% B, 10-15 min 70% B, 15-20 min 70%-2% B). The amount of peptide

remaining was calculated with the area under the curve of SIM (Selected Ion Monitoring) peak in LC–MS.

29. Docking Calculations

Docking of **PFS**-SICRFFCGGG macrocycle to the human serum albumin (HSA) protein was performed using five different HSA crystal structures obtained from the RCSB databank (PDB IDs: 1e7e [https://www.rcsb.org/structure/1E7E], 1e7f [https://www.rcsb.org/structure/1E7F], 1e7g [https://www.rcsb.org/structure/1E7G], 1e7h [https://www.rcsb.org/structure/1E7H], 1e7i [https://www.rcsb.org/structure/1E7I]).[6] In these crystal structures, multiple fatty acids (between six and nine) of different lengths are bound to HSA proteins. The previously characterized binding locations of the fatty acids on the HSA surface[6] are here called binding sites, labeled by distinct indices. In our calculations, we docked **PFS**-SICRFFCGGG macrocycles to each of those binding sites. The **PFS**-SICRFFCGGG structure was constructed with ChemSketch.[2] Both **PFS**-SICRFFCGGG and HSA were converted to Autodock Vina[7] readable format using MGL Tools. In docking the **PFS**-SICRFFCGGG to HSA, the grid box center locations were selected to coincide with the centers of mass of the bound fatty acids. The grid boxes had 2.6 x 2.6 x 2.6 nm^2 dimensions with a default spacing of 0.0375 nm. The Autodock Vina configuration files and the docking run procedure were generated and ran automatically with a bash script provided by us on Github (https://github.com/vukoviclab/hsa-dock). The docking runs were performed using three different random seeds.

Supplementary Fig. 22A shows the HSA protein with some bound fatty acids, based on the PDB ID: 1e7e[6] [https://www.rcsb.org/structure/1E7E]. Overlaid with this structure is **14c** docked to the corresponding fatty acid binding sites on the HSA surface. Supplementary Fig. 22B shows the binding scores for **14c**–HSA complexes across different HSA structures based on the distinct pdbIDs and binding site locations (complete results summarized in Supplementary Fig. 23 and Supplementary Table 6). The docking results (Supplementary Fig. 22B) suggest that the primary HSA binding site for **14c** is binding site 1. The next most favorable binding sites are sites 8, 6, and 7, with the most favorable binding scores of –6.6 ± 1.1 kcal/mol, –6.2 ± 0.7 kcal/mol, and –6.0 ± 1.2 kcal/mol, respectively (Supplementary Fig. 22**,** Supplementary Table 6). We observed binding sites 1 and 8 are near to each other on the HSA surface, with the center of mass distance between fatty acids occupying these sites being 5.3 Å. As such, it is unlikely that binding sites 1 and 8 can be simultaneously occupied by two **14c** molecules. Supplementary Fig. 22A shows the four HSA residues interacting with the fatty acid in binding site 1 via charge and nonpolar interactions. In contrast, **PFS**-SICRFFCGGG (**14c**) has more interactions with this HSA binding site, including the HSA residues R114, R117, Y138, Y161, I142, L154, S193. Notably, HSA residues R117, Y138, and Y161 in binding site 1 are found to mediate HSA interactions with both the fatty acid and **14c**. Additionally, we examined the superposition of **14c** and several ligands with established binding in the fatty binding site 1 (IB site).[8,9] By aligning our structures of 1**4c**-HSA complex, using the structure with the best docking score, and the ligand-HSA complex crystal structures, we identified four candidate ligands whose positions in FA1 IB site significantly overlap with **14c** (Supplementary Tables 7-8, Supplementary Fig. 24).

Structure of HSA bound to diclofenac[7] (PDB ID: 4z69 [https://www.rcsb.org/structure/4Z69], Supplementary Fig. 23B-D) contains two HSA chains in it. One of the HSA chains has single diclofenac at the binding site 7, while the second HSA chain has three bound diclofenac ligands in total, with two also located at the binding site 7, and the third located near the binding site 1, which is also occupied by a bound fatty acid. The structure locations suggest that diclofenac has the strongest binding to site 7, since it is observed there in both HSA chains, and a weaker binding to binding site 1, as only one single HSA chain, is observed with diclofenac nearby.

When examining the superposition of diclofenac (crystal structure) with **14c** (docking) in IB site of HSA. The overlap of **14c** and diclofenac, occurs only on one side of **14c**. **14c** binds to 19 amino acids of HSA, and diclofenac binds to 9 amino acids of HSA, and 7 out of 9 amino acids which bind to diclofenac also bind to **14c** (L154, G189, Y161, K190, R186, H146, I142). Furthermore, IB site of HSA in the crystal structure 4z69 [https://www.rcsb.org/structure/4Z69] binds simultaneously to both diclofenac and a fatty acid so that diclofenac binding could be modulated by the fatty acid binding. There is no fatty acid in the **14c**-HSA complex obtained by docking. Therefore, **14c** has a larger binding site to FA1 than diclofenac, and a more favorable docking score of -10 kcal/mol, compared to diclofenac's binding score of -6.6 kcal/mol (Supplementary Fig. 23D).

30. Docking simulations for free energy calculations of 14c and alanine scans

We chose initial binding poses from docking simulation with Autodock-VINA at 5 different binding pockets. The binding pocket was defined by 30x30x30 $Å^3$ cubic box whose center is at the center of mass of the binding ligand in albumin-fatty-acid (PDB ID: 1e7f [https://www.rcsb.org/structure/1E7F], 1e7i [https://www.rcsb.org/structure/1E7I])[6] or albumin-hemin (PDB ID: 1o9x [https://www.rcsb.org/structure/1O9X])[10] complexes. We located our peptide at the center of each binding pocket and performed virtual screening to find the lowest energy binding conformation of the peptide with Autodock-VINA forcefield.

For further equilibration, we conducted NVT and NPT simulations at 298 K in explicit water molecules with the conformations generated by the procedures described above. In detail: the protein-peptide complex was solvated with explicit TIP3P water molecules in a 120×120×120 $Å^3$ box. Then, the system was neutralized and ionized with explicit sodium and chloride ions at 150 mM concentration. After energy minimization, we ran NVT simulations for 2 ns and NPT for 10 ns at 1atm. Throughout all equilibrium simulations, the equation of motion was integrated with a general leap-frog scheme with 2 fs timesteps. Electrostatic interaction was calculated with Particle-Mesh Ewald (PME) method[11].

With these equilibrated binding conformations, we calculated binding free energy using steered molecule dynamics (SMD) and umbrella sampling technique[12]. For SMD, we applied varying force on the peptide to pull the center of mass of the peptide at the constant speed out of the binding pocket until it is completely detached from the protein (about 4 nM away from the original position). The pulling force profile is shown in Supplementary Fig. 24. The conformation was sampled at every 50 ps with 0.5 angstrom interval and used 70 configurations as initial conformations for umbrella sampling simulation windows.

Finally, potential of mean force (PMF) of the unbinding process was assessed through umbrella sampling simulations. In each simulation window, we ran biased molecular dynamics simulations with a harmonic potential whose interaction center is located at the specific distance between the binding pocket and the center of mass of the peptide. Total simulation time was 20 ns per window with 2 fs timesteps. The force coefficient for the umbrella sampling simulations was as large as 1000.0 $kJ \cdot mol^{-1} \cdot nm^{-2}$ to maintain the distance between the pocket and the peptide. We collected pulling forces for each window and at last analyzed the data with Weighted Histogram Analysis Method[13] (WHAM) with 200 bins. The data before 5ns was ignored so a total of 15 ns data was considered. All simulations were done with GROMACS 2019[14].

The change of free energy of binding by alanine substitutions were estimated by free energy perturbation (FEP) method[15-17]. Briefly, our procedures are as follows. We took initial conformations from docked structures and solvated in a periodic box with 1.5nm margin. Then, the system was equilibrated under NVT condition at 298K for 5ns after energy minimization. And 10ns NPT equilibration at 298K and 1atm followed. Finally, the production runs were conducted in 28 λ-windows for 500ps with respect to both the albumin-bound and albumin-free conformations. The λ-schedule is presented in Supplementary Table 9. Soft-core potential was used to avoid crash during FEP and electrostatic potential was computed with Potential Mesh Ewald (PME) scheme. Regarding R4A mutations, $\Delta\Delta G$ should be calculated with extra care because it accompanies the variation of the peptide charge, and it is well-known that it may lead to very large discrepancy with experimental results[18]. Therefore, we extended the simulation time to 5ns in R4A case so that 10 times more samples were collected and analyzed. We adopted CHARMM36[19] force field for the protein and peptide, and Charmm-modified TIP3P models for water. All simulations were carried out with GROMACS 2019[14].

The histograms of the distances from the center of mass of the peptide to albumin binding pockets for all umbrella sampling simulations are shown in Supplementary Fig. 25. The distributions well cover whole range of the distance (0-3.7nm), which indicates that the simulation windows and the constraint force constant were adequately set to sample all possible conformations. The calculated PMFs of three binding modes are presented in Supplementary Fig. 26. Note that in the plot we aligned the y-values of the plots to make energy minima to be zero for comparison and calculated the energy differences from the bottom to the average PMF over the points where the distance is greater that 10 Å as binding free energies. Our plot shows pocket 1, which is proximate to the hemin binding site, has significantly stronger binding energy than the others. The ΔG was about -7.0 kcal/mol for pocket 1, while the others exhibit weaker binding affinity with less than -4.2 kcal/mol of ΔG. These results are consistent with our assumptions and experimental observations that the binding site of the peptide is proximate to the hemin binding site and the peptide competes with hemin for the interactions.

To figure out the reason for strong binding affinity of pocket 1, we analyzed SMD and umbrella sampling trajectories. In the SMD simulations, ARG4 of the peptide forms stable salt bridges with ASP183 of the albumin (Fig. 5B). In contrast, it is absent in the other binding modes at around 9ns (Fig. 5C-D). Thus, we inferred that the salt bridge between these two amino acids may provide the additional stability the albumin-peptide complex. Since our interest was the intermediate binding regime where the displacement ranges from

6-10 Å and the PMF of the pocket 1 starts to deviate from the others, we performed hydrogen bond interaction searches with 3.5 Å cut off distance between a donor and an acceptor at intermediate regime from umbrella sampling simulations. These findings suggest that the interaction between ARG4 and ASP183 plays a crucial role in the binding of the peptide to the pocket 1 binding site of albumin. This was supported by the fact that hydrogen bonds between these residues were observed in over 90% of the simulation snapshots in pocket 1, while the other pockets showed them for no more than 10% of the simulation time (Supplementary Fig. 27). Overall, these findings provide important insights into the mechanism of binding between the peptide and albumin, which could inform the design of future albumin-binding peptides with improved affinity and specificity. The concurrence of the docking results and the experimental binding data provides strong evidence that our simulations accurately predicted the binding for **14c**.

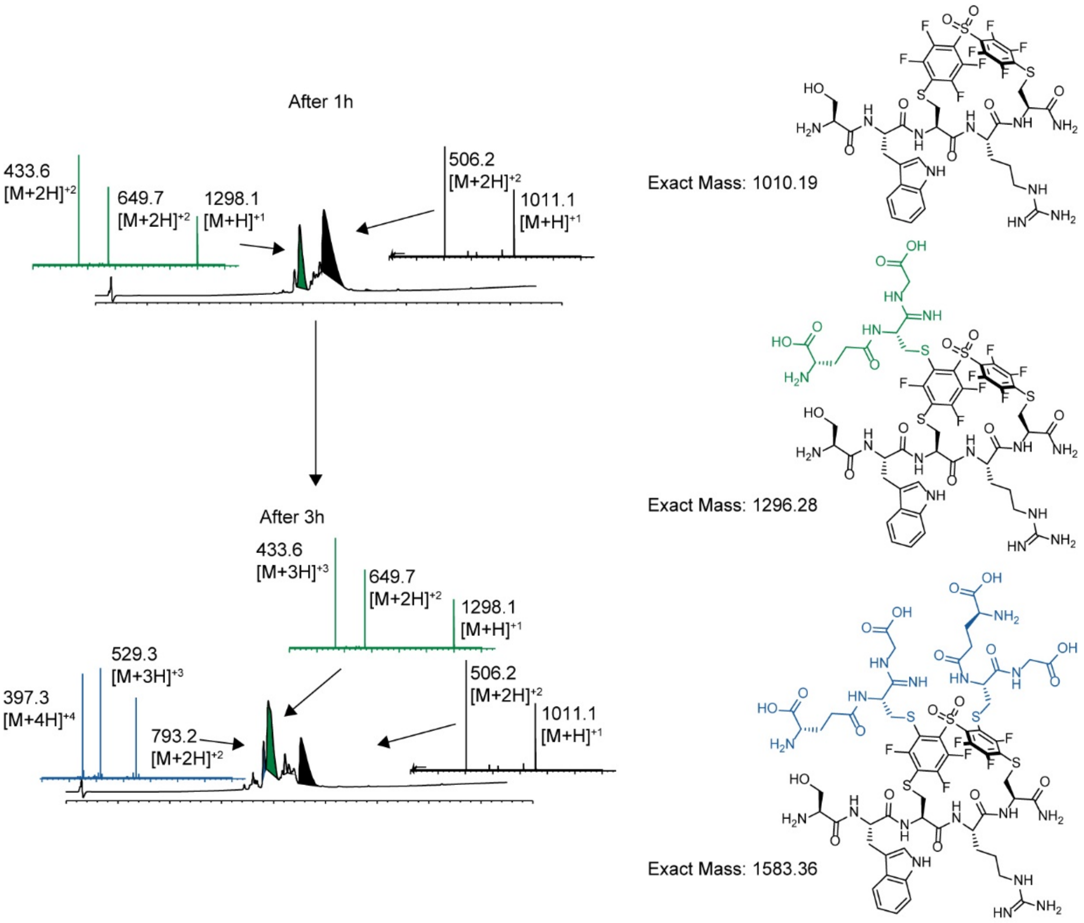

Supplementary Fig. 7. DFS stapled peptide reacted with GSH over 3 hours . The **OFS**-SWCRC peptide was added with one equivalent of GSH in 60% acetonitrile in 50 mM Tris-HCl at pH 8.5. The reaction was monitored by LC–MS.

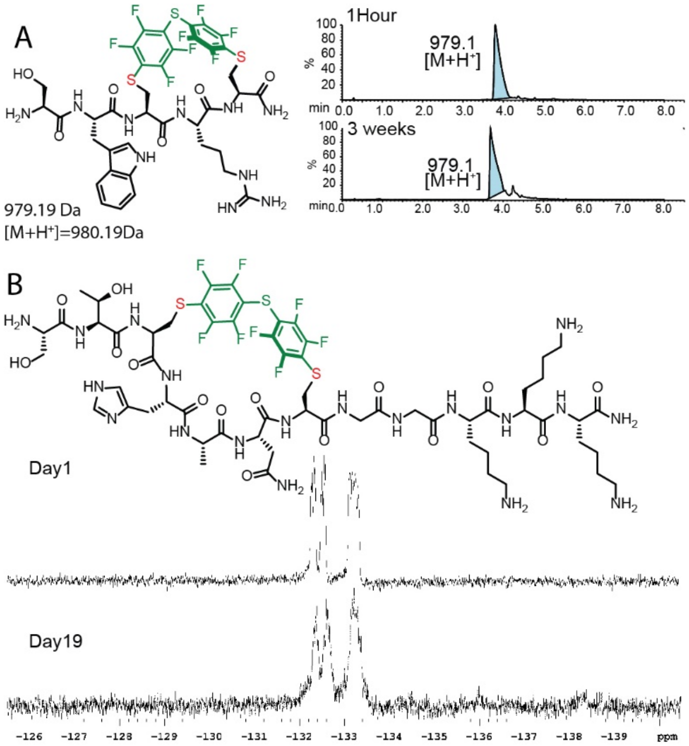

Supplementary Fig. 8. Stability of the **PFS** stapled peptides (A) Results of the **PFS**-SWCRC mixed with 2-mercaptoethanol and analyzed by LC–MS after 1 hour and 3 weeks. The **PFS**-SWCRC macrocycle was combined with 1 equivalent of 2-mercaptoethanol in 60% acetonitrile and 50 mM Tris-HCl at pH 8.5. The mixture was monitored by LC–MS. (B) A spectrum of 1 mg of **PFS**- STCHANCGGKKK mixed with 1 mg of HSA over 19 days in PBS. The mixture was monitored by ^{19}F NMR in PBS, 10% D_2O

Supplementary Table 5: Summary of the selected peptide sequences.

#	Sequence	**a**	**b**	**c**	**d**	**e**	**f**	**g**	**h**	**i**	**j**	**k**	**l**	**m**	**n**
		SH	DFS	PFS	PFS C-BODIPY	PFS N-BODIPY	DFS C-BODIPY	PFS + N-PEG7	PFS + C-term. triazole-PEG4	PFS + C-term. amide-PEG4	HFB	DFB	IAA	MBX	Apelin conjugate
1	STCHDITC	**	LS												
2	STCHYIGC	**	LS												
3	STCHANC	**	LS												
4	STCHTIYC	**	LS												
5	SICRFFC	**	LS												
6	SFCPMFC	**	LS												
7	SLCKREC	**	LS												
8	STCQGEC	**	LS												
9	STCHDITCGGKKK	**9a**	**9b**	**9c**											
10	STCHYIGCGGKKK	**10a**	**10b**	**10c**											
11	STCHANCGGG	**	**11b**	**11c**											
12	STCHANCGGKKK	**12a**	**12b**	**12c**											
13	STCHTIYCGGKKK	**13a**	**13b**	**13c**											
14	SICRFFCGGG	**14a**	**14b**	**14c**		**14e**		**14g**		**14i**	**14j**	**14k**	**14l**	**14m**	
15	SFCPMFCGGG	**	**15b**	**15c**											
16	SLCKRECGGG	**16a**	**16b**	**16c**											
17	STCQGECGGG	**17a**	**17b**	**17c**											
18	SICRFFCGGK	**			**18d**		**18f**								
19	STCQGECGGK	**			**19d**										
20	SICRFFCGGGZ	**		**20c**					**20h**						**20n**
21	AICRFFCGGG	**		**21c**											
22	SACRFFCGGG	**		**22c**											
23	SICAFFCGGG	**		**23c**											
24	SICRAFCGGG	**		**24c**											
25	SICRFACGGG	**		**25c**											
26	SICRFFCGGGK(N3)	**		**26c**											

The peptides nominated from three panning campaigns were chemically synthesized and modified. **Linear peptides were made as intermediates but not used in any assays and their characterization are not included. *LS= low solubility: further tests were abandoned

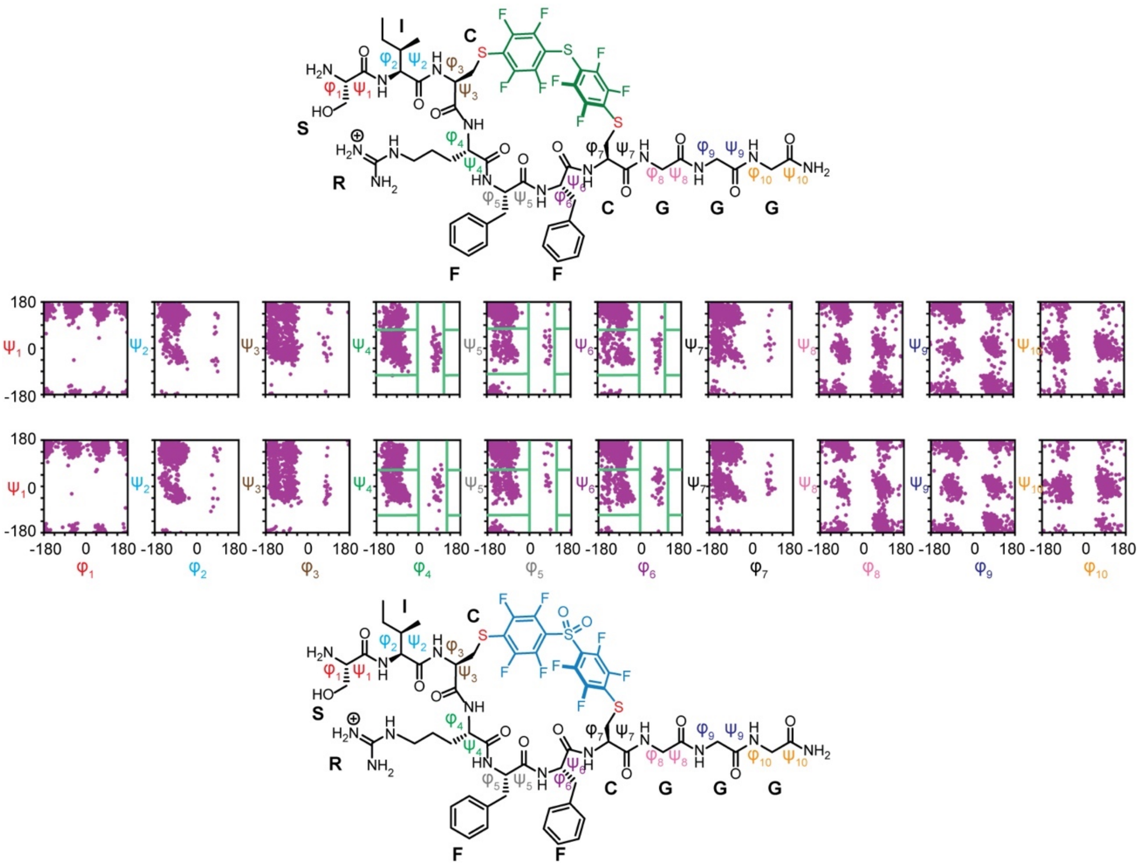

Supplementary Fig. 9. Ramachandran plot of the cyclic peptide backbone for **14b** and **14c** : Green lines indicate the binning boundaries used in the cluster analysis.

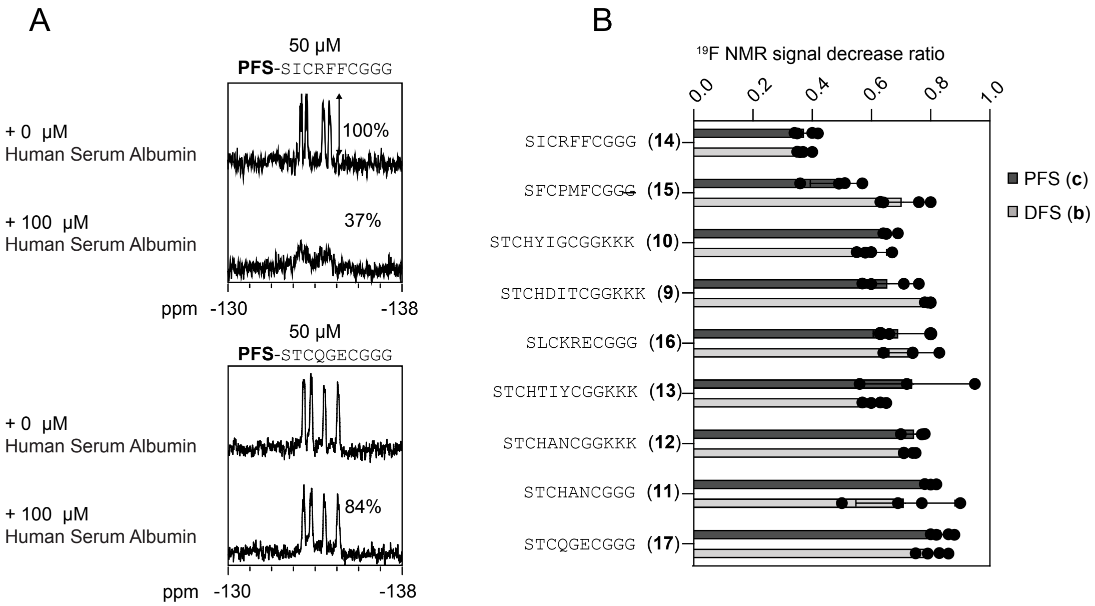

Supplementary Fig. 10. Summary of the ^{19}F NMR binding measurement. (A) HSA titration spectra of 50 μM of **9b-17b** and **9c-17c** against 100 μM of HSA. (B) The percentage represents the remaining peak intensity following the addition of HSA. Detailed NMR spectra for panel (B) are described in Supplementary Fig. 11.

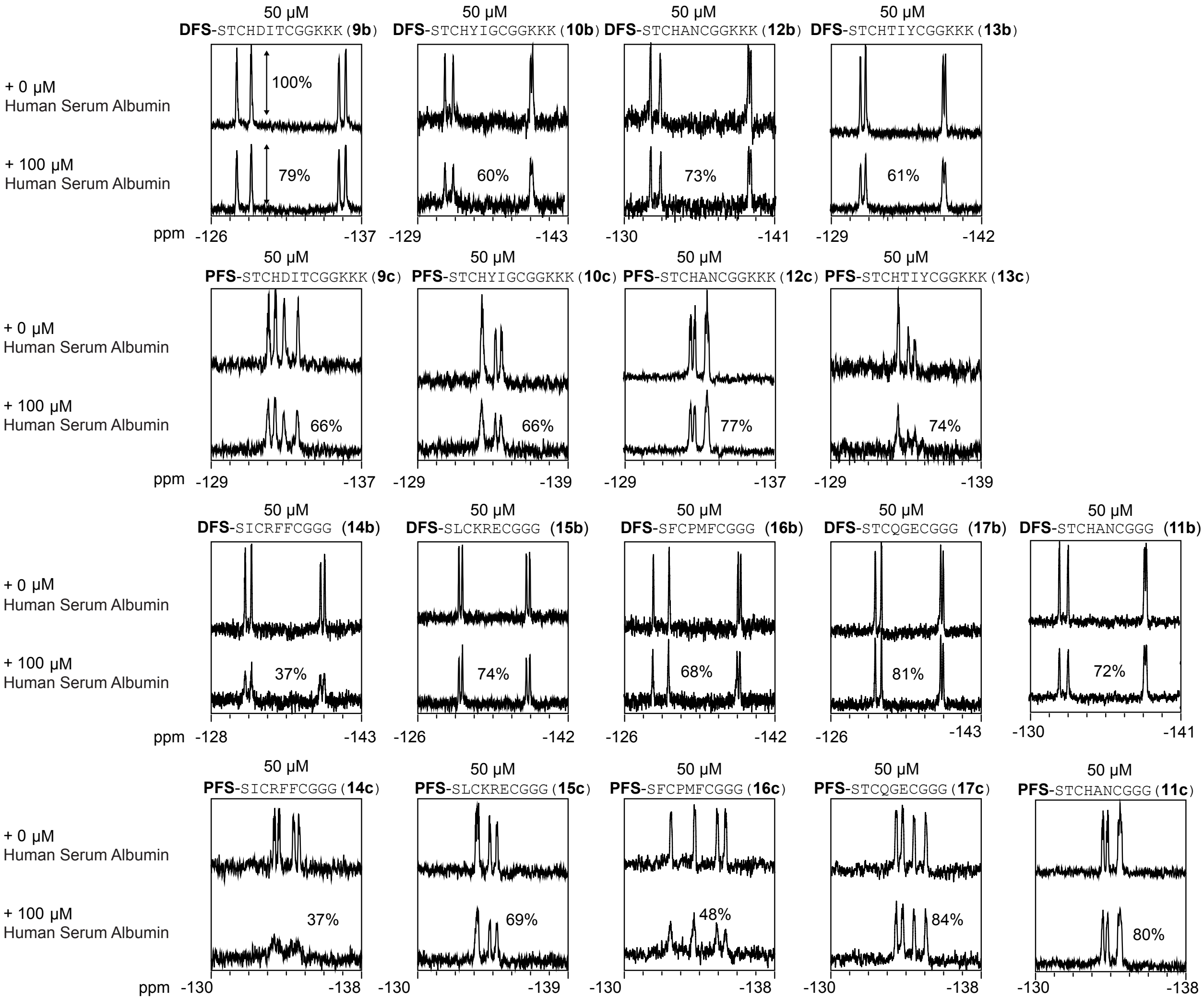

Supplementary Fig. 11. ^{19}F NMR measurement of binding of **9b-17b** and **9c-17c** to HSA. The percentage represents the peak height remaining after the addition of HSA. Measurements were performed on an Agilent MR400 with an OneNMRTM Probe (690:1 S/N for 19F). PFS and OFS peptides were tested as 50 µM solutions in PBS, 2.5% DMSO, and 10% D2O with 100 µM HSA

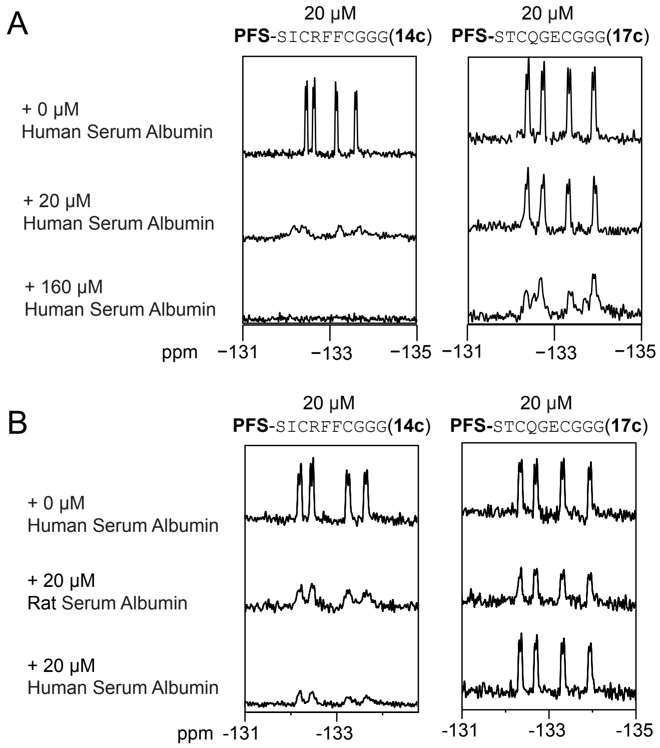

Supplementary Fig. 12. Binding of **14c** and **17c** to human and rat serum albumin.
(A) The ^{19}F NMR titration spectra for human serum albumin; (B) ^{19}F NMR measurement of binding of **14c** and **17c** to rat and human serum albumin, all reagents at 20 μM concentration.

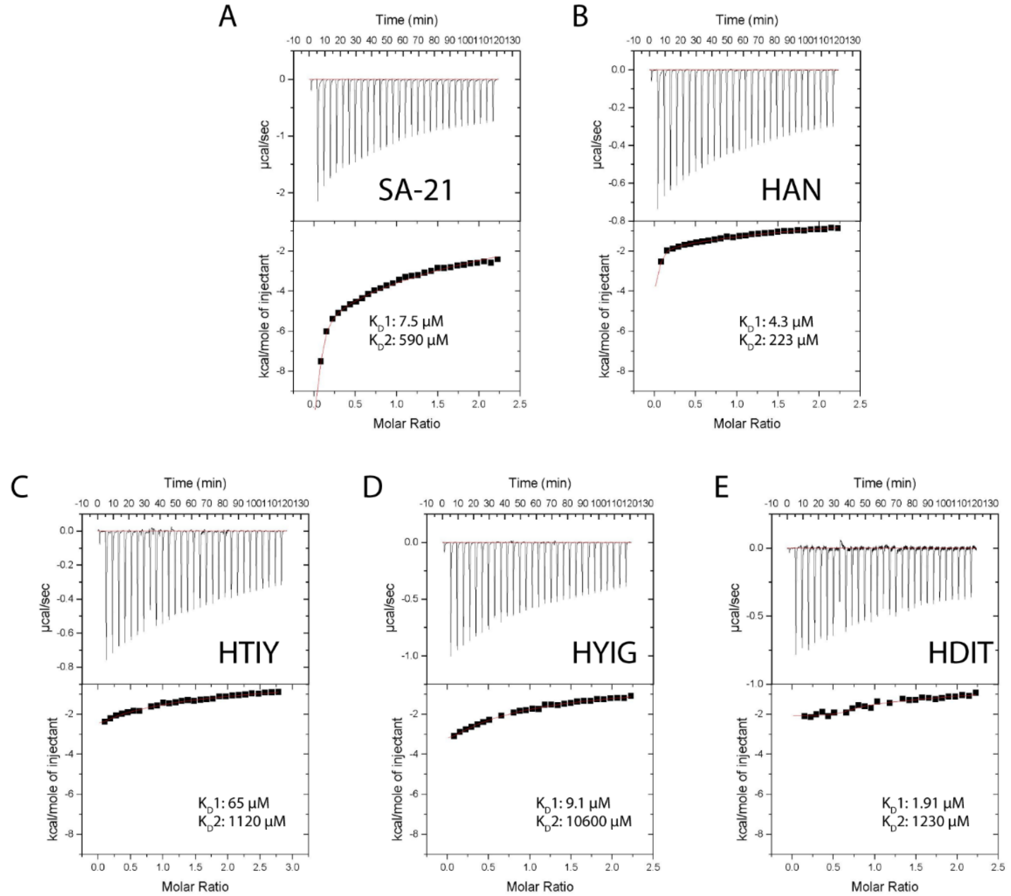

Supplementary Fig. 13. ITC experiment with 4 mM peptide and 0.4 mM albumin in PBS The ITC traces and binding isotherms for peptides (A) SA-21, (B) **DFS**-STCHANCGGKKK (**12b**), (C) **DFS**-STCHTIYCGGKKK (**13b**), (D) **DFS**-STCHYIGCGGKKK (**10b**) , (E) **DFS**-STCHDITCGGKKK (**9b**).

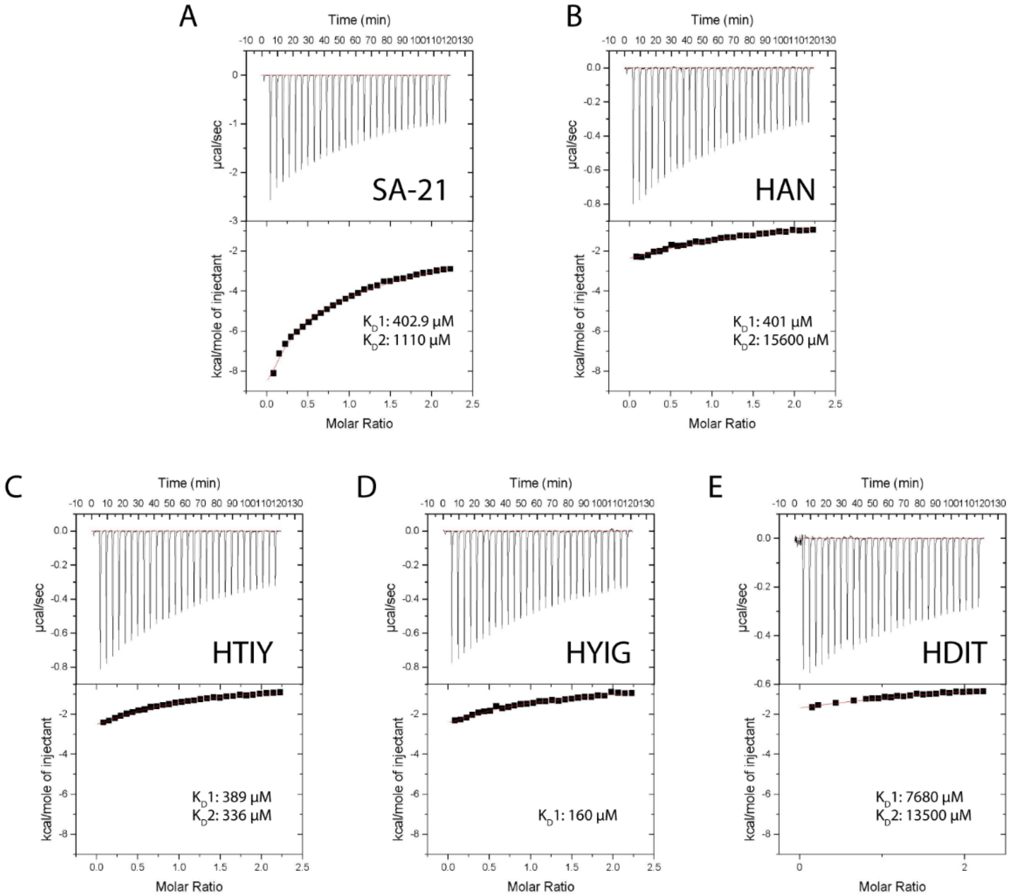

Supplementary Fig. 14. ITC experiment with 1 mM peptide and 0.1 mM albumin in PBS. The ITC traces and binding isotherms for peptides (A) SA-21, (B) **DFS**-STCHANCGGKKK (**12b**), (C) **DFS**-STCHTIYCGGKKK (**13b**), (D) **DFS**-STCHYIGCGGKKK (**10b**) , (E) **DFS**-STCHDITCGGKKK (**9b**).

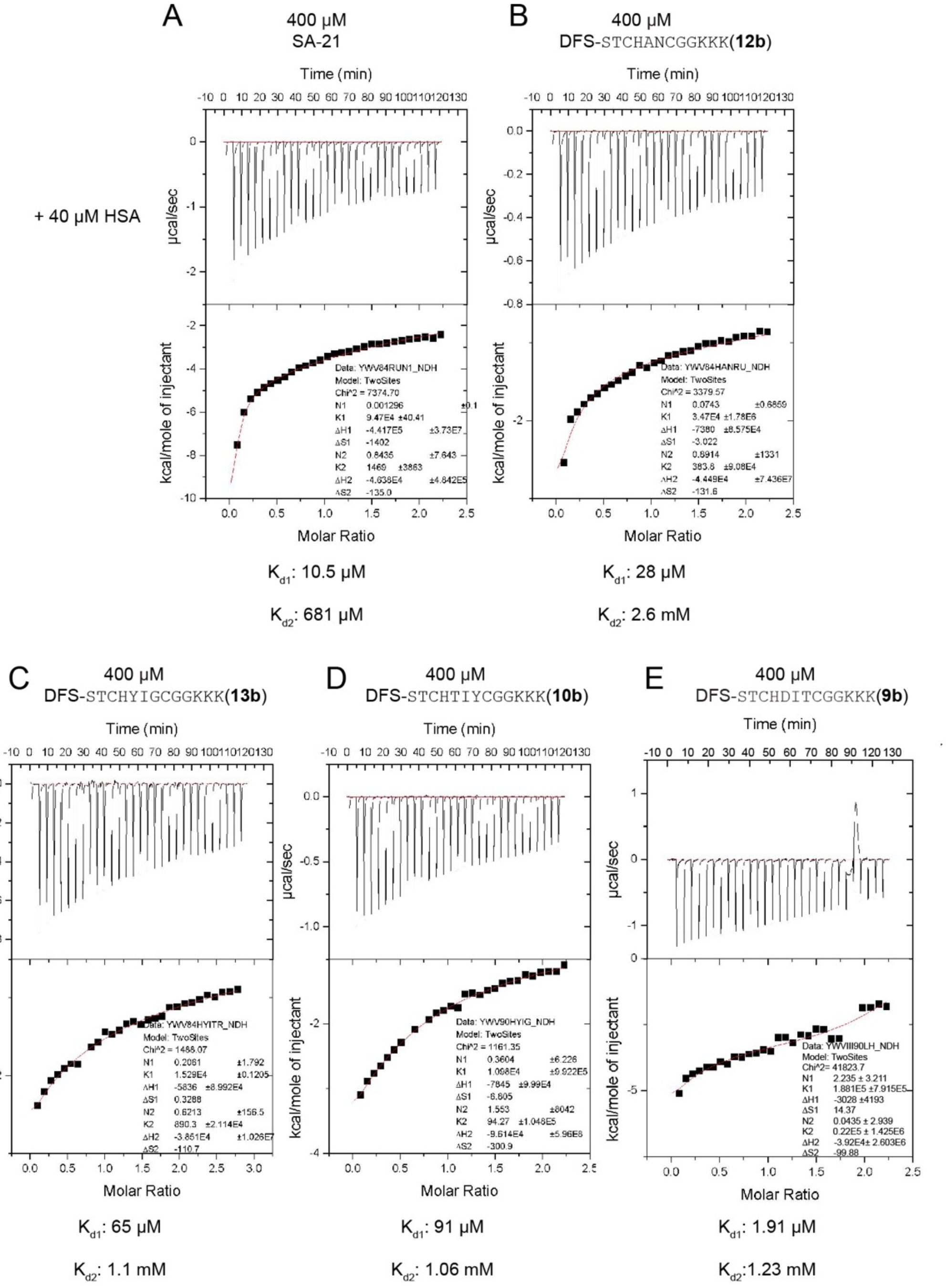

Supplementary Fig. 15. ITC experiment with 1 mM peptide and 0.1 mM albumin in PBS.
The ITC traces and binding isotherms for peptides (A) SA-21, (B) **DFS**-STCHANCGGKKK (**12b**), (C) **DFS**-STCHYIGCGGKKK (**13b**), (D) **DFS**-STCHTIYCGGKKK (**10b**) , (E) **DFS**-STCHDITCGGKKK (**9b**).

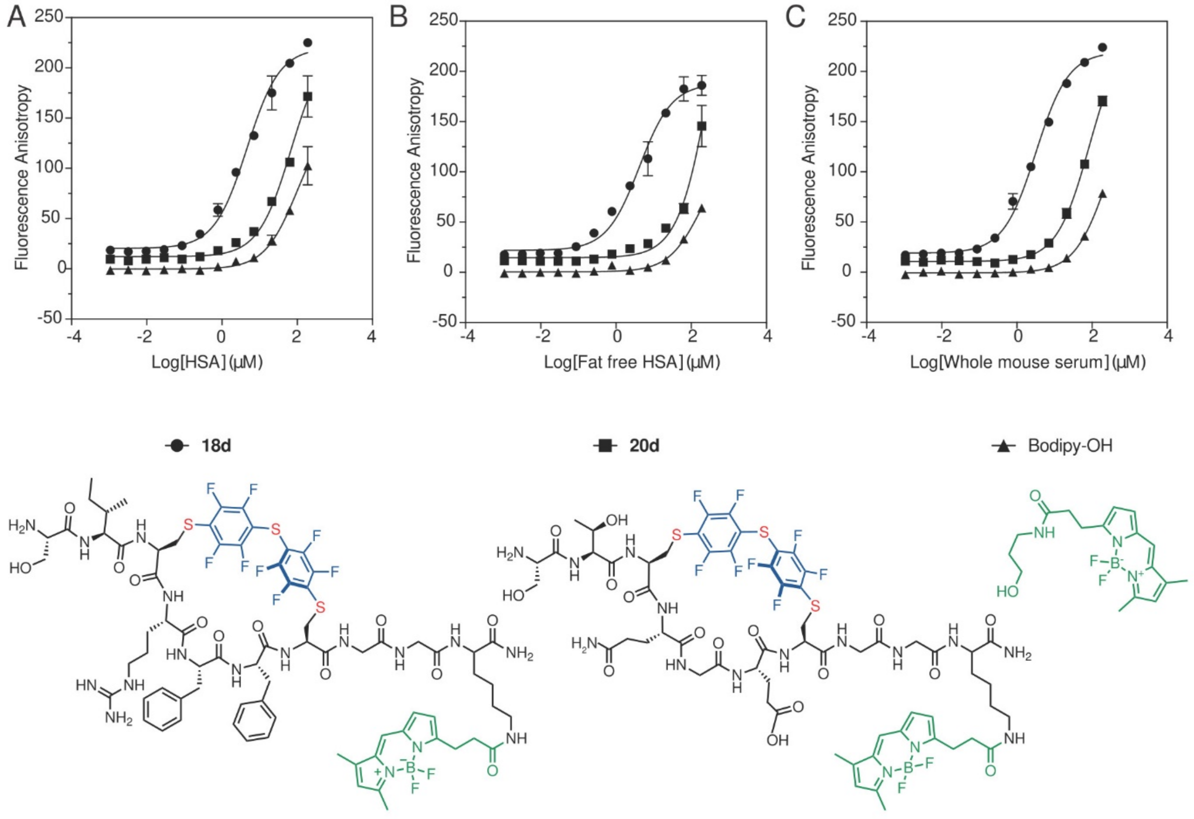

Supplementary Fig. 16. FP binding assay between albumin and peptide-BODIPY.
(A) FP assay for BODIPY conjugates titrated against HSA: Binding affinity of **18d**, **20d** and BODIPY-OH was extrapolated to be 6 μM, >82 μM and >320 μM binding affinity. (B) FP assay for BODIPY conjugates titrated against fatty acid-free HSA: Binding affinity of **18d**, **20d** and BODIPY-OH was extrapolated to be 4 μM, >112 μM, and >196 μM. (C) FP assay for BODIPY conjugates titrated against whole mouse serum: Binding affinity of **18d**, **20d** and BODIPY-OH was extrapolated to be 4 μM, >100 μM, and >80 μM.

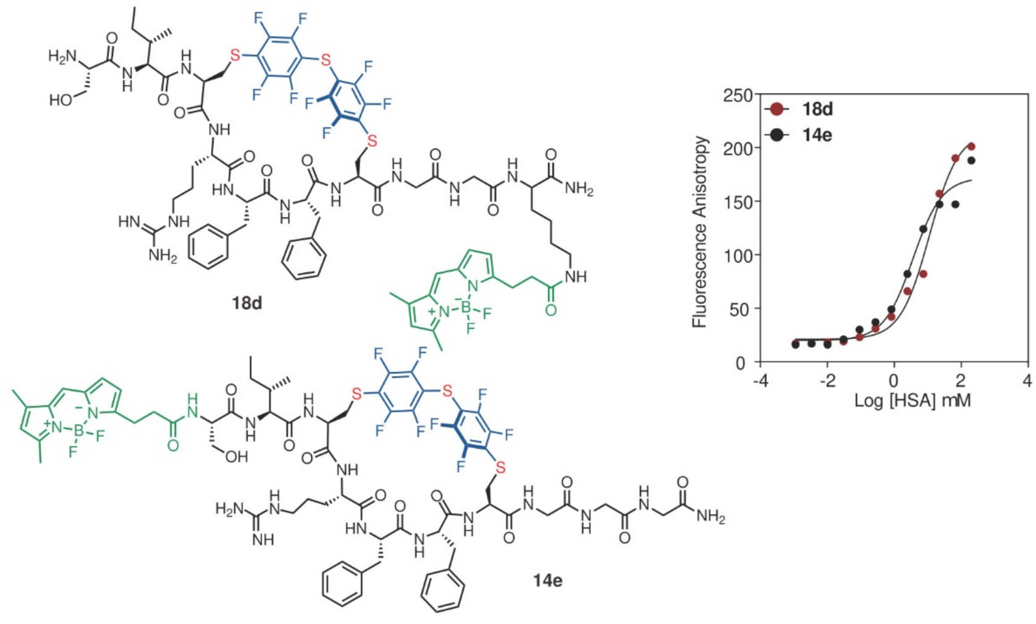

Supplementary Fig. 17. Binding of **PFS**-SICRFFC with C- or N-term BODIPY to HSA. Peptides with BODIPY conjugated to C-terminus (**18d**) or N- terminus (**14e**) were titrated against HSA. FP assay extrapolated a similar affinity for **18d** and **14e**.

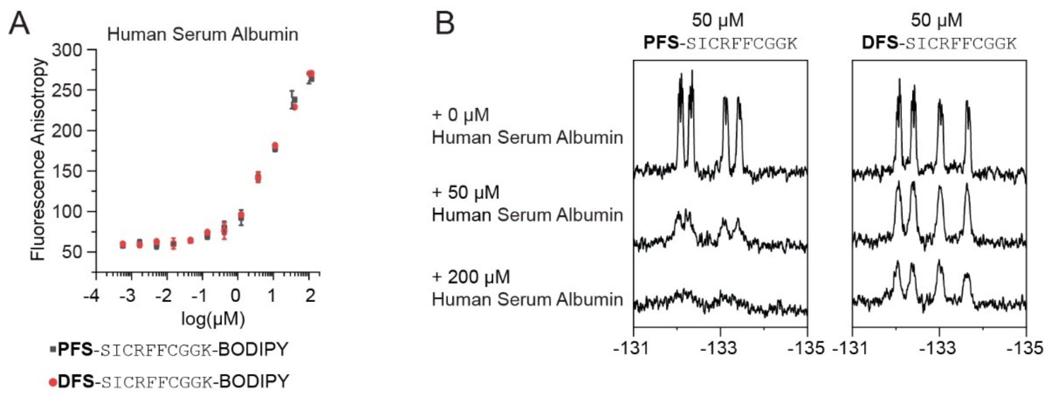

Supplementary Fig. 18. Comparison of **DFS** and **PFS**-modified peptides by FP and NMR (A) FP binding assay of **18d** and **18f** (B) ^{19}F NMR binding assay of **18b** and **18c**.

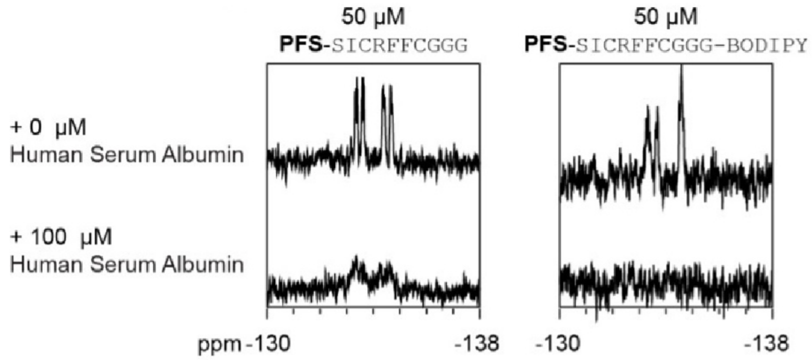

Supplementary Fig. 19. ^{19}F NMR comparison of BODIPY labeled and unlabeled peptides **PFS-**SICRFFCGGG (**14c**) and BODIPY labeled **PFS**-SICRFFCGGG (**14e**).

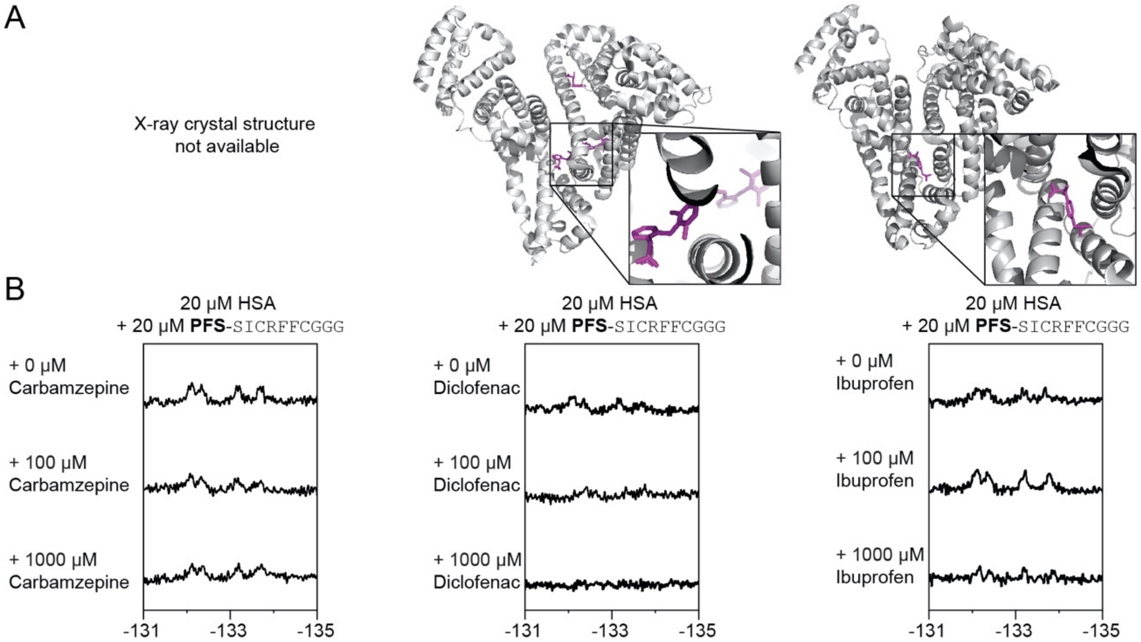

Supplementary Fig. 20. Competition of **14c** and albumin binders measured by ^{19}F NMR. (A) X-ray crystal structure of diclofenac bound HSA (PDB ID: 4Z69 [https://www.rcsb.org/structure/4Z69]) and ibuprofen bound HSA (PDB ID: 2BXG [https://www.rcsb.org/structure/2BXG]). (B) ^{19}F NMR competitive inhibition assay with **14c** against carbamazepine, diclofenac and ibuprofen.

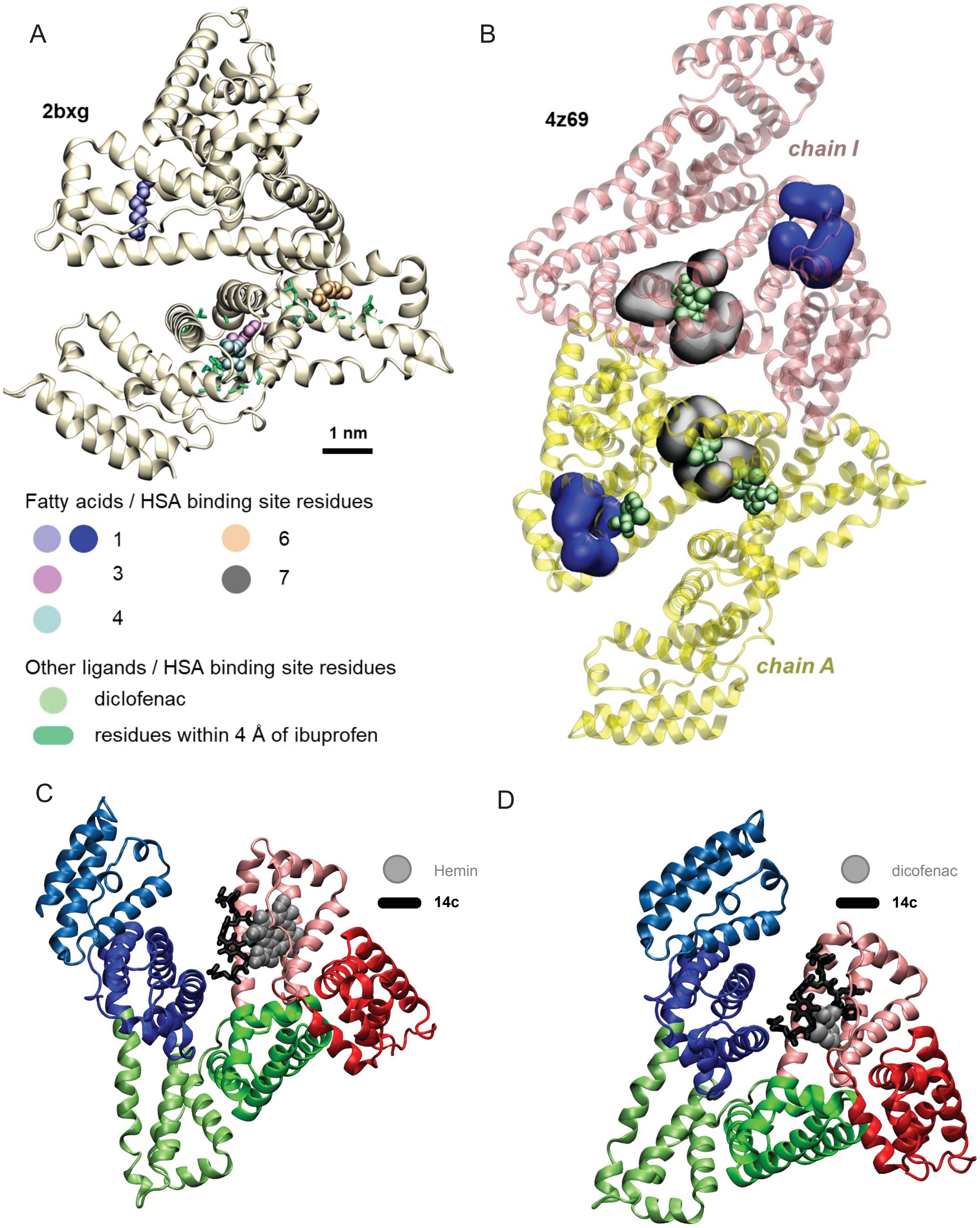

Supplementary Fig. 21. HSA binding sites of known ligands. (A) HSA binding site residues, including fatty acid binding sites (PDB ID: 2bxg [https://www.rcsb.org/structure/2BXG]). (B) Structure of two HSA chains and four diclofenac molecules in (PDB ID: 4z69 [https://www.rcsb.org/structure/4Z69]). (C) Superposition of **14c**-HSA (docking) and hemin-HSA (PDB 1O9X [https://www.rcsb.org/structure/1OX9]). (D) Superposition of **14c**-HSA complex (docking) with diclofenac (pdbID: 4z69 [https://www.rcsb.org/structure/4Z69]) in IB site. **14c** is shown in black licorice, and diclofenac is shown with gray van der Waals spheres.

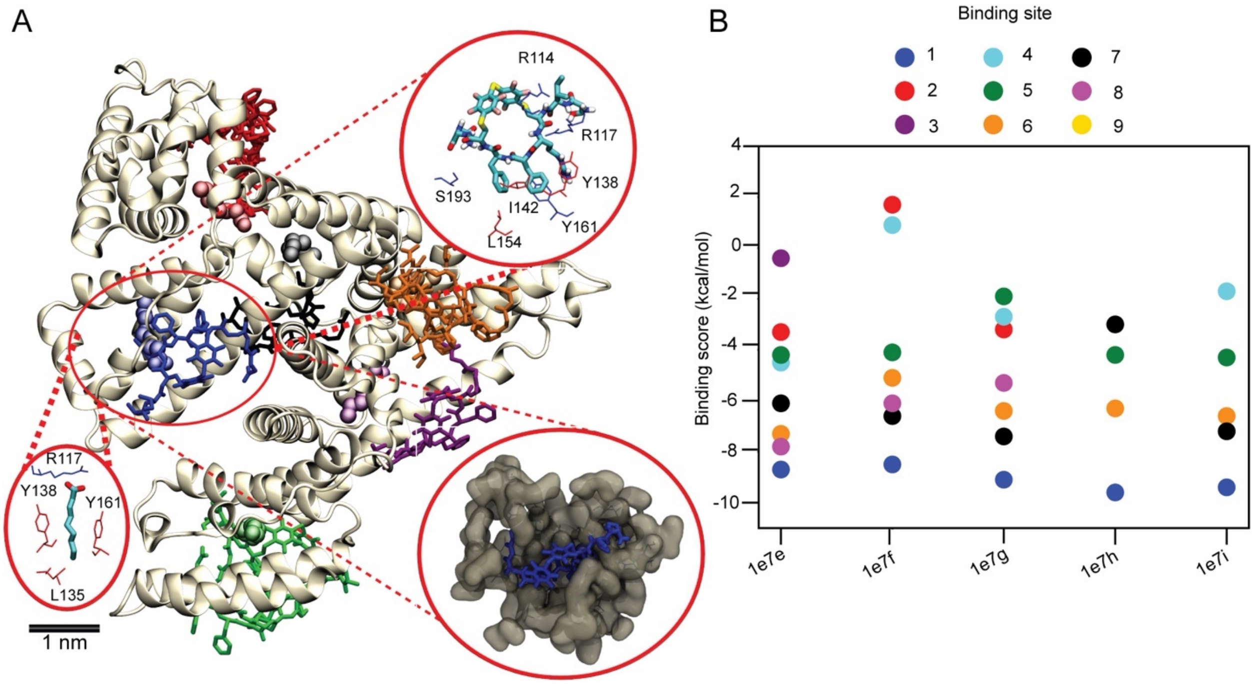

Supplementary Fig. 22. Docking calculations for **PFS**-SICRFFCGGG (**14c**) and HSA. (A) HSA protein structure with bound fatty acids overlaid with **14c** docked to the respective fatty acid binding sites on the HSA surface. Binding site 1 is circled in red. Thin licorice: amino acids directly connected to the fatty acid. Amino acids shown in blue form hydrogen bonds with the fatty acid, and amino acids shown in red form van der Waals (nonpolar) interactions with the fatty acid. (B) Plots of **14c**–HSA binding scores obtained in one set of docking calculations with structures from PDB IDs: 1e7e https://www.rcsb.org/structure/1E7E], 1e7f [https://www.rcsb.org/structure/1E7F], 1e7g [https://www.rcsb.org/structure/1E7G], 1e7h [https://www.rcsb.org/structure/1E7H], and 1e7i [https://www.rcsb.org/structure/1E7I].

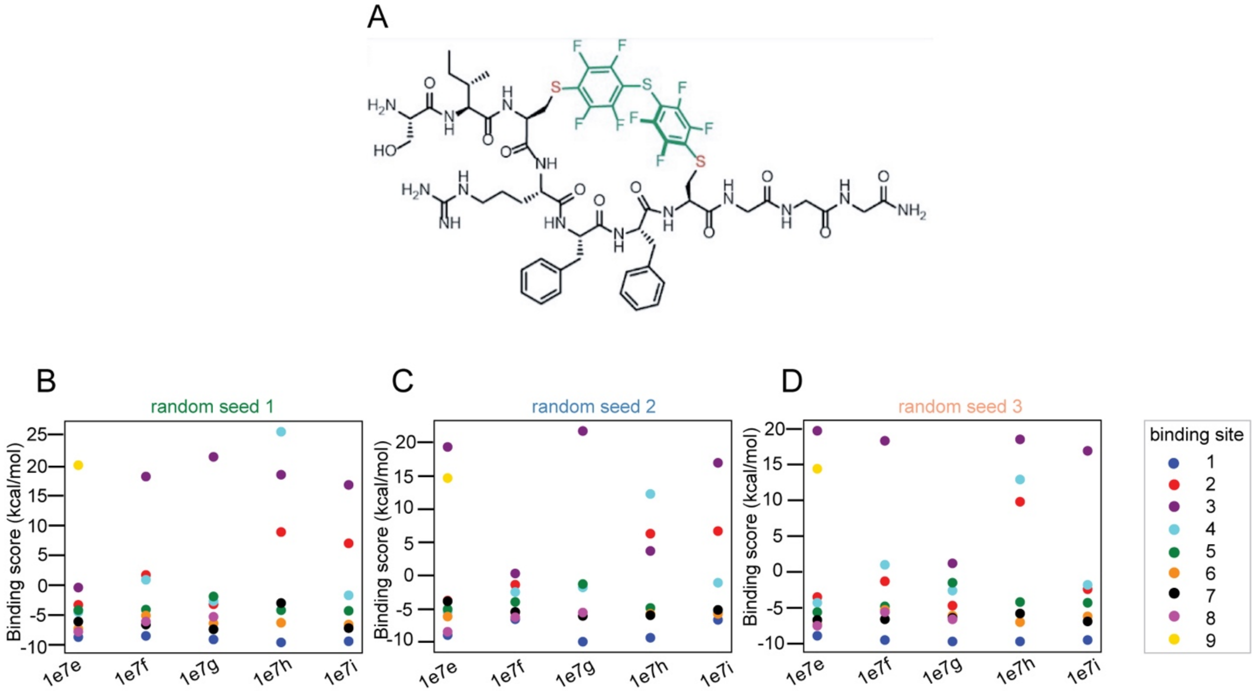

Supplementary Fig. 23. Docking scores of **14c** and HSA binding sites of fatty acids. (A) **PFS**-SICRFFCGGG macrocycle (**14c**). (B-D) Plots of **14c**-HSA binding scores from docking calculations using three different random seed values. Separate docking calculations were performed for different HSA structures extracted from PDB Databank files with PDB IDs 1e7e [https://www.rcsb.org/structure/1E7E], 1e7f [https://www.rcsb.org/structure/1E7F], 1e7g [https://www.rcsb.org/structure/1E7G], 1e7h [https://www.rcsb.org/structure/1E7H], and 1e7i [https://www.rcsb.org/structure/1E7I]. **PFS**-SICRFFCGGG macrocycles were docked to previously reported six to nine fatty acid binding sites on HSA surface[6], each labeled by a different color. Some of the HSA fatty acid binding sites and the resulting **14c**-HSA binding modes are shown in Supplementary Fig. 21.

Supplementary Table 6: Binding scores of **14c** to HSA all fatty acid binding sites

	binding scores (kcal/mol)																
binding sites	random seed 1					random seed 2					random seed 3					average	standard deviation
	1e7e	1e7f	1e7g	1e7h	1e7i	1e7e	1e7f	1e7g	1e7h	1e7i	1e7e	1e7f	1e7g	1e7h	1e7i		
1	-8.7	-8.5	-9.1	-9.6	-9.4	-9	-6.6	-10	-9.4	-6.7	-8.9	-9.5	-9.7	-9.7	-9.5	-8.95	1.02
2	-3.3	1.7	-3.2	8.9	7	-3.8	-1.4	-5.6	6.3	6.7	-3.5	-1.3	-4.7	9.8	-2.4	0.75	5.44
3	-0.4	18.2	21.5	18.5	16.8	19.4	0.3	21.8	3.7	17	19.7	18.3	1.2	18.5	16.9	14.09	8.22
4	-4.5	0.9	-2.7	25.7	-1.7	-5.5	-2.5	-1.8	12.3	-1.1	-4.3	1	-2.6	12.9	-1.8	1.62	8.62
5	-4.2	-4.1	-1.9	-4.2	-4.3	-5.1	-4	-1.3	-4.9	-5.7	-5.6	-4.8	-1.5	-4.2	-4.3	-4.01	1.37
6	-7.3	-5.1	-6.4	-6.3	-6.6	-6.2	-5.6	-5.6	-5.8	-5.8	-7.3	-5.3	-6.1	-7	-6.2	-6.17	0.67
7	-6.1	-6.6	-7.4	-3	-7.2	-3.9	-5.5	-6.1	-6	-5.2	-6.7	-6.6	-6.4	-5.8	-6.9	-5.96	1.19
8	-7.8	-6.1	-5.3	-	-	-8.5	-6.3	-5.6	-	-	-7.5	-5.6	-6.6	-	-	-6.59	1.11
9	20.1	-	-	-	-	14.7	-	-	-	-	14.4	-	-	-	-	16.40	3.21

Supplementary Table 7: Ligands that overlap with **14c** at the FA1 IB binding site.

Ligand type	Ligand name	PDB ID (ligand-HSA)	Distance between **14c** and ligand COMs (Å)	Panel in Supplementary Figure **23**
Oncology drug	(2S)-2-[1-amino-8-(hydroxymethyl)-9-oxo-9,11-dihydroinnolizino[1,2-b]quinoline-7-yl]-2-hydroxybutanoic acid	4L8U	3.85	A
Chemotherapeutic agents in HIV	3'-azido-3'-deoxythymidine	3B9L	3.89	B
Anti-inflammatory drug	Azapropazone		4.24	C
Anti-inflammatory drug	indomethacin	2BXM<br>2BXI	3.19	D

The overlap is evaluated by calculating the center-of mass (COM) distances between ligands and **14c** molecules.

Supplementary Table 8: Amino acids that contact **14c** and Hemin in HSA IB (1O9X).

AA No.	**14c**	HEMIN(1O9X)
1	ala191	ala158
2	ala194	arg114
3	arg114	his146
4	arg117	ile142
5	arg186	leu139
6	asp183	leu154
7	asp187	lys190
8	gly189	met123
9	his146	phe149
10	ile142	phe157
11	leu115	tyr138
12	leu154	tyr161
13	leu182	val116
14	lys190	
15	met123	
16	ser193	
17	tyr138	
18	tyr161	
19	val116	
total of overlap		9
Distance between COMs		6.8 Å

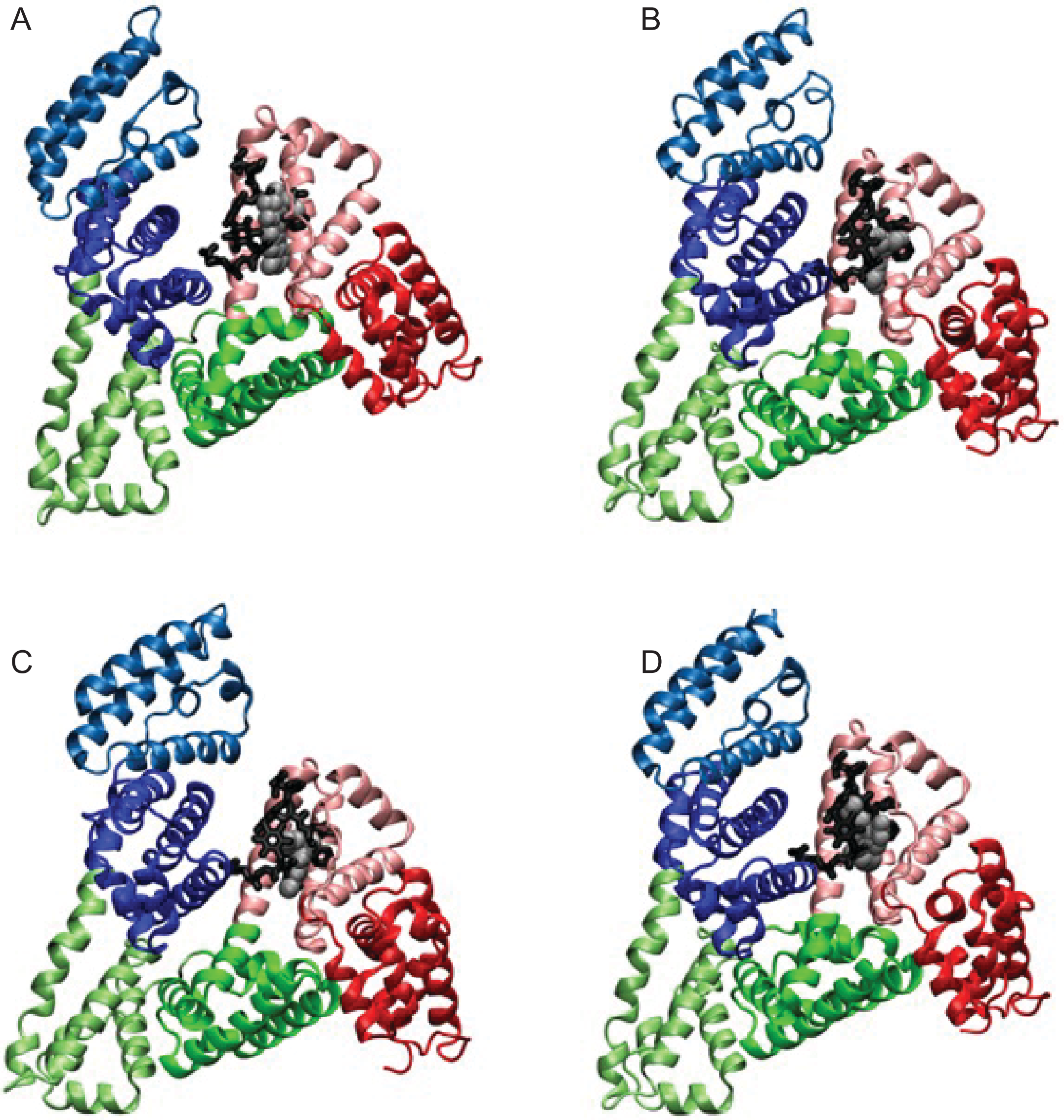

Supplementary Fig. 24. Superposition of calculated **14c**-HSA complex and crystal structures of HSA binding to four different ligands in FA1 IB site. **14c** is shown in black licorice, and ligands are shown as gray van der Waals spheres. The docking score of **14c** to FA1 IB site within HSA is -10 kcal/mol. The ligand positions were obtained from crystal structures with the following PDB IDs: (A) 4L8U [https://www.rcsb.org/structure/4L8U], (B) 3B9L [https://www.rcsb.org/structure/3B9L], (C) 2BXI [https://www.rcsb.org/structure/2BXI], (D) 2BXM [https://www.rcsb.org/structure/2BXM].

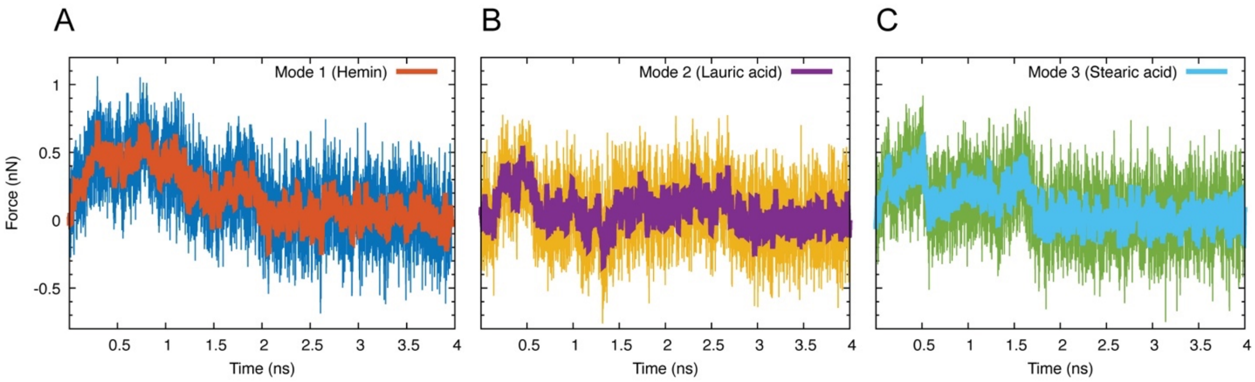

Supplementary Fig. 25. The pulling force profiles of SMD simulations.
Steered molecular dynamics (SMD) simulations of binding mode 1 (A) that binds to hemin binding site, binding mode 2 (B) to lauric acid and binding mode 3 (C) to stearic acid. Thick lines indicate running averages over 50 ps. Mode 1 shows a slightly larger maximum pulling force than the others in 0.5-1 ns regime, which means some interactions may persist in that region, while they are broken in the others.

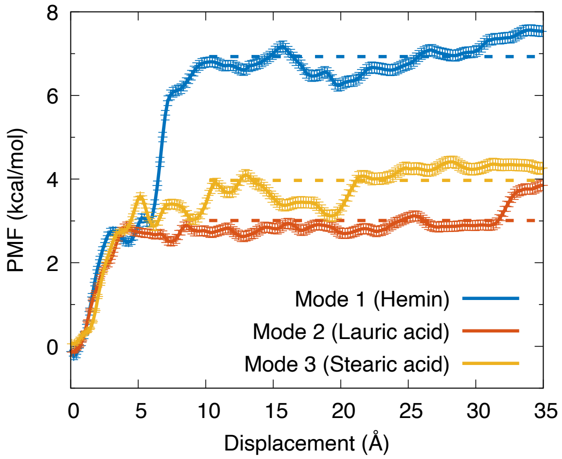

Supplementary Fig. 26. PMFs calculation of 14c in different binding sites.
PFM of mode 1, 2 and 3 c Potential of mean forces (PFM) were calculated by umbrella sampling and weighted histogram method. The plots were shifted to align their origins. Dashed lines are average PMF over the distance of 10Å and farther, where it is considered that the peptide is completely detached from the albumin. Therefore, the inverses of the heights from the origin to the dashed lines read the estimated binding affinity of each binding mode and the measured binding affinities are -7.0, -3.0 and -4.0 kcal/mol for mode 1, 2 and 3, respectively.

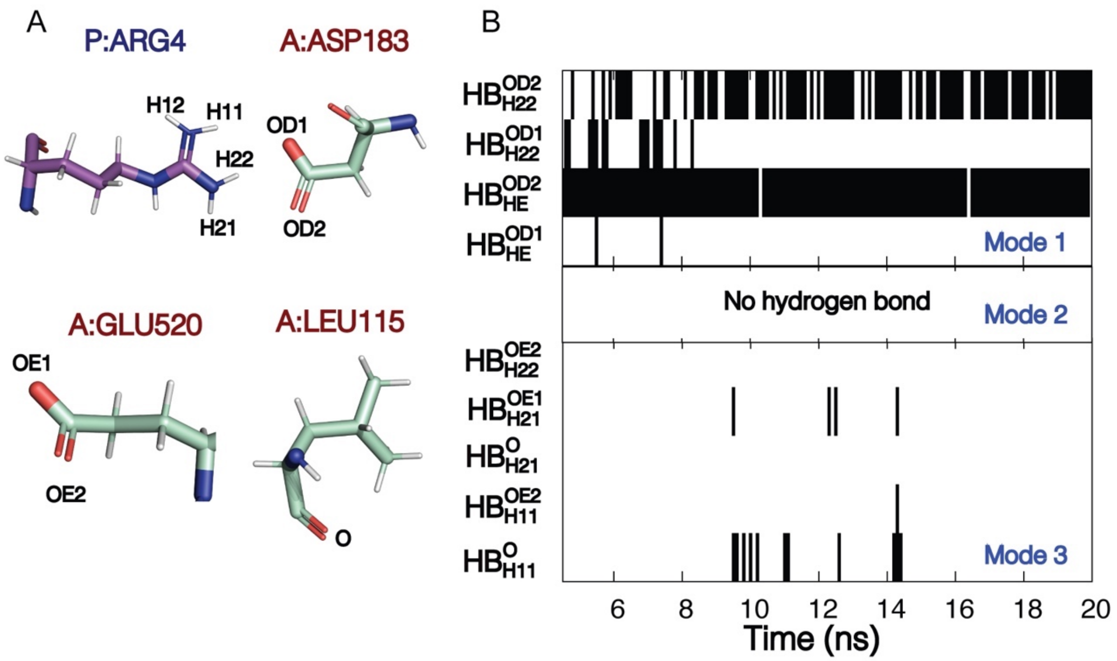

Supplementary Fig. 27. Hydrogen bond analyses for **14c** in different binding sites
Hydrogen Bond Analyses of 9Å distance umbrella sampling trajectories with the 3.5 Å cut off between a donor and an acceptor with respect to ARG4 of the macrocyclic peptide for mode 1, 2 and 3. (A) Illustration of atom labeling in the analyses. P:ARG4 is from the peptide and all the other three are from the albumin. We used the same labeling as in (B). (B) Heatmap of the hydrogen bonds between P:ARG4 and albumin for mode 1, 2 and 3. Here HB_D^A indicates a hydrogen bond between a donor D and an acceptor A. The heatmap of mode 1demonstrates that P:ARG4 participates in hydrogen bonds during almost entire simulation (>90%), by contrast, mode 2 and 3 show no or very small numbers (<10%).

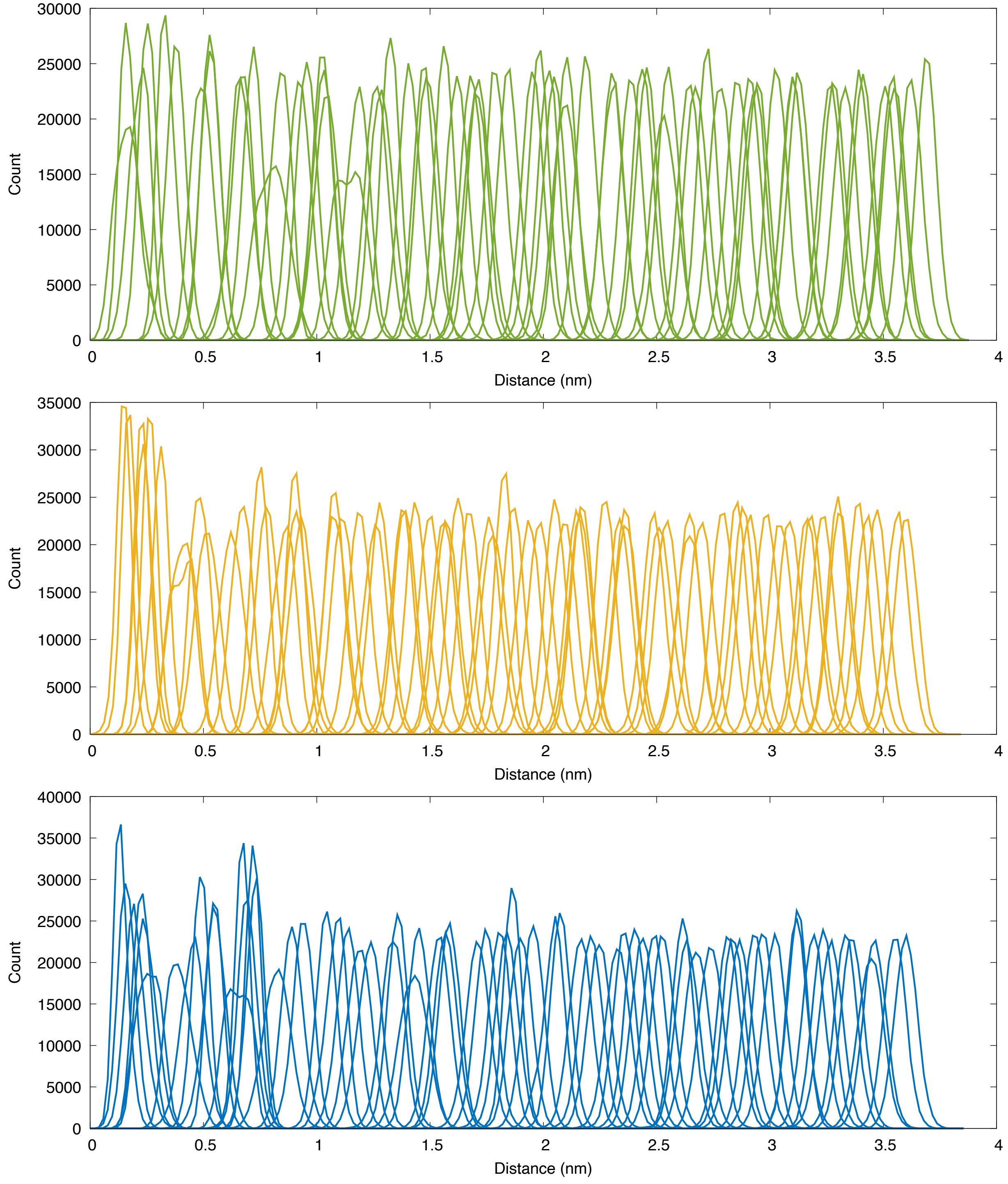

Supplementary Fig. 29. Analysis of distances between **14c** and different binding pockets Histograms of distances between the center of mass of macrocyclic peptide and binding pockets with different constraint forces for mode 1 (top), 2 (middle), 3 (bottom). In all plots, the histograms cover all distance ranges without gap, which indicates that our force constraints and distance windows are good enough to obtain converged results without biases due to the paucity of the data in certain regions

Supplementary Table 9: λ-schedule used in FEP calculations.

#	λ_{elec}	λ_{vdw}	#	λ_{elec}	λ_{vdw}
1	0	0	15	1	0.10
2	0.05	0	16	1	0.20
3	0.15	0	17	1	0.30
4	0.20	0	18	1	0.40
5	0.25	0	19	1	0.50
6	0.30	0	20	1	0.60
7	0.40	0	21	1	0.65
8	0.50	0	22	1	0.70
9	0.60	0	23	1	0.75
10	0.70	0	24	1	0.80
11	0.80	0	25	1	0.85
12	0.90	0	26	1	0.90
13	1	0	27	1	0.95
14	1	0.05	28	1	1

λ_{elec} and λ_{vdw} are λ values for electrostatic potential and van der Waals interactions, respectively.

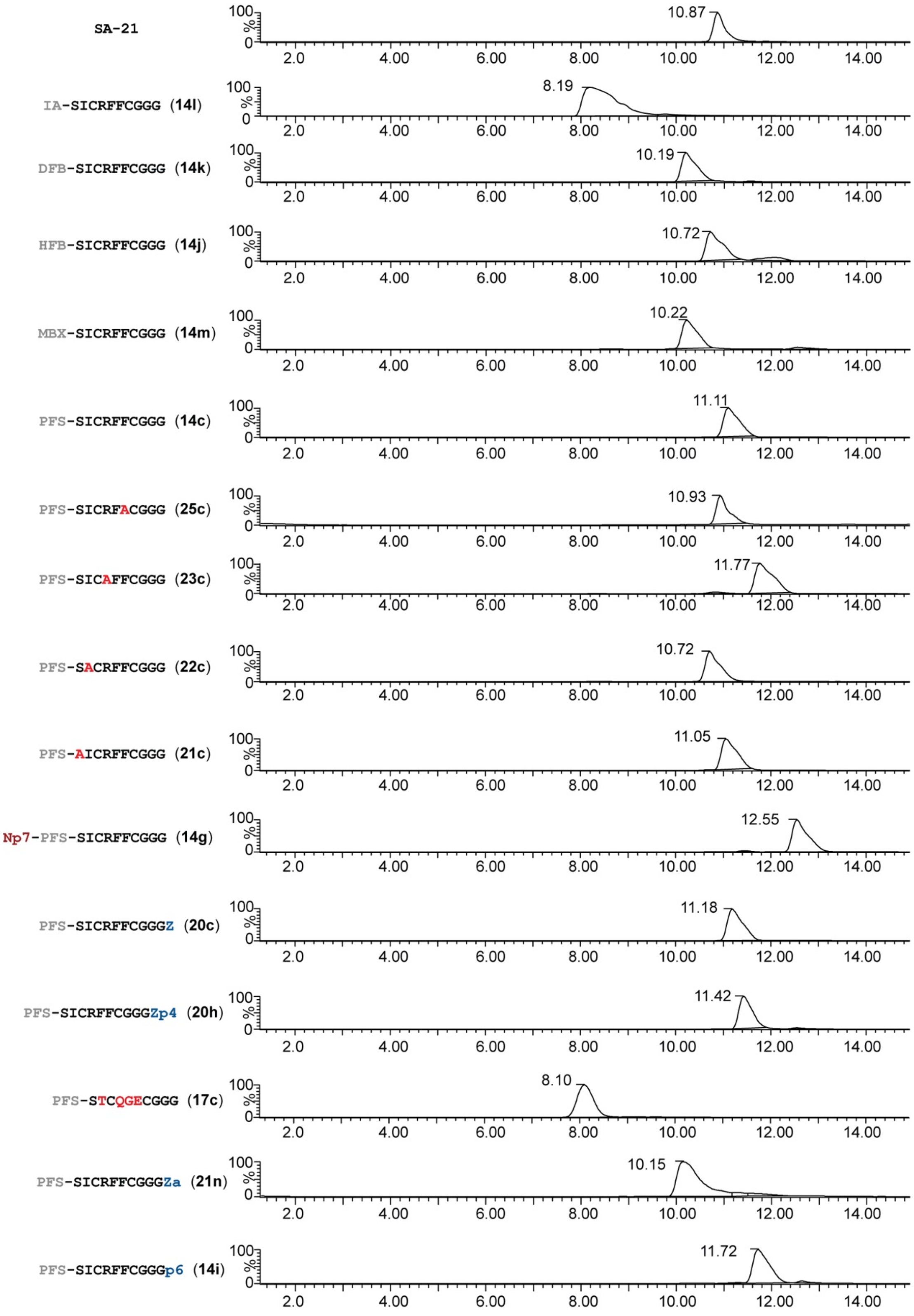

Supplementary Fig. 30. Detection of macrocycles using mass spectrometry. Selected ion chromatograms of macrocycles tested in the pharmacokinetic studies.

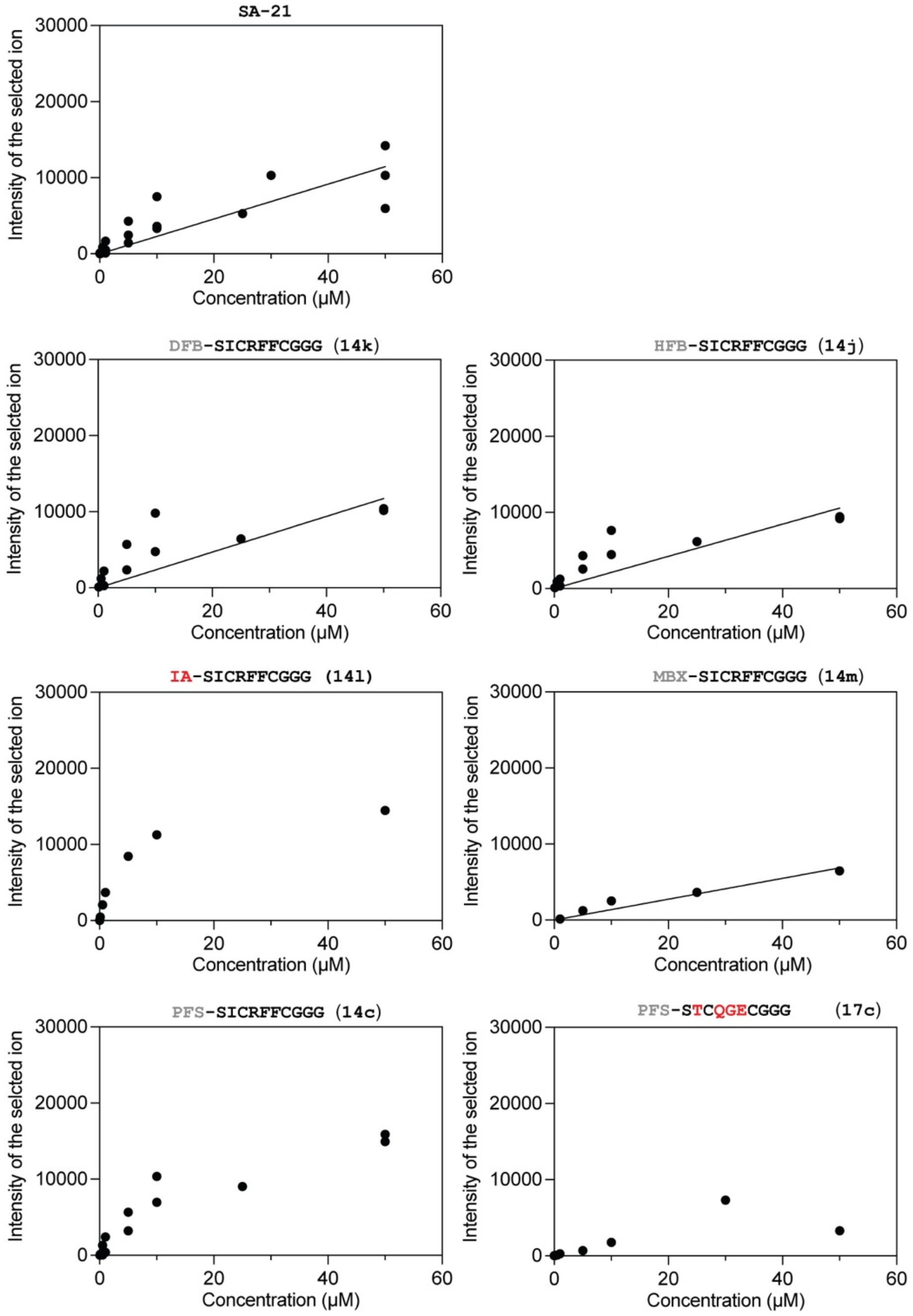

Supplementary Fig. 31. Standard curves for selected ions of SA-21, **14k-j-m-l-c**, and **17c**

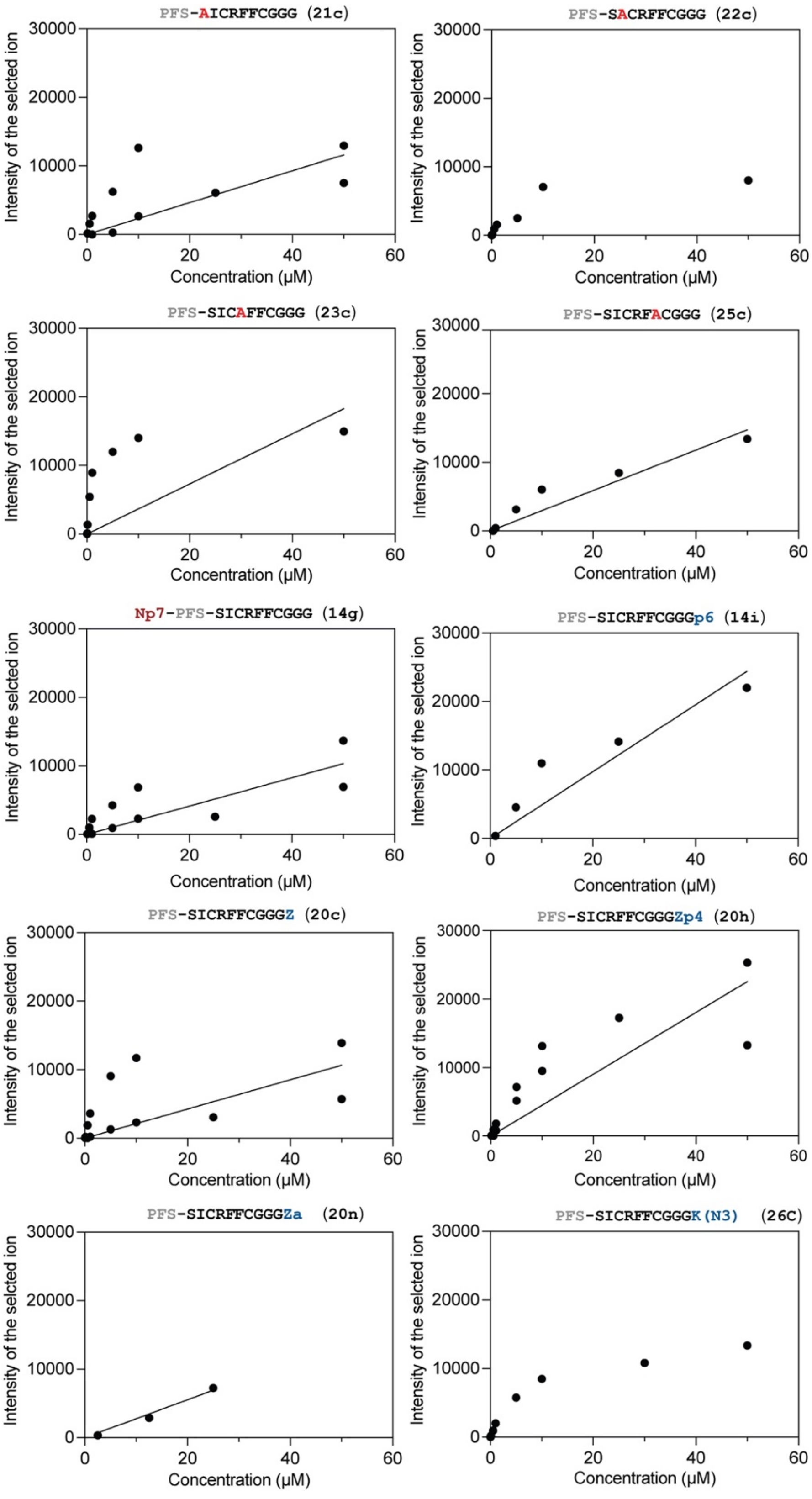

Supplementary Fig. 32. Standard curves for selected ions of **21-25c**, **14g-i**, **20c-h-n**, **26c**

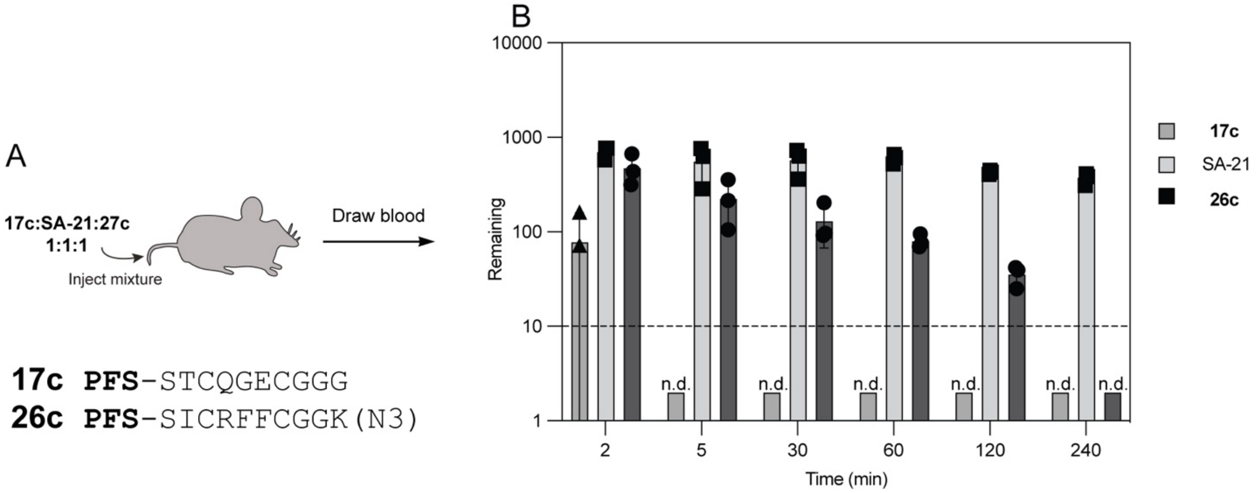

Supplementary Fig. 33. Pharmacokinetic studies for **26c**, **17c** and SA-21. (A) An equimolar (0.1 mM) mixture of macrocycles was injected into mice and blood was drawn at various time points. (B) Blood samples were collected at time points 2, 5, 30, 60, 120, and 240 mins and analyzed by LC-MS (*n* = 3, these are biological replicates). The dotted horizontal line represents the limit of detection. (n.d.: not detected).

Supplementary Table 10: Summary of in vivo experiments

Experiment reported in Figure	Blood withdrawn at time points	sex and age
Figure 6B	2 min, 60 min	3 male mice (6-12 weeks)
Figure 6C-E	2 min, 60 min, 180 min	4 female mice (6-12 weeks)
Supplementary Figure 32	2 min, 5 min, 30 min, 60 min, 120 min, 240 min	3 male mice (6-12 weeks)

31. MATLAB script for DE analysis

```
clear;

Dir='';
File = 'YW_unfiltered_20170829_ed.txt';


SET[1]  = 1:3;       % HSA
SET[2] = 4:9;          % T4-GP1
SET[3]  = 10:12;     % ConA
SET[4]  = 13:15;     % Input

TEST_SET = 1;
TARGET = 'HSA';
CONTROL_SETS = [2 3 4];

REREAD=1;   % change to zero if you don’t want to wait for -re-reading of data

close all

OUTPUT='normalized';   % other output_type: 'normalized' 'normalized+1' 'raw'

if REREAD
    disp('reading...');
    [Nuc, AA, Fr] = readMulticolumn('Dir', Dir, 'File', File, ...
                                    'column', 1:max(cell2mat(SET)),...
                                    'skip', 2, 'output', OUTPUT);
end
%%% To plot Figure S1D change the variables above to the variables below
% TEST_SET = 9;
% CONTROL_SETS = [10 11];

HITS2DISPLAY = 50; % maximum numer of hits to display
SHOWaminoACIDS = [18 19 20 21 22 23 24 25 26 27];

CLUSTERbyH = 1;          % 1 if you want your hits to be clustered by Hamming dist.
PLOT_VOLCANO = 1;        % set to 1 if you want to see the actual volcano plot
Sort_CXC_file = 1;       % set to 1 if you want to sort the reslt into different CXC files
AA_AXA_Analysis = 1;     % set to 1 if you want to see the actual AA_AXA_Analysis

%%%%%%% volcano plot parameters here %%%%%%%%%%%%%%%%%%%%%%%%%%%%%%%%%%%%%%
p_cutoff = 0.05;            % p-value cutoff
R_cutoff = 3;               % ratio cutoff
MaxX=6;                     % maximum on the X-scale (if plotting volcano)
vert_cutoff = 0.00001;      % maximum on the Y-scale (if plotting volcano)
%%%%%%%%%%%%%%%%%%%%%%%%%%%%%%%%%%%%%%%%%%%%%%%%%%%%%%%%%%%%%%%%%%%%%%%%%%%%



%%%%%%%%%%%%%%%%%%%%%% do not change things beyond this point %%%%%%%%%%%%%
%%%%%%%%%%%%%%%%%%%%%% unless you know what you are doing %%%%%%%%%%%%%%%%%

if CLUSTERbyH == 1
    disp('Culster on')
else
    disp('Culster off')
end

if PLOT_VOLCANO == 1
    disp('Volcano Plot on')
else
    disp('Volcano plot off')
end

if Sort_CXC_file == 1
    disp('Sort CXC on')
else
    disp('Sort CXC off')
end
```

```
if AA_AXA_Analysis == 1
    disp('AA AXA Analysis on')
else
    disp('AA AXA Analysis off')
end

SAVEto = [File(1:end-4) TARGET '_'...
                         OUTPUT...
                         'CONTR_' num2str(CONTROL_SETS) '_'...
                         '_P' num2str(p_cutoff)...
                         '_R' num2str(R_cutoff)...
                         '.CSV'];  % keep blank if don't want to save


% select only the aminoacids you want to see
cAA = char(AA);
sAA=cellstr(cAA(:,SHOWaminoACIDS));
SQUARE=zeros(size(Fr,1),1);


i=0;
disp('calculating p and R...');
IX=zeros(size(Fr,1),numel(CONTROL_SETS));


disp('calculating p and R...');
i=0;
for j=CONTROL_SETS
    i=i+1;
    ratio(:,i) = mean(Fr(:,SET{TEST_SET}), 2) ./ mean(Fr(:,SET{j}), 2);


    [~,confi(:,i)] = ttest2(Fr(:,SET{TEST_SET})',Fr(:,SET{j})',....
                             p_cutoff,'both','unequal');

    IX(:,i) = (confi(:,i) <= p_cutoff) & (ratio(:,i) >= R_cutoff);

    SQUARE = SQUARE + ratio(:,i).^2;


    if PLOT_VOLCANO
        subplot(1,numel(CONTROL_SETS),i);

        plot(log2 (ratio(:,i)),...
            -log10(confi(:,i)),'d',...
            'MarkerSize',4,...
            'MarkerFaceColor',0.5*[1 1 1],...
            'MarkerEdgeColor',0.5*[1 1 1]); hold on;

        plot( log2 (ratio(find(IX(:,i)),i)),...
             -log10(confi(find(IX(:,i)),i)),'d',...
                    'MarkerSize',4,...
                    'MarkerFaceColor','r',...
                    'MarkerEdgeColor','r'); hold on;

        line([log2(R_cutoff) MaxX],[-log10(p_cutoff) -log10(p_cutoff)]);
        line([log2(R_cutoff)   log2(R_cutoff)],...
             [-log10(p_cutoff) -log10(vert_cutoff)]);

        xlim([-MaxX MaxX]);
    end
end

    R2 = sqrt(SQUARE);

    IXall = find( (sum(IX,2)==size(IX,2)) ); % hits that satisfy all criteria


   % you can loosen the stringency if necessary
```

```
  % IXall = find( (sum(IX,2)>=2) ); %hits that satisfy two or more criteria


    hits    = char(sAA(IXall,:));
    Rhits   = ratio(IXall,:);
    R2hits  = R2(IXall);

%%%%%%%%%%%% this is part where hits are clustered by H-dist %%%%%%%%%%%%%%

if CLUSTERbyH
    disp('clustering...');
    if numel(hits)>3
        figure(2)
        Y = pdist(hits,'hamming');
        Z = linkage(Y,'complete');
        [H,T,perm] = dendrogram(Z,0,'colorthreshold',20);
        set(H,'LineWidth',2)

        for i =1:size(hits,1)
            label{i} = i;
        end
        set(gca,'XTick', 1:1:size(hits,1), 'XTickLabel',label);

        hits    = hits(perm,:);
        Rhits   = Rhits(perm,:);
        R2hits  = R2(perm);
        IXall   = IXall(perm);
    end
end

%%%%%%%%%%%%%%%%%%%display all results as heat map%%%%%%%%%%%%%%%%%%%%%%%%%

figure(3)

if size(IXall,1)>=HITS2DISPLAY
    N=HITS2DISPLAY;  % display only the first or defined number of hits
else
    N=size(IXall,1); %display all
end

FrPPM = round(10^6*Fr);   % convert normalized fraction frequency to PPM

imagesc( log10([FrPPM(IXall(1:N),:) ratio(IXall(1:N),:) ]+1) );

set(gca,'YTick', 1:1:N, 'YTickLabel',cellstr(hits(1:N,:)),'TickDir','out',...
    'FontName','Courier New','FontSize',14);
set(gca,'XTick', 1:1:size(Fr,2)+4, 'TickDir','out');
jet1=jet;
jet1(1,:)=[0.4 0.4 0.4];
colormap(jet1);
colorbar;

% generate a plain text table for saving or copy from command line
S = char(32*ones(size(hits,1),2));
COM = char(','*ones(size(hits,1),1));

L = [ S(:,1) char(124*ones(size(hits,1),1)) S(:,1)];
if strcmp(OUTPUT,'raw')
    F  = Fr(IXall,:);   % display frequency raw
else
    F  = FrPPM(IXall,:);   % display frequency in ppm
end

toDisp = [hits     S  ];
toSave = [hits     COM  ];


for i=1:numel(SET)
    for j=1:numel(SET{i})
        toDisp = [toDisp num2str(F(:,SET{i}(j))) S];
        toSave = [toSave num2str(F(:,SET{i}(j))) COM];
```

```
    end
    toDisp = [toDisp L];
end
toDisp = [toDisp S  num2str(round(Rhits)) L];
for i=1:size(Rhits,2)
    toSave = [toSave  num2str(round(Rhits(:,i))) COM ];
end

disp(toSave);
disp(toDisp);

if ~isempty(SAVEto)

    fs = fopen(fullfile(Dir,SAVEto),'w');
    RET = char(10*ones(size(toSave,1),1));
    fprintf( fs, '%s\r\n', [toSave(:,1:end-1) RET]');
    fclose all;
    disp('file saved');
end

%%%%%%%%%%%%%%%%%%%%%%%%%%%%%%%%C3C Sorting%%%%%%%%%%%%%%%%%%%%%%%%%%%%%%%%

if Sort_CXC_file

    disp('sorting...') ;


    %Sorting conditions
    C2X = ['S' '\w' 'C' '\w' '\w' 'C' '\w' '\w' '\w'] ;
    C3X = ['S' '\w' 'C' '\w' '\w' '\w' 'C' '\w' '\w' '\w'] ;
    C4X = ['S' '\w' 'C' '\w' '\w' '\w' '\w' 'C' '\w' '\w'] ;
    C5X = ['S' '\w' 'C' '\w' '\w' '\w' '\w' '\w' 'C' '\w'] ;
    C7X = ['A' 'C' '\w' '\w' '\w' '\w' '\w' '\w' '\w' 'C'] ;
    % Creat sorting array
    C2XHits = [];
    C3XHits = [];
    C4XHits = [];
    C5XHits = [];
    C7XHits = [];
    indexs=[];              %for data purposes not really useful for now
    j=0;

    % sort C2C
    disp('Sorting C2C...')
    C2s = regexp(cellstr(hits),C2X);
    for i=1:numel(C2s)
        if ~isempty(C2s{i})
            j=j+1;
            C2XHits = [C2XHits; hits(i,:)];
            index(j) = i;
        end
    end
    %C2Cfound(s) = numel(indexs);


    %Sort C3C
    disp('Sorting C3C...')
    indexs=[];
    j=0;
    C3s = regexp(cellstr(hits),C3X);
    for i=1:numel(C3s)
        if ~isempty(C3s{i})
            j=j+1;
            C3XHits = [C3XHits; hits(i,:)];
            index(j) = i;
        end
    end
    %C3Cfound(s) = numel(indexs);

    %Sort C4C
    disp('Sorting C4C...')
```

```
    indexs=[];
    j=0;
    C4s = regexp(cellstr(hits),C4X);
    for i=1:numel(C4s)
        if ~isempty(C4s{i})
            j=j+1;
            C4XHits = [C4XHits; hits(i,:)];
            indexs(j) = i;
        end
    end
    %C4Cfound(s) = numel(indexs);

    %Sort C5C
    disp('Sorting C5C...')
    indexs=[];
    j=0;
    C5s = regexp(cellstr(hits),C5X);
    for i=1:numel(C5s)
        if ~isempty(C5s{i})
            j=j+1;
            C5XHits = [C5XHits; hits(i,:)];
            indexs(j) = i;
        end
    end
    %C5Cfound(s) = numel(indexs);

    % Sort C7C
    disp('Sorting C7C...')
    indexs=[];
    j=0;
    C7s = regexp(cellstr(hits),C7X);
    for i=1:numel(C7s)
        if ~isempty(C7s{i})
            j=j+1;
            C7XHits = [C7XHits; hits(i,:)];
            indexs(j) = i;
        end
    end
    %C7Cfound(s) = numel(indexs);

end
if ~isempty(C2XHits)
    C2 = fopen(fullfile(Dir,['C2C',SAVEto]),'w');
    RET = char(10*ones(size(C2XHits,1),1));
    fprintf(C2, '%s\r\n',[C2XHits(:,1:end-1) RET]');
    fclose all;
    disp('C2C saved');
end

if ~isempty(C3XHits)
    C3 = fopen(fullfile(Dir,['C3C',SAVEto]),'w');
    RET = char(10*ones(size(C3XHits,1),1));
    fprintf(C2, '%s\r\n',[C3XHits(:,1:end-1) RET]');
    fclose all;
    disp('C3C saved');
end

if ~isempty(C4XHits)
    C4 = fopen(fullfile(Dir,['C4C',SAVEto]),'w');
    RET = char(10*ones(size(C4XHits,1),1));
    fprintf(C2, '%s\r\n',[C4XHits(:,1:end-1) RET]');
    fclose all;
    disp('C4C saved');
end

if ~isempty(C5XHits)
    C5 = fopen(fullfile(Dir,['C5C',SAVEto]),'w');
    RET = char(10*ones(size(C5XHits,1),1));
    fprintf(C2, '%s\r\n',[C5XHits(:,1:end-1) RET]');
    fclose all;
    disp('C5C saved');
```

```
end

if ~isempty(C7XHits)
    C7 = fopen(fullfile(Dir,['C7C',SAVEto]),'w');
    RET = char(10*ones(size(C7XHits,1),1));
    fprintf(C2, '%s\r\n',[C7XHits(:,1:end-1) RET]');
    fclose all;
    disp('C7C saved');

end

%%%%%%%%%%%%%%%%AA & AxA analysis%%%%%%%%%%%%%%%%%%%%%%%%%%%%%%%%%%%%%%%%%%%

if AA_AXA_Analysis
    disp('Start AA AXA Analysis...')
    figure(100);
    AAA = 'ADEFHIKLMNPQRSTVWY';
    Y = [];

    for i=1:numel(AAA)
        Y(i) = numel(find(hits==AAA(i)));
        xlabel{i} = AAA(i);
    end

    plot(1:numel(AAA), Y, 'ok');
    set(gca, 'xTick', 1:numel(AAA), 'xTickLabel', xlabel, 'TickDir','out');


    %%
    Nfound =  [];
    NfoundS = [];
    toSaveIX = [];
    toSaveIXS = [];

    M = 9;

    fs = fopen(fullfile(Dir,['AA' SAVEto]),'w');
    fsS = fopen(fullfile(Dir,['AxA' SAVEto]),'w');
    fclose all;

    fs = fopen(fullfile(Dir,['AA' SAVEto]),'a+');
    fsS = fopen(fullfile(Dir,['AxA' SAVEto]),'a+');

    for ii = 1:numel(AAA)
        %disp(num2str(ii));
        for jj= 1:numel(AAA)


            phrase = [ AAA(ii) AAA(jj) ] ;
            phraseS = [ AAA(ii) '\w' AAA(jj) ] ;

            phraseHits = [];
            spacedHits = [];
            index=[];
            j=0;


            IX = regexp(cellstr(hits),phrase);
            for i=1:numel(IX)
                if ~isempty(IX{i})
                    %check whether his is S**** or A****; if it is, discard
                    if (phrase(1) =='S' || phrase(1) =='A')
                        if (numel(IX{i})==1 && IX{i}==1)
                            continue
                        end
                    end

                    j=j+1;
                    phraseHits = [phraseHits; hits(i,:)];
                    index(j) = i;
```

```
                    S1 = char (32*ones(1, M-IX{i}(1)));
                    S2 = char (32*ones(1, IX{i}(1)  ));
                    spacedHits = [spacedHits; S1 hits(i,:) S2];
                end
            end
            Nfound(ii,jj) = numel(index);

            % lets save this with offsets
            RET = char(10*ones(size(index,2),1));
            fprintf( fs, '%s\r\n', [spacedHits toSave(index,:) RET]');

            phraseHits = [];
            index=[];
            spacedHits = [];
            j=0;
            clear IX

            IX = regexp(cellstr(hits),phraseS);
            for i=1:numel(IX)
                if ~isempty(IX{i})
                    %check whether his is S**** or A****; if it is, discard
                    if (phraseS(1) =='S' || phraseS(1) =='A')
                        if (numel(IX{i})==1 && IX{i}==1)
                            continue
                        end
                    end

                    j=j+1;
                    phraseHits = [phraseHits; hits(i,:)];
                    index(j) = i;

                    S1 = char (32*ones(1, M-IX{i}(1)));
                    S2 = char (32*ones(1, IX{i}(1)  ));
                    spacedHits = [spacedHits; S1 hits(i,:) S2];
                end
            end
            NfoundS(ii,jj) = numel(index);
            toSaveIXS = [toSaveIXS index];

            % lets save this with offsets
            RET = char(10*ones(size(index,2),1));
            fprintf( fsS, '%s\r\n', [spacedHits toSave(index,:) RET]');

        end
    end

    figure(200);
    subplot(1,2,1);
    imagesc(Nfound); colorbar;
    set(gca, 'xTick', 1:numel(AAA), 'xTickLabel', xlabel, 'TickDir','out',...
        'yTick', 1:numel(AAA), 'yTickLabel', xlabel);

    subplot(1,2,2);
    imagesc(NfoundS); colorbar;
    set(gca, 'xTick', 1:numel(AAA), 'xTickLabel', xlabel, 'TickDir','out',...
        'yTick', 1:numel(AAA), 'yTickLabel', xlabel);


    fclose all;
    fs = fopen(fullfile(Dir,['AA' SAVEto]),'w');
    fsS = fopen(fullfile(Dir,['AxA' SAVEto]),'w');
    RET = char(10*ones(size(toSaveIX,2),1));
    fprintf( fs, '%s\r\n', [toSave(toSaveIX,:) RET]');
    disp('AA Saved')

    RET = char(10*ones(size(toSaveIXS,2),1));
    fprintf( fsS, '%s\r\n', [toSave(toSaveIXS,:) RET]');
    disp('AxA Saved')
end
```

32. Summary of synthesis

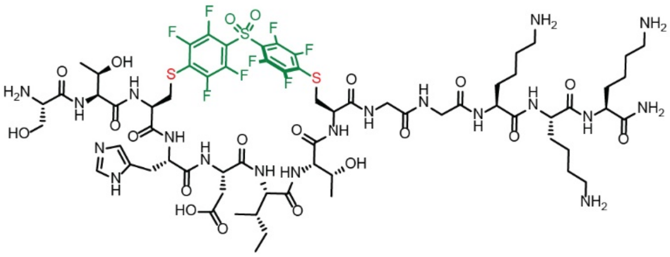

Chemical Formula: $C_{67}H_{95}F_8N_{19}O_{20}S_3$
Exact Mass: 1733.60
Molecular Weight: 1734.78

Starting material mass: 8.8 mg
Final product mass: 0.5 mg

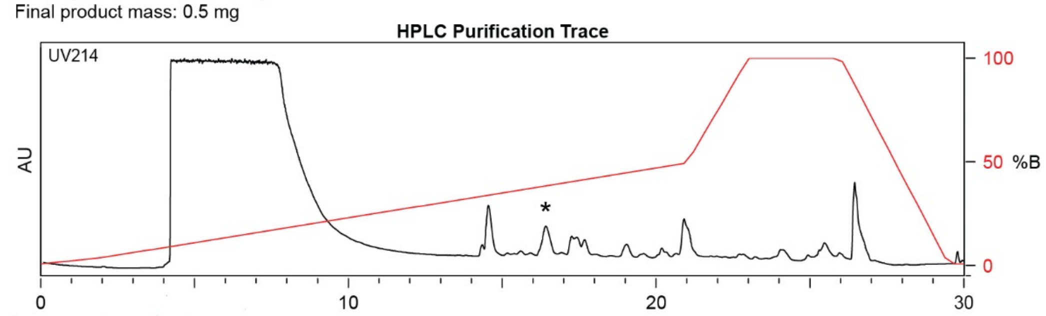

Time (min)	Solvent B (%)
0	2
5	30
24	95
25	100
27	100
28	2
30	2

Flow Rate: 13 mL / min
Phenomenex Kinetex EVO C18 Prep Column
(100 Å, 5 μm, 21.5 mm X 250 mm)

Slovent A: H_2O + 0.1% (v/v) TFA
Slovent B: MeCN + 0.1% (v/v) TFA

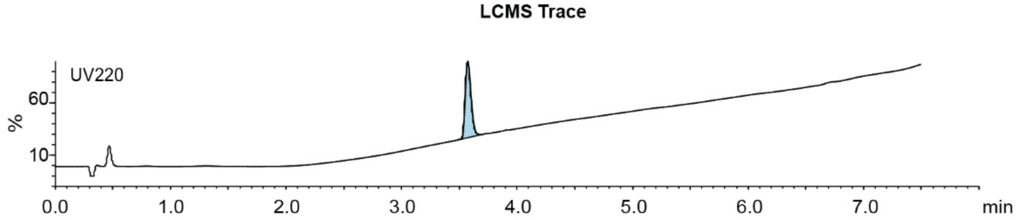

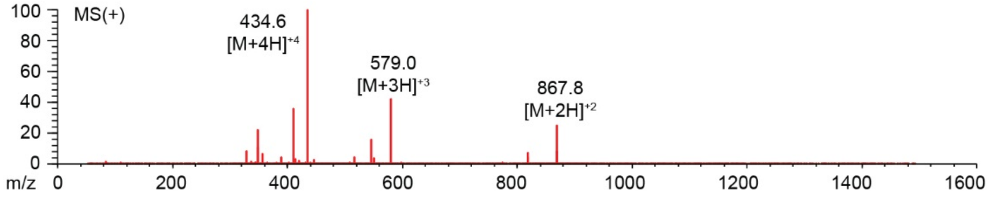

Supplementary Fig. 34. Synthesis summary of **9b DFS-**STCHDITCGGKKK

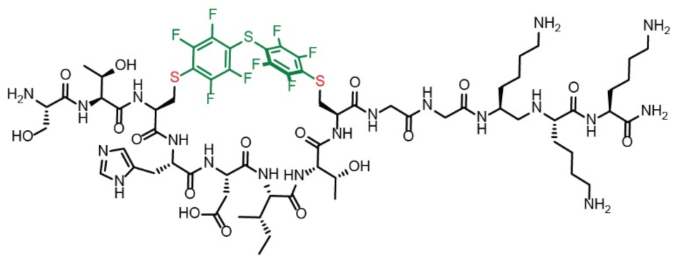

Chemical Formula: $C_{67}H_{97}F_8N_{19}O_{17}S_3$
Exact Mass: 1687.63
Molecular Weight: 1688.80

Starting material mass: 9.2 mg
Final product mass: 3.3 mg

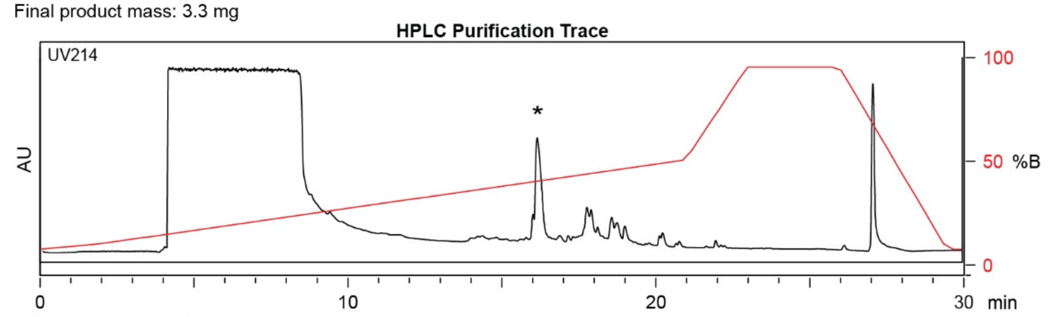

Time (min)	Solvent B (%)
0	2
2	5
21	50
23	100
26	100
29.5	2
30	2

Flow Rate: 13 mL / min
Phenomenex Kinetex EVO C18 Prep Column
(100 Å, 5 µm, 21.5 mm X 250 mm)

Solvent A: H_2O + 0.1% (v/v) TFA
Solvent B: MeCN + 0.1% (v/v) TFA

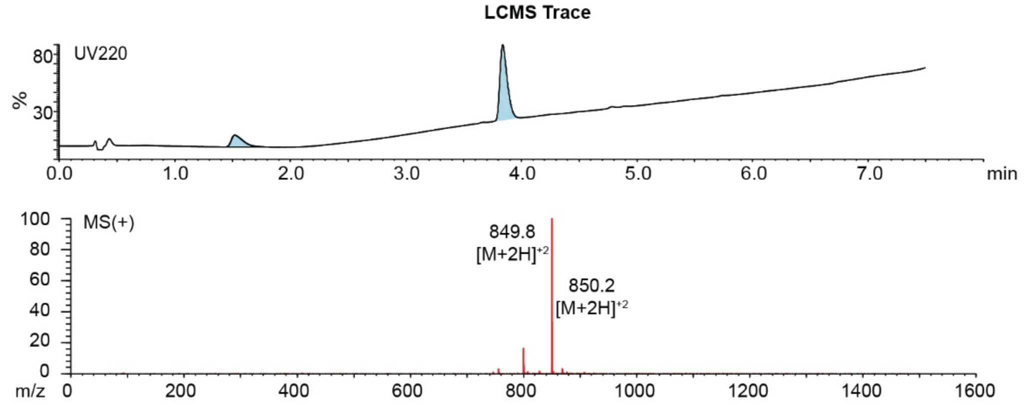

Supplementary Fig. 35. Synthesis summary of **9c PFS**-STCHDITCGGKKK

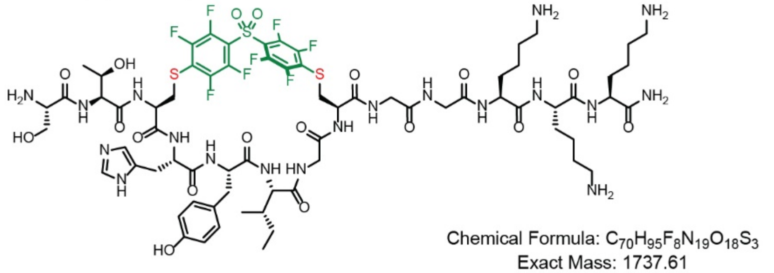

Chemical Formula: $C_{70}H_{95}F_8N_{19}O_{18}S_3$
Exact Mass: 1737.61
Molecular Weight: 1738.81

Starting material mass: 20 mg
Final product mass: 15 mg

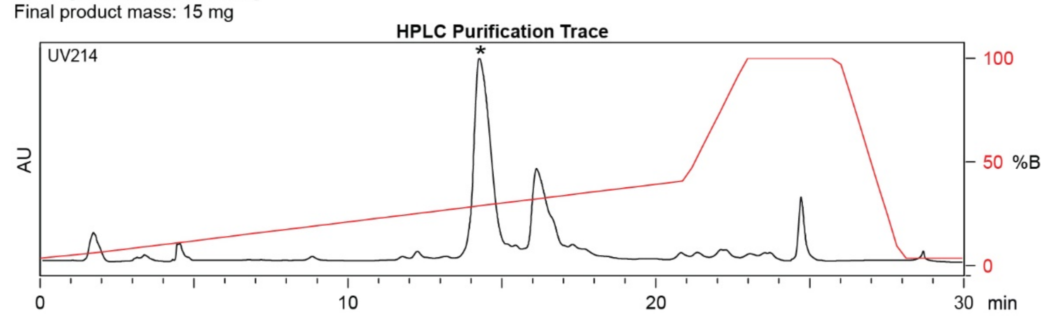

Time (min)	Solvent B (%)
0	2
2	5
21	50
23	100
26	100
29.5	2
30	2

Flow Rate: 13 mL / min
Phenomenex Kinetex EVO C18 Prep Column
(100 Å, 5 µm, 21.5 mm X 250 mm)

Solvent A: H_2O + 0.1% (v/v) TFA
Solvent B: MeCN + 0.1% (v/v) TFA

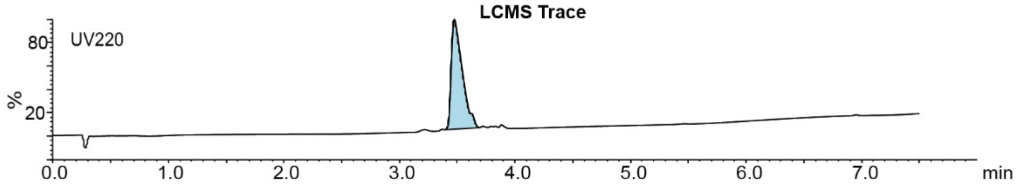

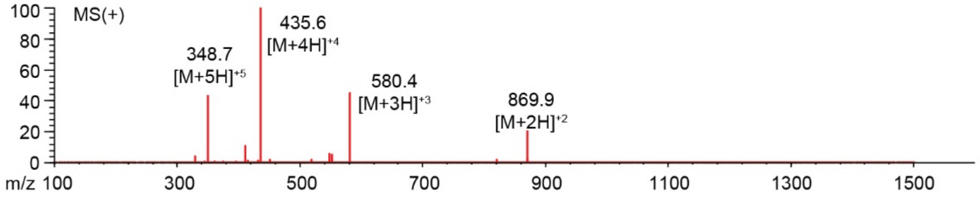

Supplementary Fig. 36. Synthesis summary of **10b DFS**-STCHYIGCGGKKK

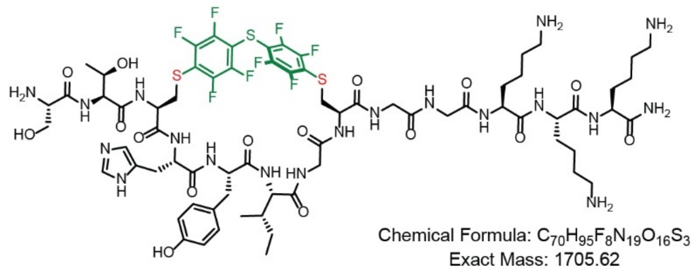

Chemical Formula: $C_{70}H_{95}F_8N_{19}O_{16}S_3$
Exact Mass: 1705.62
Molecular Weight: 1706.81

Starting material mass: 10 mg
Final product mass: 3.4 mg

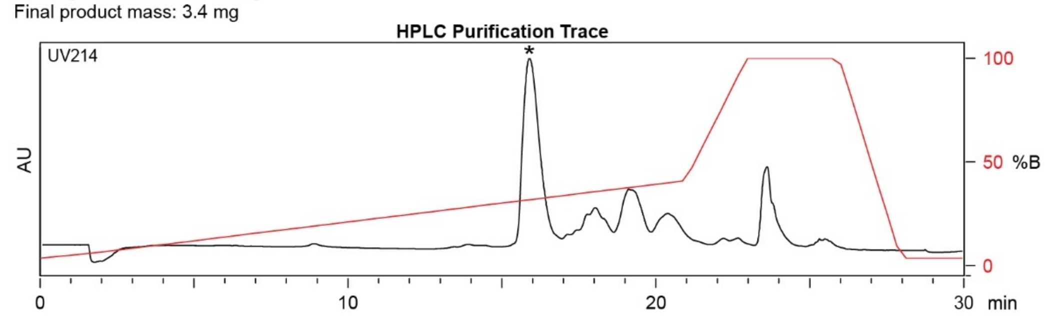

Time (min)	Solvent B (%)
0	2
2	5
21	50
23	100
26	100
29.5	2
30	2

Flow Rate: 13 mL / min
Phenomenex Kinetex EVO C18 Prep Column
(100 Å, 5 μm, 21.5 mm X 250 mm)

Solvent A: H_2O + 0.1% (v/v) TFA
Solvent B: MeCN + 0.1% (v/v) TFA

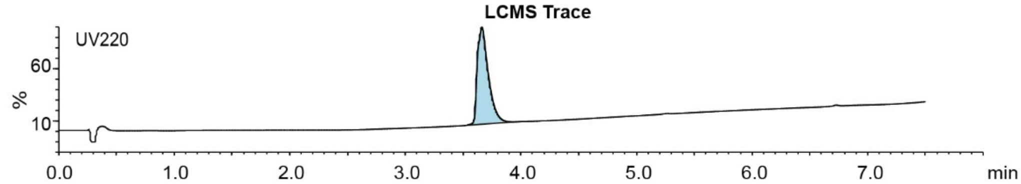

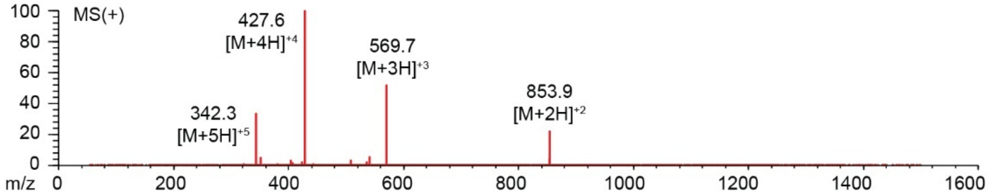

Supplementary Fig. 37. Synthesis summary of **10c PFS**-STCHYIGCGGKKK

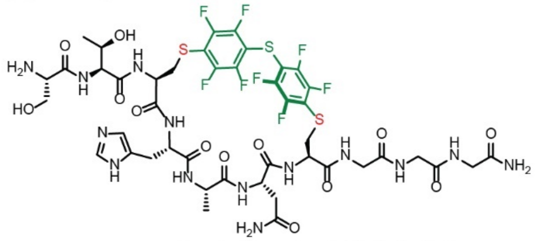

Chemical Formula: $C_{44}H_{50}F_8N_{14}O_{13}S_3$
Exact Mass: 1230.27
Molecular Weight: 1231.14

Starting material mass: 8.8mg
Final product mass: 3.3 mg

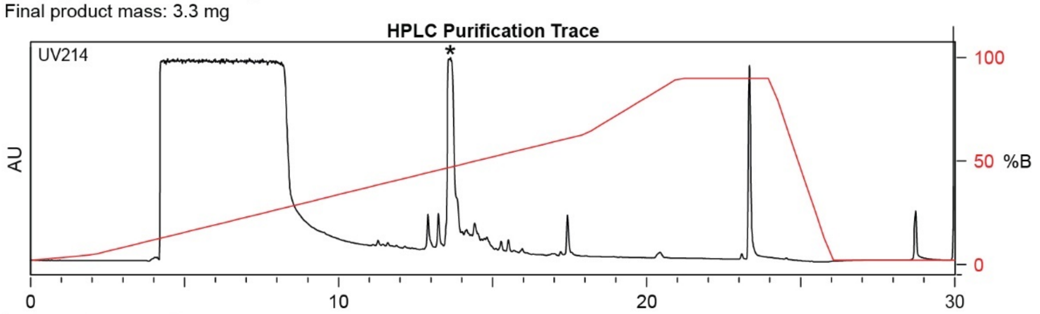

Time (min)	Solvent B (%)
0	2
2	5
18	70
21	100
24	100
26	2
30	2

Flow Rate: 13 mL / min
Phenomenex Kinetex EVO C18 Prep Column
(100 Å, 5 μm, 21.5 mm X 250 mm)

Solvent A: H_2O + 0.1% (v/v) TFA
Solvent B: MeCN + 0.1% (v/v) TFA

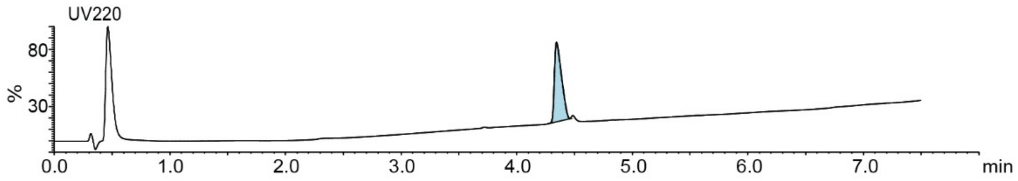

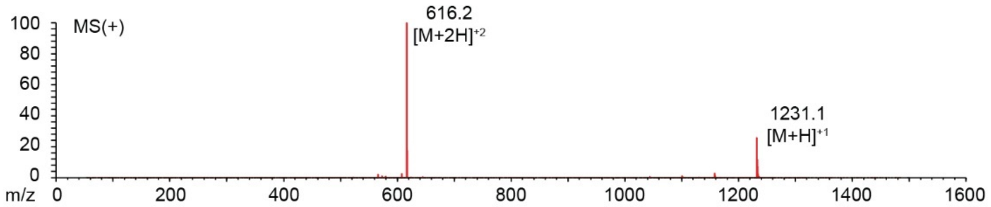

Supplementary Fig. 38. Synthesis summary of **11c PFS**-STCHANCGGG

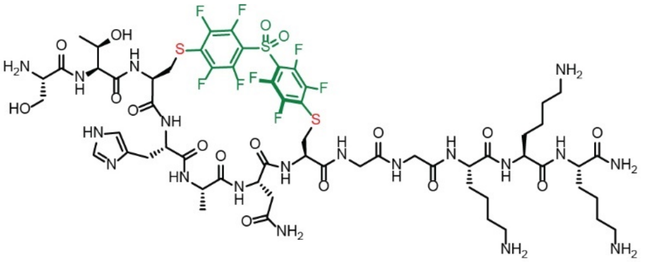

Chemical Formula: $C_{60}H_{83}F_8N_{19}O_{17}S_3$
Exact Mass: 1589.52
Molecular Weight: 1590.61

Starting material mass: 10.5 mg
Final product mass: 3.3 mg

HPLC Purification Trace

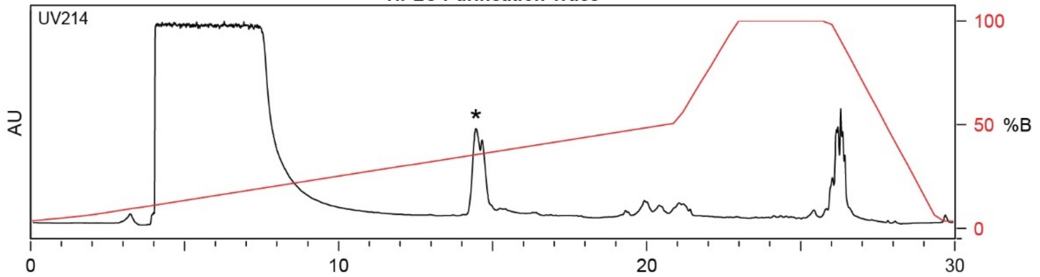

Time (min)	Solvent B (%)
0	2
2	5
21	50
23	100
26	100
29.5	2
30	2

Flow Rate: 13 mL / min
Phenomenex Kinetex EVO C18 Prep Column
(100 Å, 5 μm, 21.5 mm X 250 mm)

Slovent A: H_2O + 0.1% (v/v) TFA
Slovent B: MeCN + 0.1% (v/v) TFA

LCMS Trace

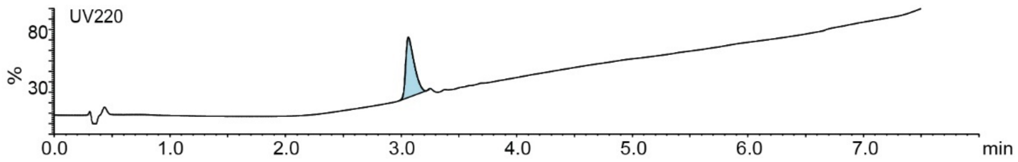

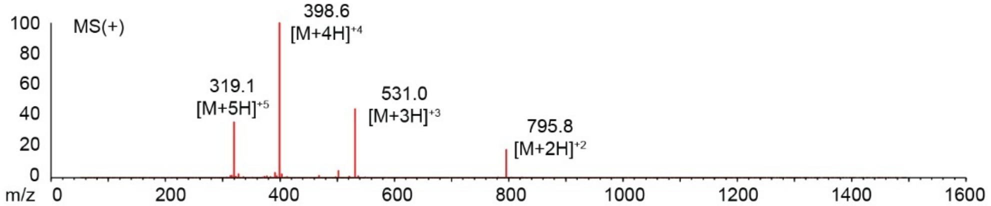

Supplementary Fig. 39. Synthesis summary of **12b DFS**-STCHANGCGGKKK

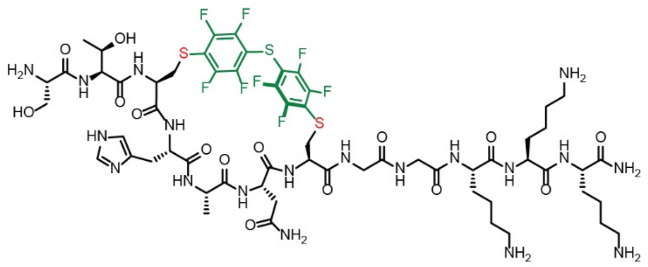

Chemical Formula: $C_{60}H_{83}F_8N_{19}O_{15}S_3$
Exact Mass: 1557.54
Molecular Weight: 1558.61

Starting material mass: 11.4 mg
Final product mass: 2.0 mg

HPLC Purification Trace

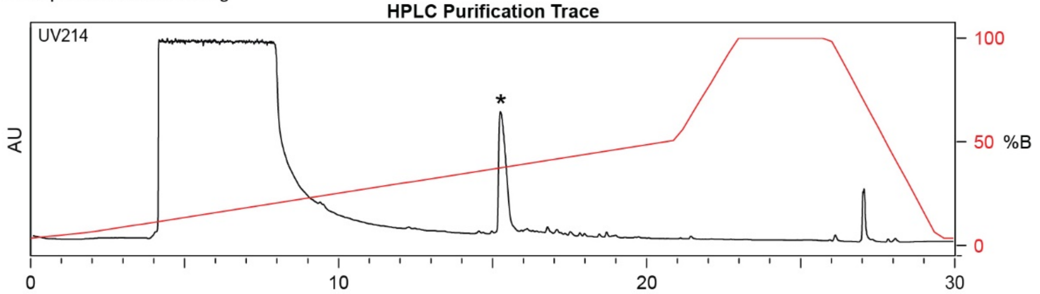

Time (min)	Solvent B (%)
0	2
2	5
21	50
23	100
26	100
29.5	2
30	2

Flow Rate: 13 mL / min
Phenomenex Kinetex EVO C18 Prep Column
(100 Å, 5 µm, 21.5 mm X 250 mm)

Solvent A: H_2O + 0.1% (v/v) TFA
Solvent B: MeCN + 0.1% (v/v) TFA

LCMS Trace

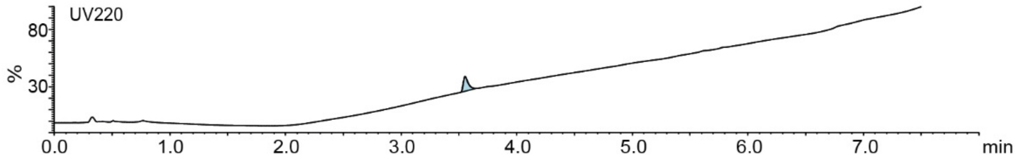

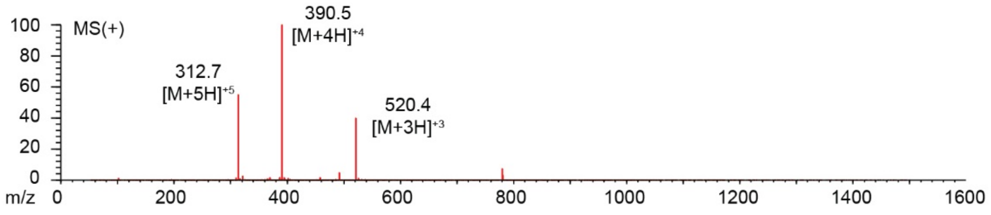

Supplementary Fig. 40. Synthesis summary of **12c PFS**-STCHANGCGGKKK

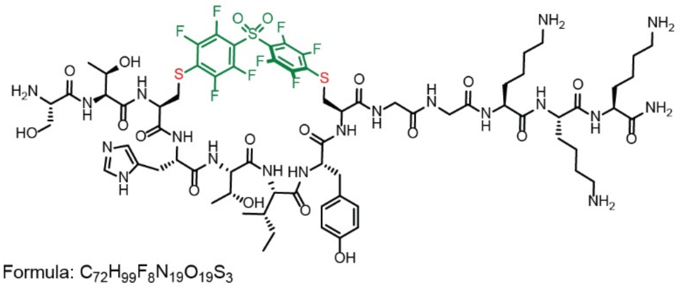

Chemical Formula: $C_{72}H_{99}F_8N_{19}O_{19}S_3$
Exact Mass: 1781.64
Molecular Weight: 1782.87

Starting material mass: 9.0 mg
Final product mass: 1.0 mg

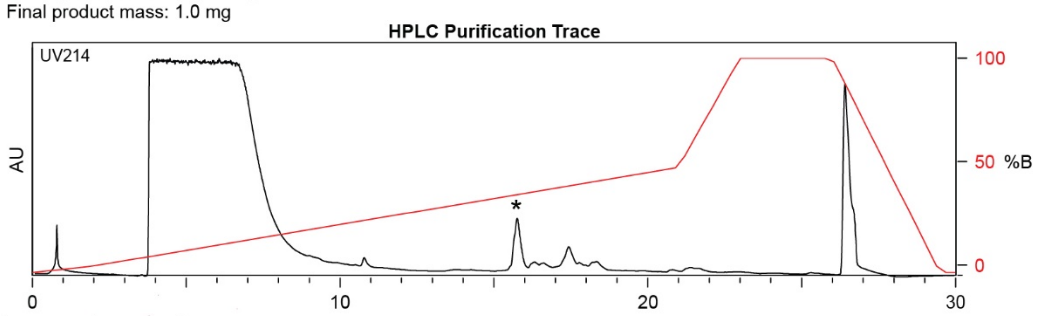

Time (min)	Solvent B (%)
0	2
2	5
21	50
25	100
27	100
28	2
30	2

Flow Rate: 13 mL / min
Phenomenex Kinetex EVO C18 Prep Column
(100 Å, 5 µm, 21.5 mm X 250 mm)

Slovent A: H_2O + 0.1% (v/v) TFA
Slovent B: MeCN + 0.1% (v/v) TFA

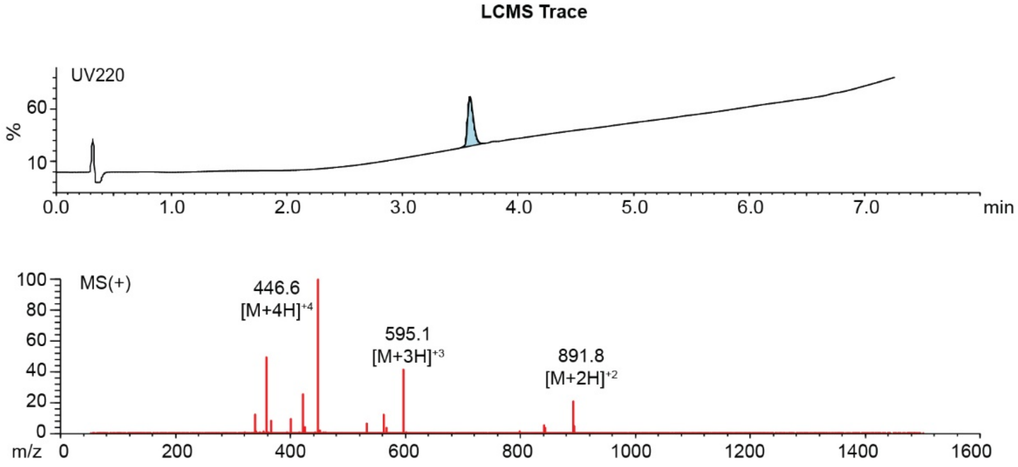

Supplementary Fig. 41. Synthesis summary of **13b DFS**-STCHTIYCGGKKK

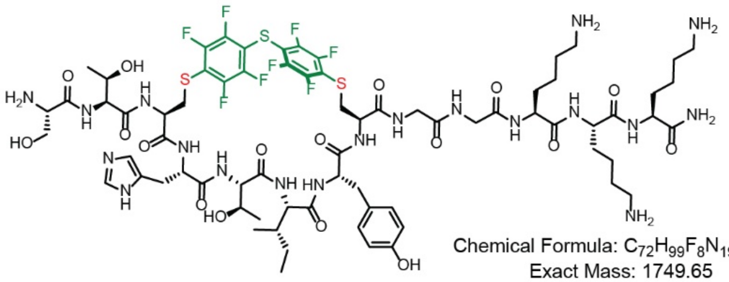

Chemical Formula: $C_{72}H_{99}F_8N_{19}O_{17}S_3$
Exact Mass: 1749.65
Molecular Weight: 1750.87

Starting material mass: 10 mg
Final product mass: 1.4 mg

HPLC Purification Trace

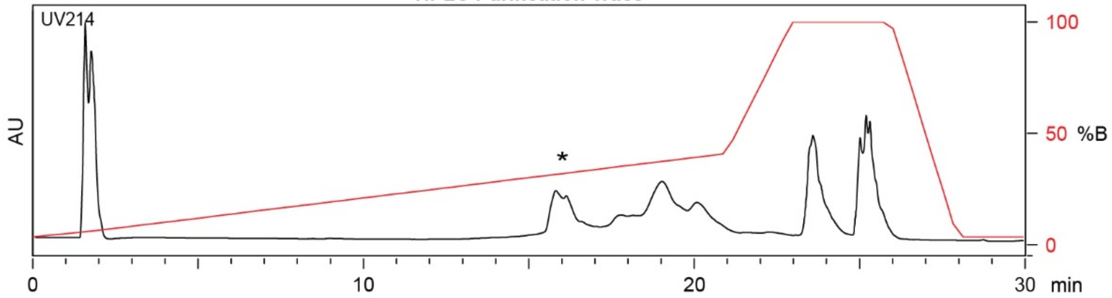

Time (min)	Solvent B (%)
0	2
2	5
21	70
23	100
26	100
29.5	2
30	2

Flow Rate: 13 mL / min
Phenomenex Kinetex EVO C18 Prep Column
(100 Å, 5 µm, 21.5 mm X 250 mm)

Solvent A: H_2O + 0.1% (v/v) TFA
Solvent B: MeCN + 0.1% (v/v) TFA

LCMS Trace

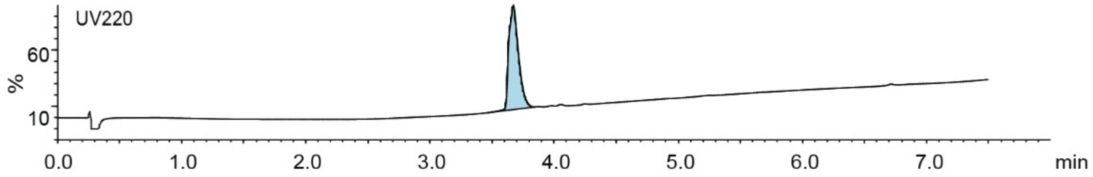

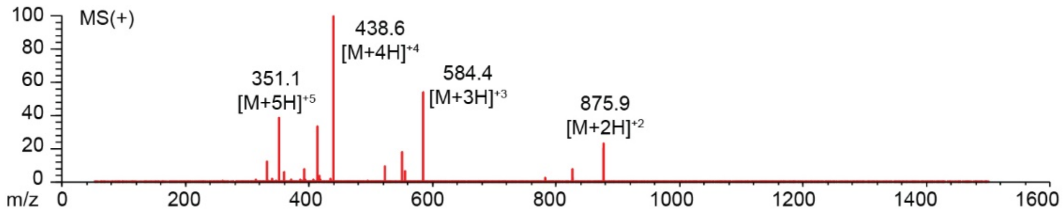

Supplementary Fig. 42. Synthesis summary of **13c PFS**-STCHTIYCGGKKK

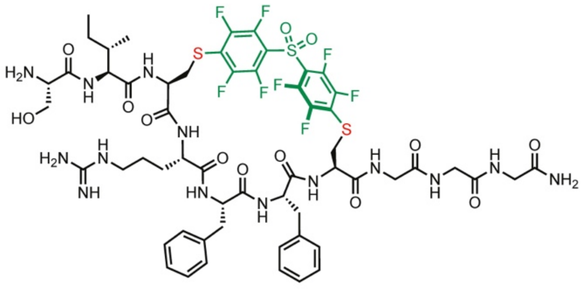

Chemical Formula: $C_{57}H_{66}F_8N_{14}O_{13}S_3$
Exact Mass: 1402.3968
Molecular Weight: 1403.4072

Starting material mass:10 mg
Final product mass: 7.9 mg

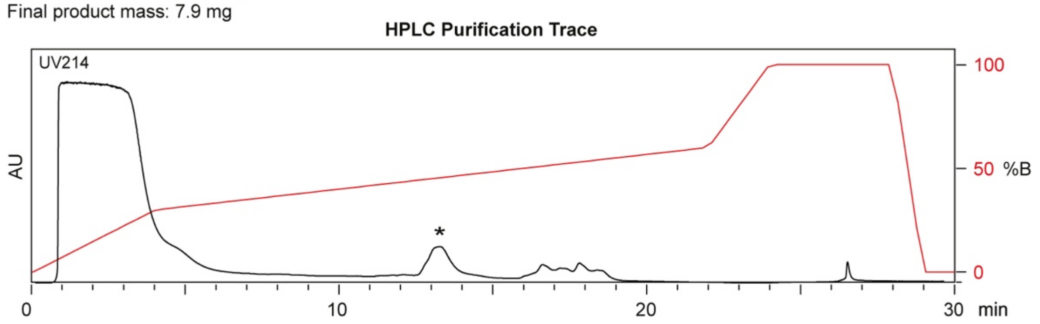

Time (min)	Solvent B (%)
0	0
4	30
22	60
24	100
28	100
29	0
30	0

Flow Rate: 13 mL / min
Phenomenex Kinetex EVO C18 Prep Column
(100 Å, 5 µm, 21.5 mm X 250 mm)

Solvent A: H_2O + 0.1% (v/v) TFA
Solvent B: MeCN + 0.1% (v/v) TFA

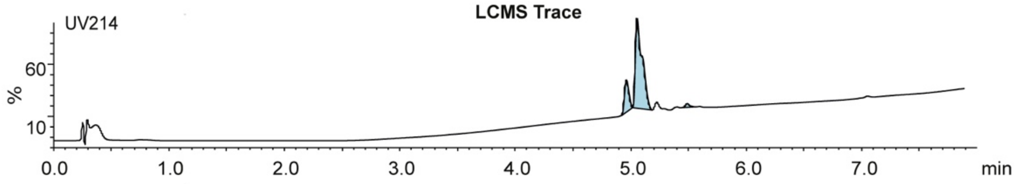

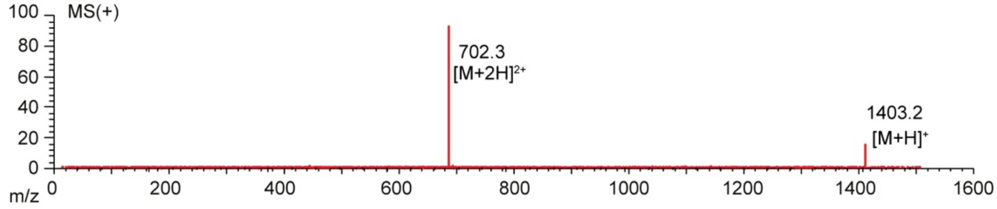

Supplementary Fig. 43. Synthesis summary of **14b DFS**-SICRFFCGGG

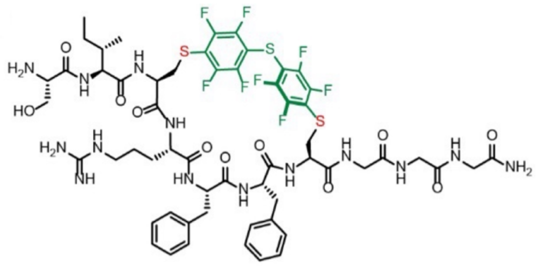

Chemical Formula: $C_{57}H_{66}F_8N_{14}O_{11}S_3$
Exact Mass: 1370.41
Molecular Weight: 1371.41

Starting material mass : 5.5 mg
Final product mass: 3.2 mg

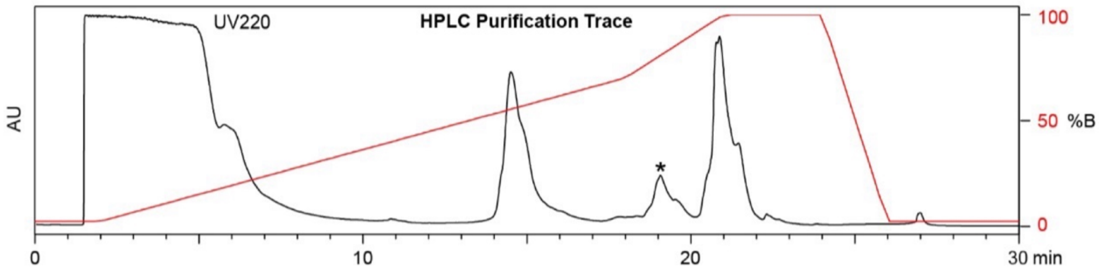

Time (min)	Solvent B (%)
0	2
2	2
18	70
21	100
24	100
26	0
30	0

Flow Rate: 8 mL/min
Symmetry C18 Prep Column
(100 Å, 5 µm, 10 mm X 50 mm)

Slovent A: H_2O + 0.1% (v/v) TFA
Slovent B: MeCN + 0.1% (v/v) TFA

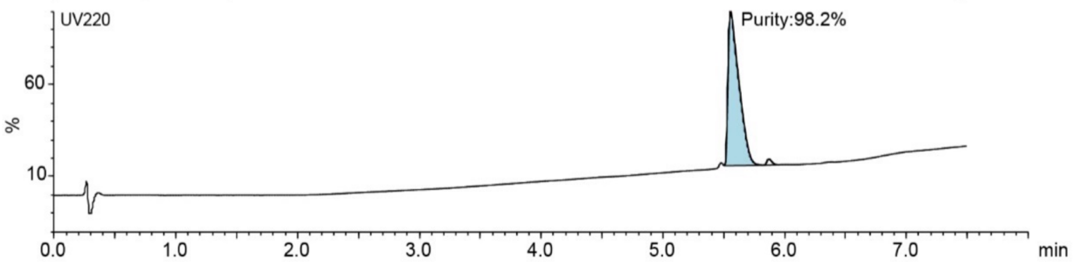

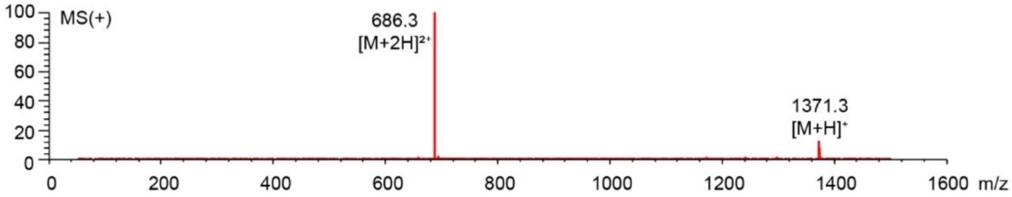

Supplementary Fig. 44. Synthesis summary of **14c PFS**-SICRFFCGGG

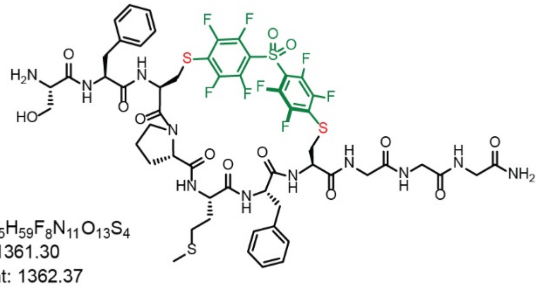

Chemical Formula: $C_{55}H_{59}F_8N_{11}O_{13}S_4$
Exact Mass: 1361.30
Molecular Weight: 1362.37

Starting material mass: 4.7 mg
Final product mass: 2.1 mg

HPLC Purification Trace

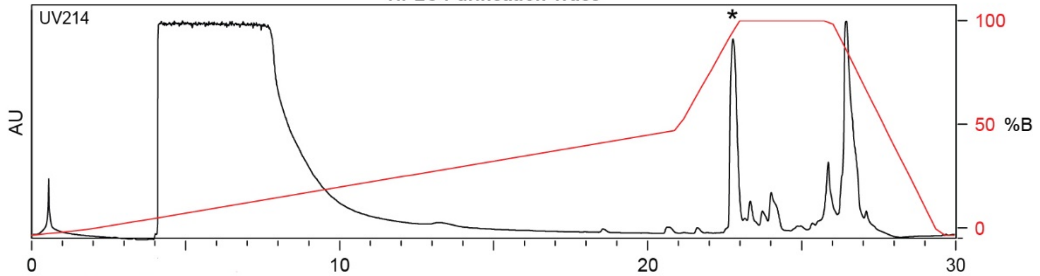

Time (min)	Solvent B (%)
0	2
2	5
21	50
25	100
27	100
28	2
30	2

Flow Rate: 13 mL / min
Phenomenex Kinetex EVO C18 Prep Column
(100 Å, 5 µm, 21.5 mm X 250 mm)

Slovent A: H_2O + 0.1% (v/v) TFA
Slovent B: MeCN + 0.1% (v/v) TFA

LCMS Trace

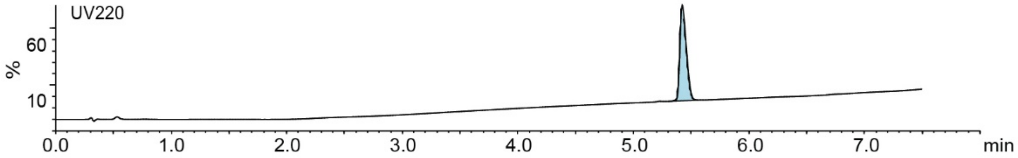

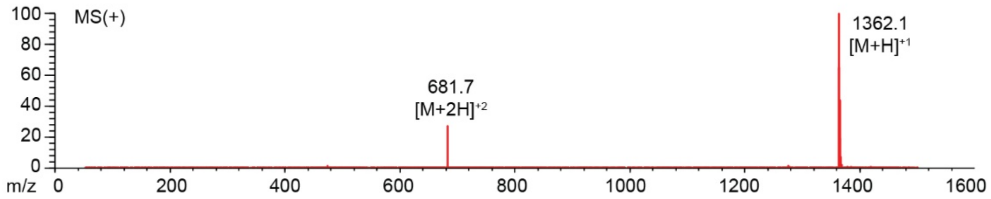

Supplementary Fig. 45. Synthesis summary of **15b DFS**-SFCPMFGGG

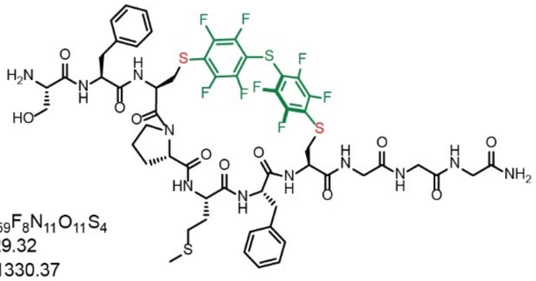

Chemical Formula: $C_{55}H_{59}F_8N_{11}O_{11}S_4$
Exact Mass: 1329.32
Molecular Weight: 1330.37

Starting material mass: 10 mg
Final product mass: 5.1 mg

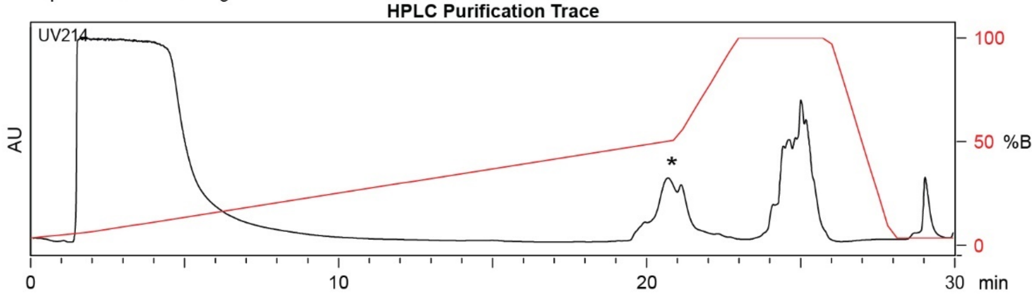

Time (min)	Solvent B (%)
0	2
2	5
21	50
23	100
26	100
29.5	2
30	2

Flow Rate: 13 mL / min
Phenomenex Kinetex EVO C18 Prep Column
(100 Å, 5 µm, 21.5 mm X 250 mm)

Solvent A: H_2O + 0.1% (v/v) TFA
Solvent B: MeCN + 0.1% (v/v) TFA

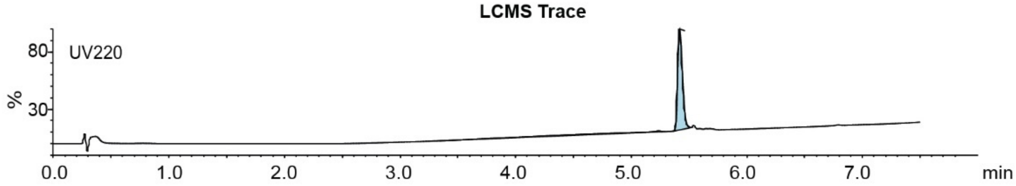

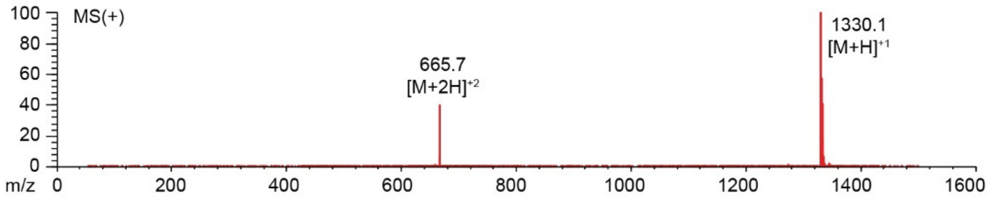

Supplementary Fig. 46. Synthesis summary of **15c PFS**-SFCPMFGGG

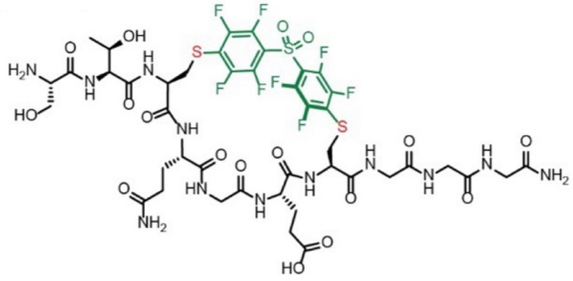

Chemical Formula: $C_{43}H_{50}F_8N_{12}O_{17}S_3$
Exact Mass: 1254.25
Molecular Weight: 1255.11

Starting material mass: 10 mg
Final product mass: 9.4 mg

HPLC Purification Trace

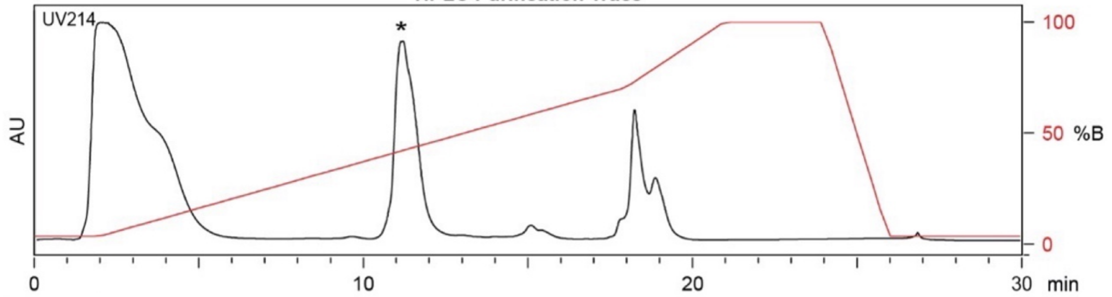

Time (min)	Solvent B (%)
0	2
2	5
21	70
23	100
26	100
29.5	2
30	2

Flow Rate: 13 mL / min
Phenomenex Kinetex EVO C18 Prep Column
(100 Å, 5 µm, 21.5 mm X 250 mm)

Solvent A: H_2O + 0.1% (v/v) TFA
Solvent B: MeCN + 0.1% (v/v) TFA

LCMS Trace

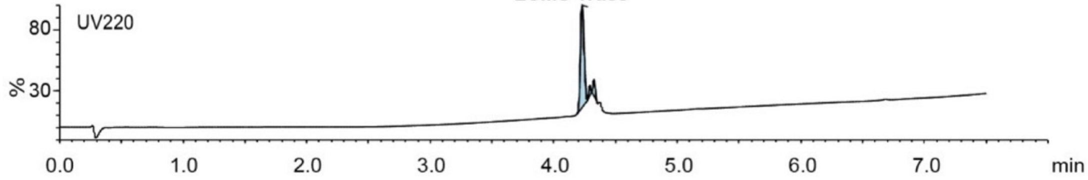

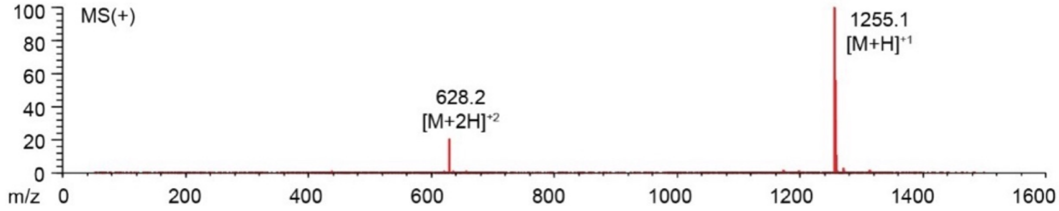

Supplementary Fig. 47. Synthesis summary of **16b DFS**-SLCKRECGGG

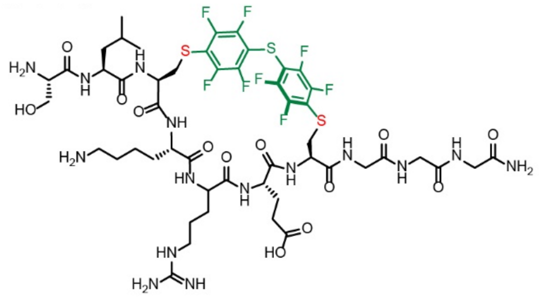

Chemical Formula: $C_{50}H_{67}F_8N_{15}O_{13}S_3$
Exact Mass: 1333.41
Molecular Weight: 1334.35

Starting material mass : 10 mg
Final product mass: 2.8 mg

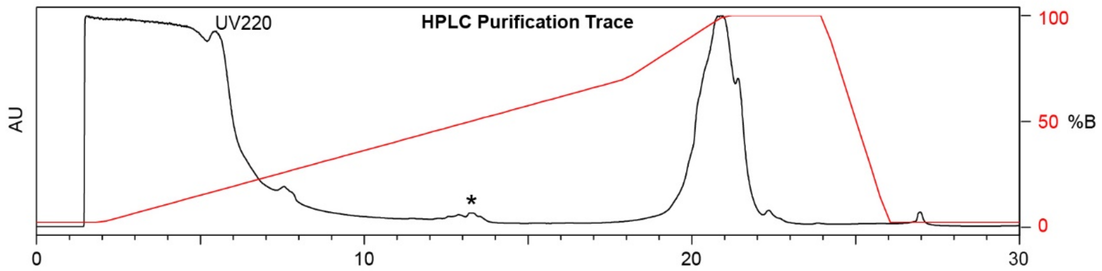

Time (min)	Solvent B %
0	2
2	2
18	70
21	100
24	100
26	0
30	0

Flow Rate: 8 mL/min
Symmetry C18 Prep Column
(100 Å, 5 µm, 10 mm X 50 mm)

Slovent A: H_2O + 0.1% (v/v) TFA
Slovent B: MeCN + 0.1% (v/v) TFA

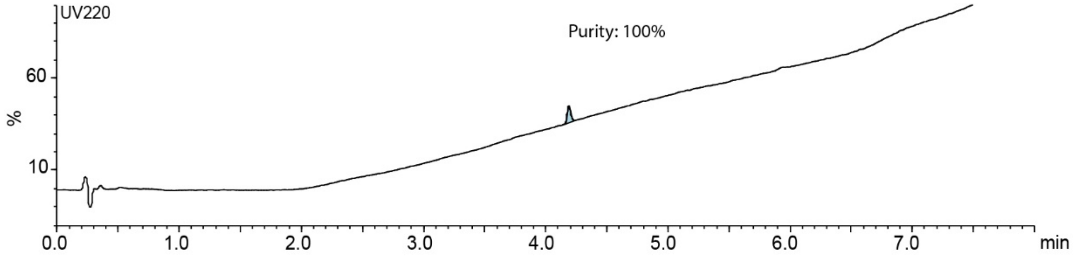

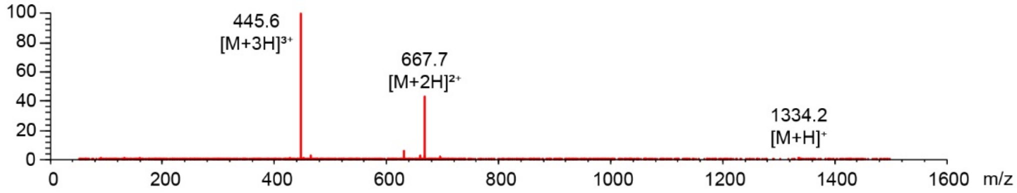

Supplementary Fig. 48. Synthesis summary of **16c PFS**-SLCKRECGGG

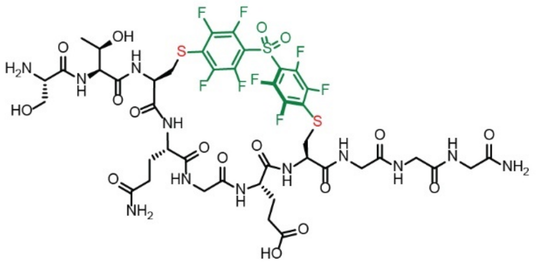

Chemical Formula: $C_{43}H_{50}F_8N_{12}O_{17}S_3$
Exact Mass: 1254.25
Molecular Weight: 1255.11

Starting material mass: 10 mg
Final product mass: 9.4 mg

HPLC Purification Trace

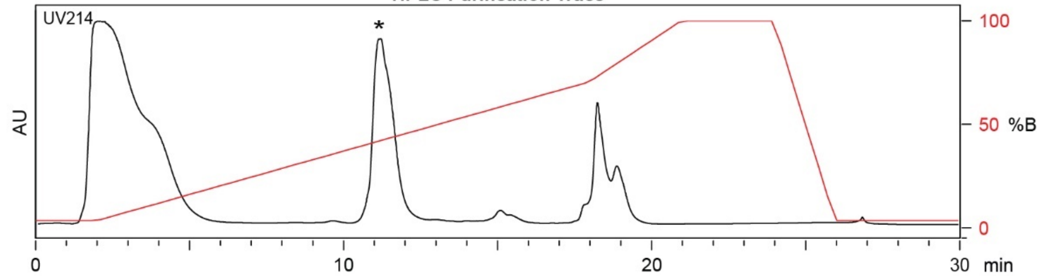

Time (min)	Solvent B (%)
0	2
2	5
21	70
23	100
26	100
29.5	2
30	2

Flow Rate: 13 mL / min
Phenomenex Kinetex EVO C18 Prep Column
(100 Å, 5 µm, 21.5 mm X 250 mm)

Solvent A: H_2O + 0.1% (v/v) TFA
Solvent B: MeCN + 0.1% (v/v) TFA

LCMS Trace

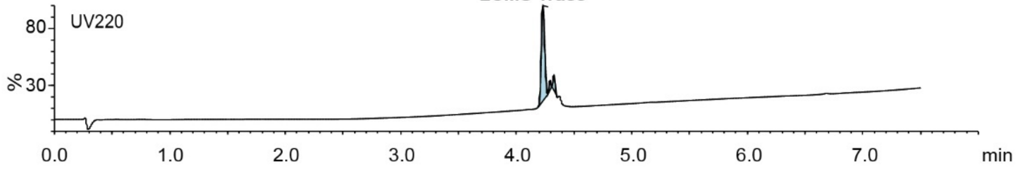

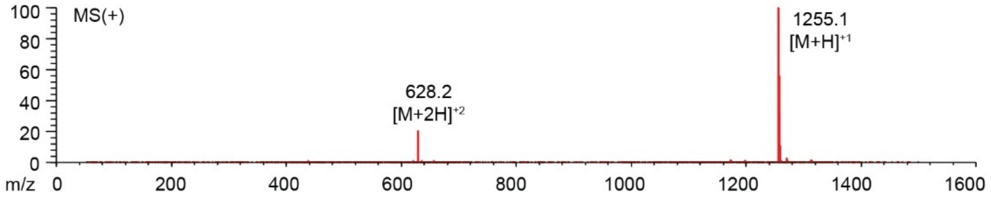

Supplementary Fig. 49. Synthesis summary of **17b DFS**-STCQGECGGG

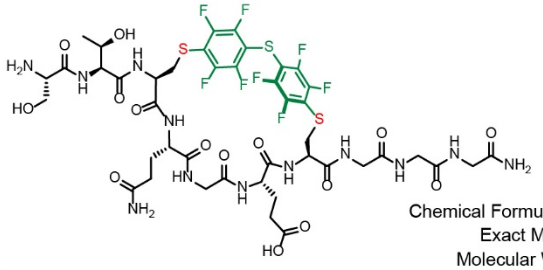

Chemical Formula: $C_{43}H_{50}F_8N_{12}O_{15}S_3$
Exact Mass: 1222.26
Molecular Weight: 1223.11

Starting material mass : 9.7 mg
Final product mass: 5.0 mg

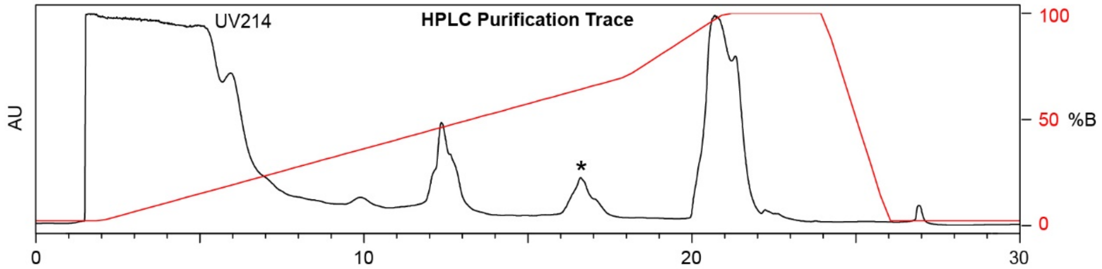

Time (min)	Solvent B %
0	2
2	2
18	70
21	100
24	100
26	0
30	0

Flow Rate: 8 mL/min
Symmetry C18 Prep Column
(100 Å, 5 µm, 10 mm X 50 mm)

Slovent A: H_2O + 0.1% (v/v) TFA
Slovent B: MeCN + 0.1% (v/v) TFA

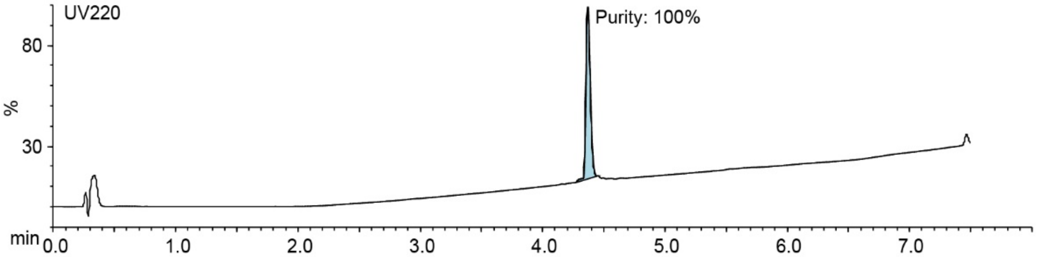

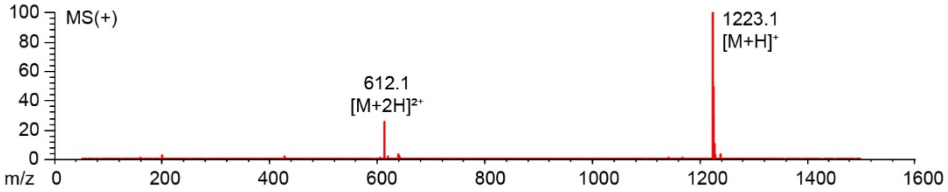

Supplementary Fig. 50. Synthesis summary of **17c PFS**-STCQGECGGG

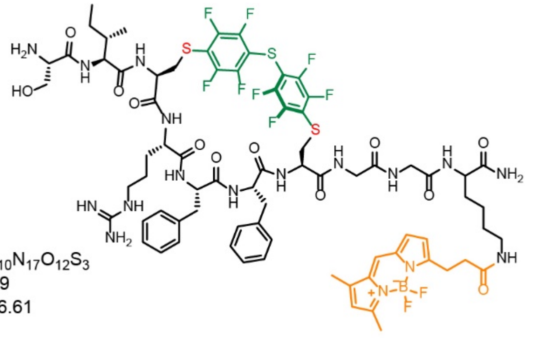

Chemical Formula: $C_{75}H_{88}BF_{10}N_{17}O_{12}S_3$
Exact Mass: 1715.59
Molecular Weight: 1716.61

Starting material mass : 1.2 mg
Final product mass: >0.1 mg

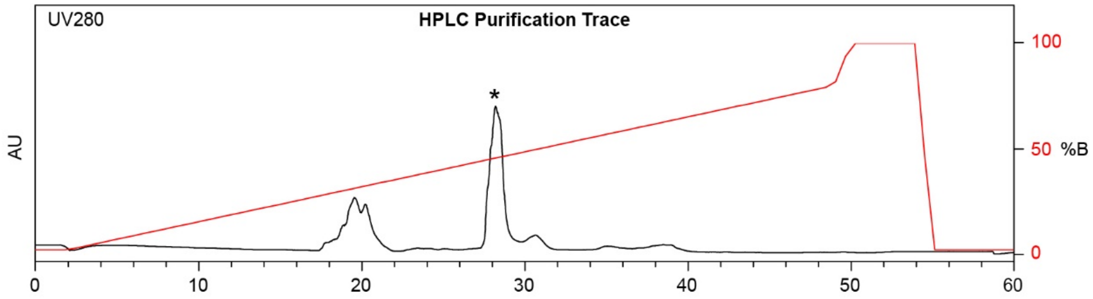

Time (min)	Solvent B %
0	2
2	2
49	80
50	100
54	100
55	2
60	2

Flow Rate: 8 mL/min
Symmetry C18 Prep Column
(100 Å, 5 µm, 10 mm X 50 mm)

Slovent A: H_2O + 0.1% (v/v) TFA
Slovent B: MeCN + 0.1% (v/v) TFA

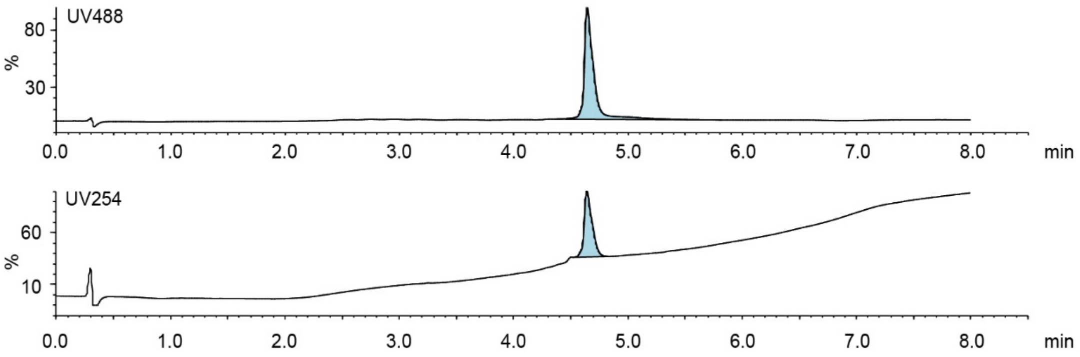

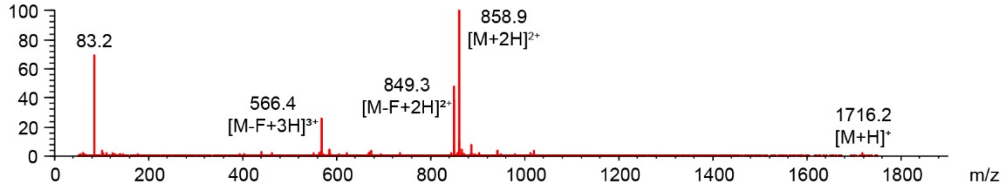

Supplementary Fig. 51. Synthesis summary of **PFS-**SICRFFGGG-BODIPY (**18d**)

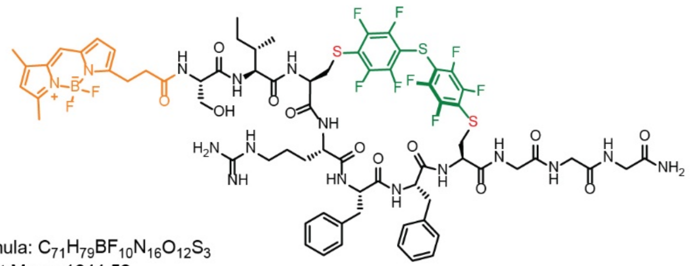

Chemical Formula: $C_{71}H_{79}BF_{10}N_{16}O_{12}S_3$
Exact Mass: 1644.52
Molecular Weight: 1645.49

Starting material mass: 1.6 mg
Final product mass: 1 mg

HPLC Purification Trace

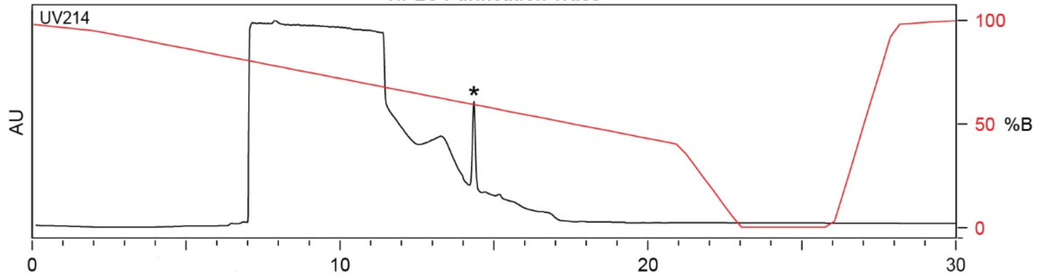

Time (min)	Solvent B (%)
0	98
2	95
21	40
23	0
26	0
28	98
30	98

Flow Rate: 8 mL / min
XBridge BEH Amide OBD Prep Column
(130 Å, 5 μm, 19 mm X 250 mm)

Slovent A: H_2O + 0.1% (v/v) TFA
Slovent B: MeCN + 0.1% (v/v) TFA

LCMS Trace

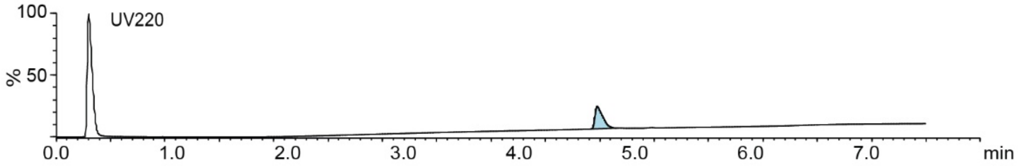

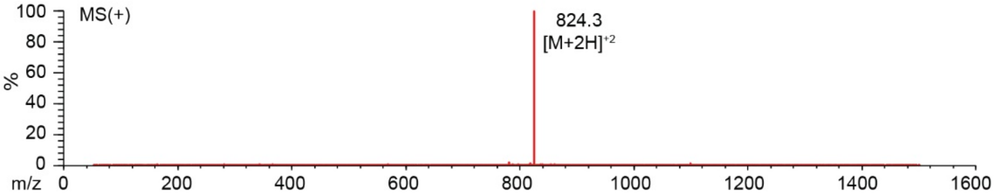

Supplementary Fig. 52. Synthesis summary of BODIPY-**PFS**-SICRFFGGG (**14e**)

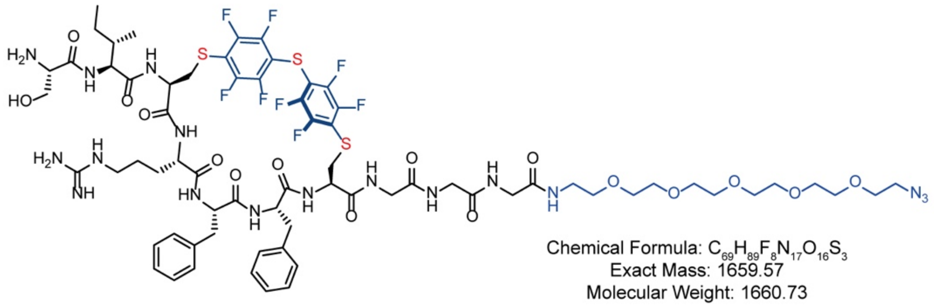

Chemical Formula: $C_{69}H_{89}F_8N_{17}O_{16}S_3$
Exact Mass: 1659.57
Molecular Weight: 1660.73

Starting material mass: 5 mg
Final product mass: 2 mg

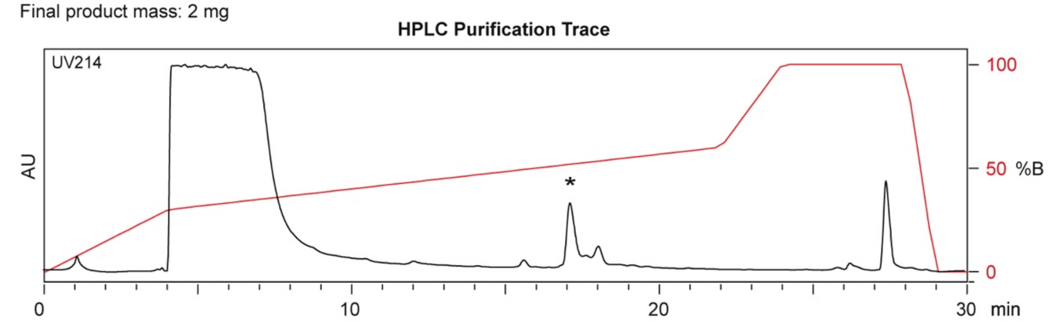

Time (min)	Solvent B (%)
0	0
4	30
22	60
24	100
28	100
29	0
30	0

Flow Rate: 13 mL / min
Phenomenex Kinetex EVO C18 Prep Column
(100 Å, 5 µm, 21.5 mm X 250 mm)

Solvent A: H_2O + 0.1% (v/v) TFA
Solvent B: MeCN + 0.1% (v/v) TFA

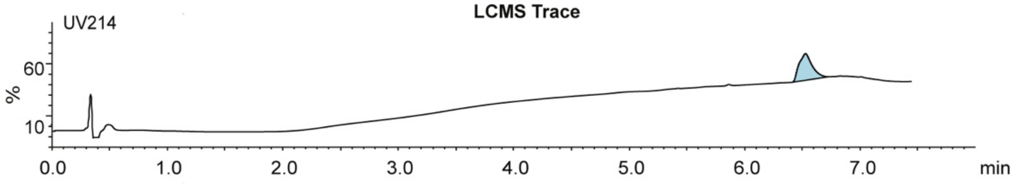

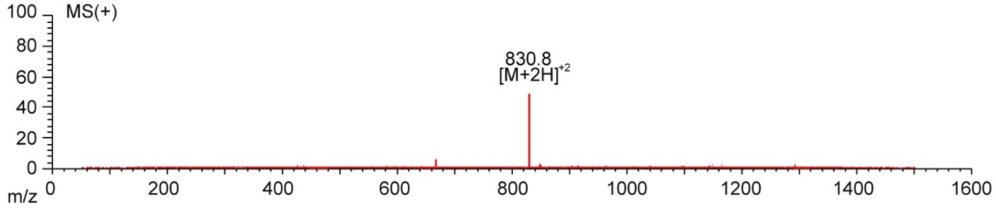

Supplementary Fig. 53. Synthesis summary of **PFS**-SICRFFGGG-PEG4 (**14i**).

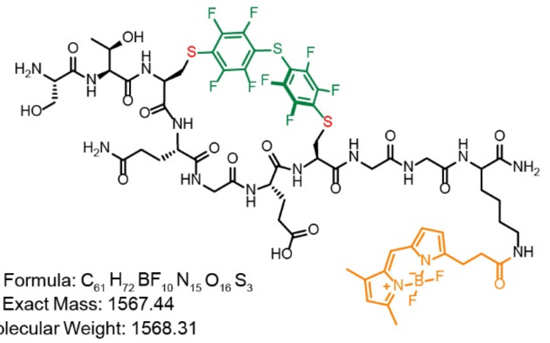

Chemical Formula: $C_{61}H_{72}BF_{10}N_{15}O_{16}S_3$
Exact Mass: 1567.44
Molecular Weight: 1568.31

Starting material mass : 1.2 mg
Final product mass: >0.1 mg

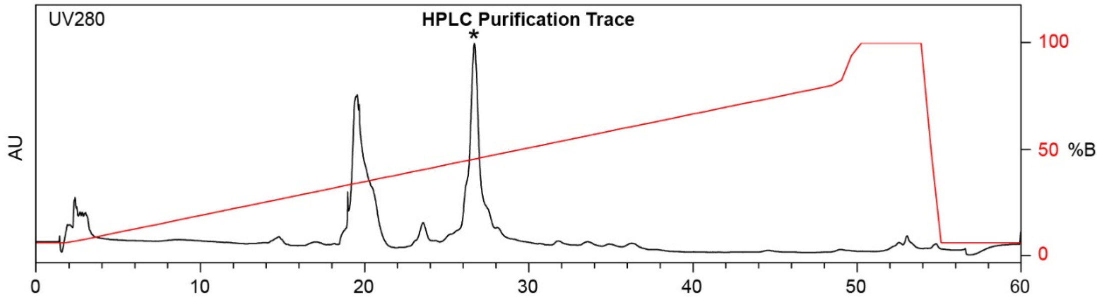

Time (min)	Solvent B %
0	2
2	2
49	80
50	100
54	100
55	2
60	2

Flow Rate: 8 mL/min
Symmetry C18 Prep Column
(100 Å, 5 μm, 10 mm X 50 mm)

Slovent A: H_2O + 0.1% (v/v) TFA
Slovent B: MeCN + 0.1% (v/v) TFA

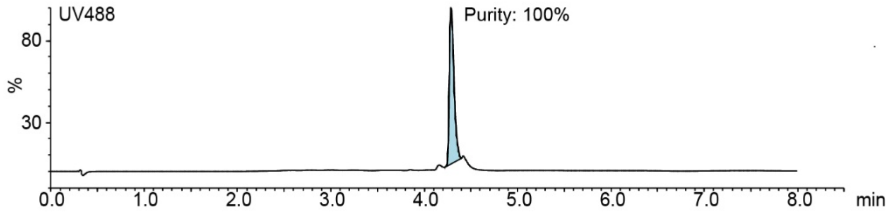

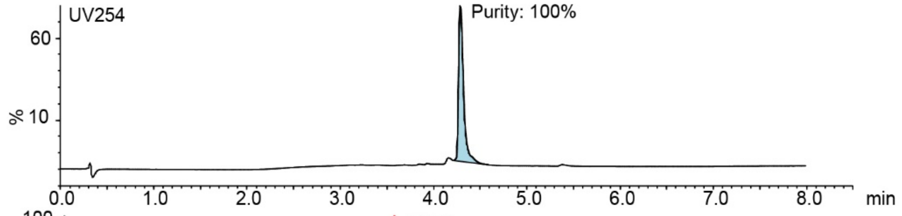

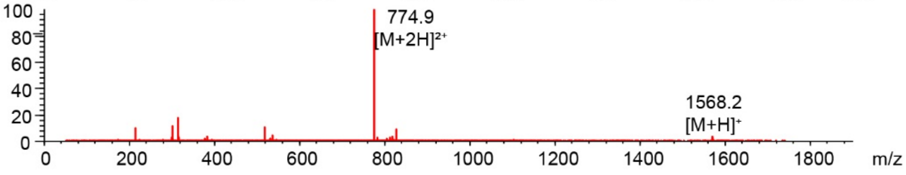

Supplementary Fig. 54. Synthesis summary of **PFS**-STCQGECGGK-BODIPY (**19d**)

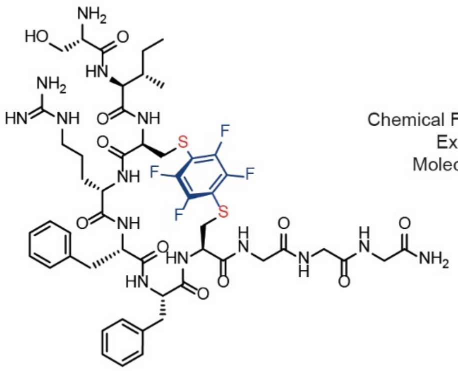

Chemical Formula: $C_{51}H_{66}F_4N_{14}O_{11}S_2$
Exact Mass: 1190.4
Molecular Weight: 1191.5

Starting material mass: 5 mg
Final product mass: 1.5 mg

HPLC Purification Trace

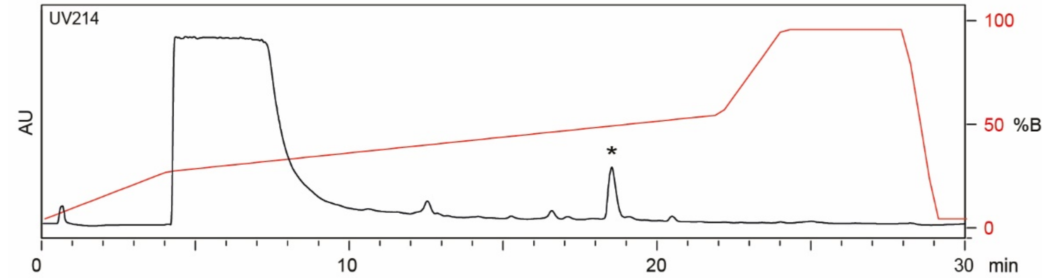

Time (min)	Solvent B (%)
0	0
2	20
21	50
23	100
26	100
29	0
30	0

Flow Rate: 13 mL / min
Phenomenex Kinetex EVO C18 Prep Column
(100 Å, 5 µm, 21.5 mm X 250 mm)

Solvent A: H_2O + 0.1% (v/v) TFA
Solvent B: MeCN + 0.1% (v/v) TFA

LCMS Trace

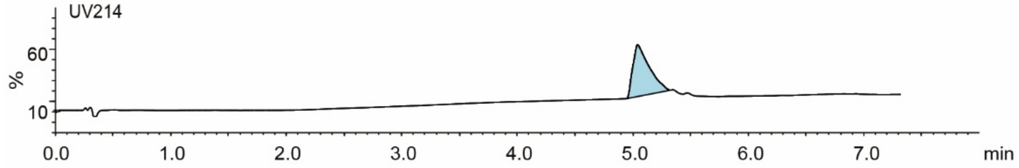

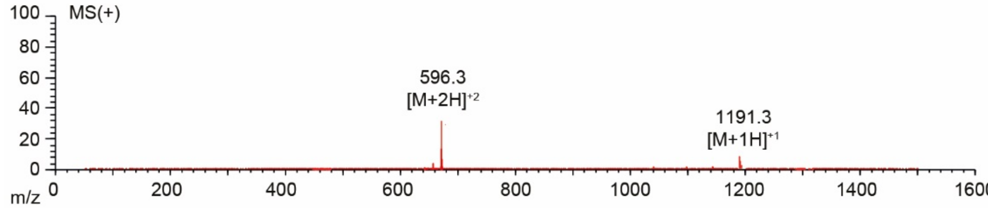

Supplementary Fig. 55. Synthesis summary of HFB-SICRFFGGG (**14j**)

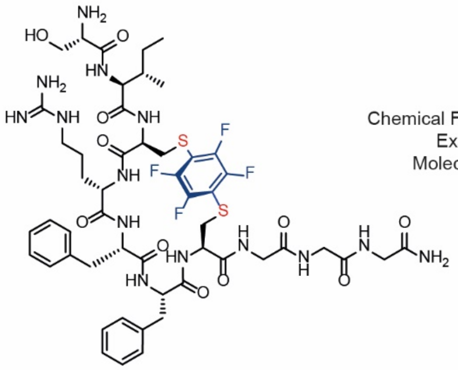

Chemical Formula: $C_{51}H_{66}F_{4}N_{14}O_{11}S_{2}$
Exact Mass: 1190.4
Molecular Weight: 1191.5

Starting material mass: 5 mg
Final product mass: 1.5 mg

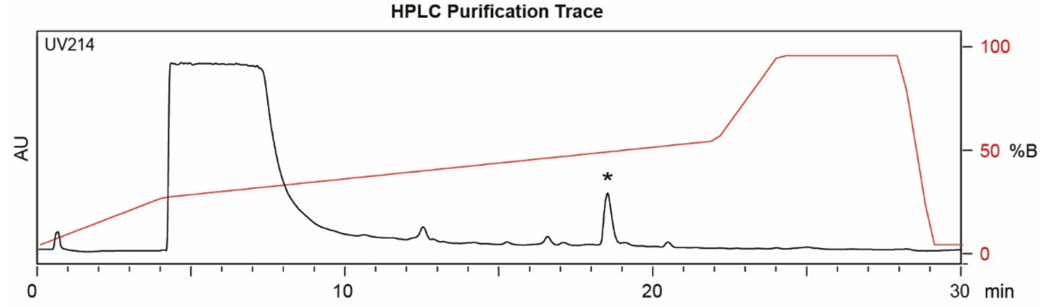

Time (min)	Solvent B (%)
0	0
2	20
21	50
23	100
26	100
29	0
30	0

Flow Rate: 13 mL / min
Phenomenex Kinetex EVO C18 Prep Column
(100 Å, 5 μm, 21.5 mm X 250 mm)

Solvent A: H_2O + 0.1% (v/v) TFA
Solvent B: MeCN + 0.1% (v/v) TFA

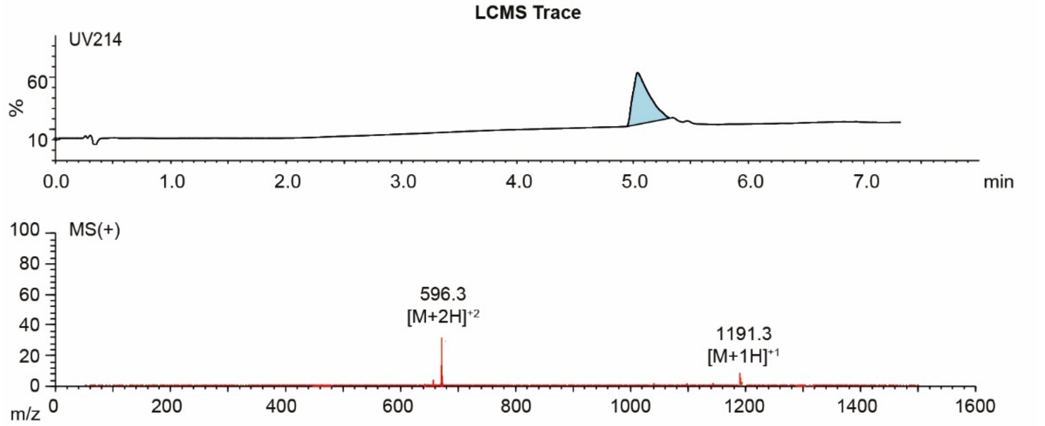

Supplementary Fig. 56. Synthesis summary of DFB-SICRFFGGG (**14k**)

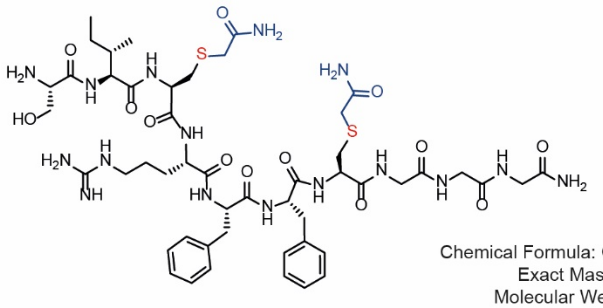

Chemical Formula: $C_{49}H_{74}N_{16}O_{13}S_2$
Exact Mass: 1158.51
Molecular Weight: 1159.34

Starting material mass: 5 mg
Final product mass: 3.4 mg

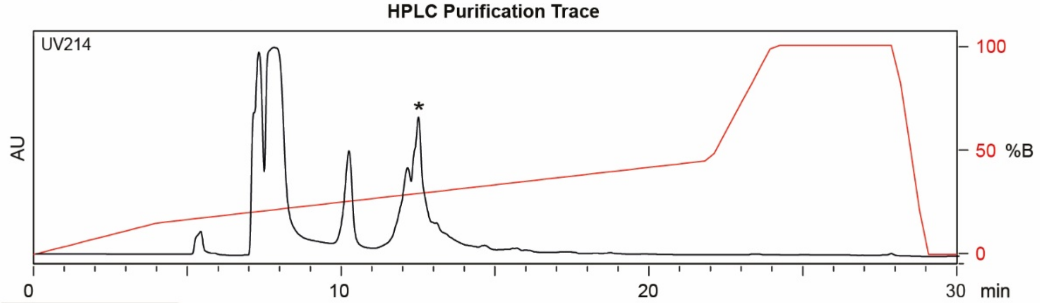

Time (min)	Solvent B (%)
0	0
4	25
22	55
24	100
28	100
29	0
30	0

Flow Rate: 13 mL / min
Phenomenex Kinetex EVO C18 Prep Column
(100 Å, 5 µm, 21.5 mm X 250 mm)

Solvent A: H_2O + 0.1% (v/v) TFA
Solvent B: MeCN + 0.1% (v/v) TFA

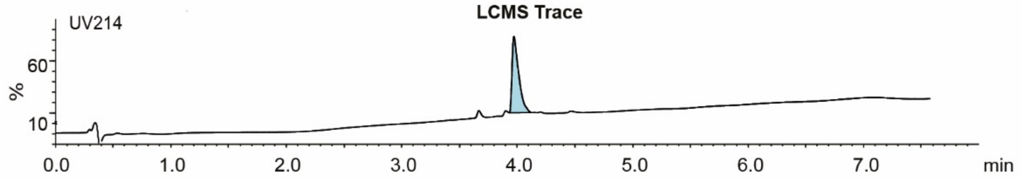

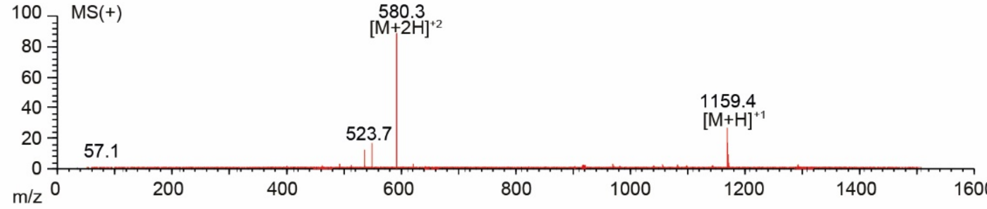

Supplementary Fig. 57. Synthesis summary of IAA-SICRFFGGG (**14l**)

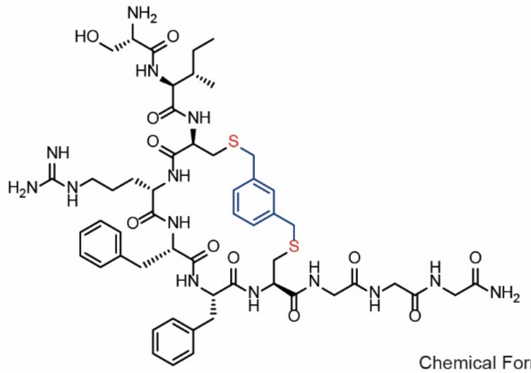

Chemical Formula: $C_{59}H_{74}N_{14}O_{11}S_2$
Exact Mass: 1146.51
Molecular Weight: 1147.37

Starting material mass: 5 mg
Final product mass: 2.8 mg

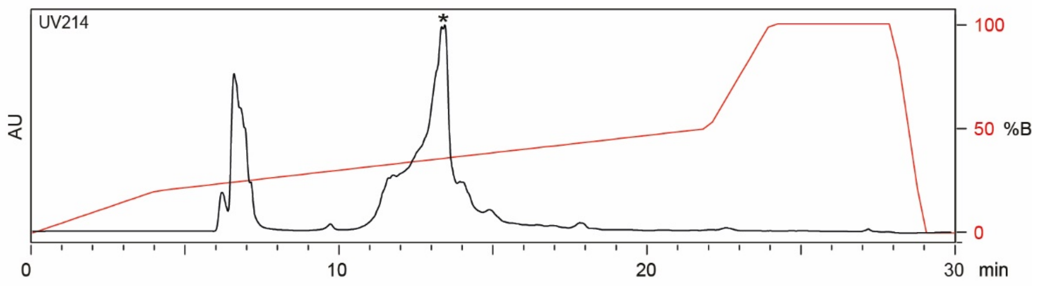

Time (min)	Solvent B (%)
0	0
4	20
22	50
24	100
28	100
29	0
30	0

Flow Rate: 13 mL / min
Phenomenex Kinetex EVO C18 Prep Column
(100 Å, 5 µm, 21.5 mm X 250 mm)

Solvent A: H_2O + 0.1% (v/v) TFA
Solvent B: MeCN + 0.1% (v/v) TFA

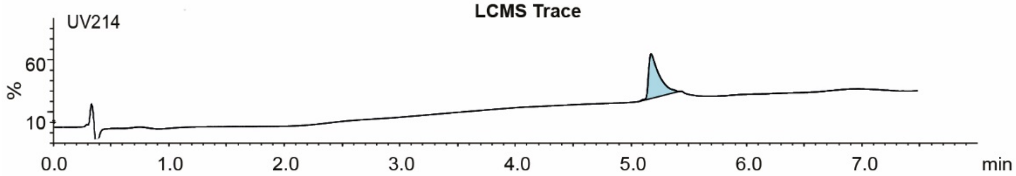

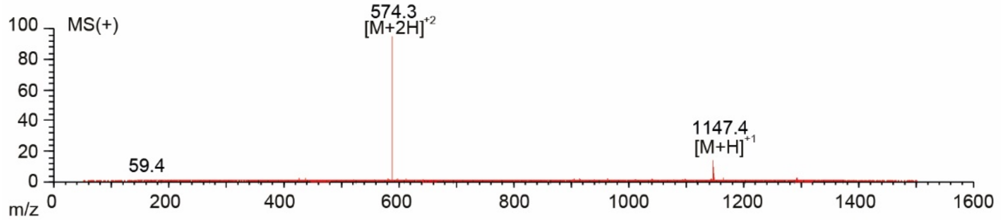

Supplementary Fig. 58. Synthesis summary of MBX-SICRFFGGG (**14m**)

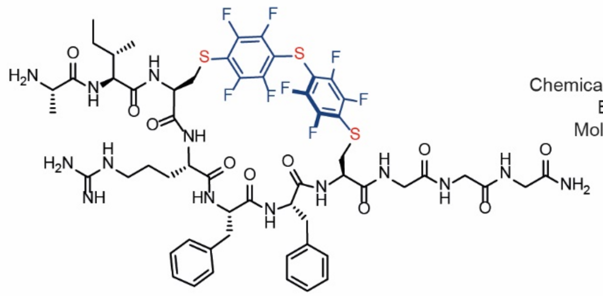

Chemical Formula: $C_{54}H_{60}F_8N_{14}O_{11}S_3$
Exact Mass: 1354.4
Molecular Weight: 1355.4

Starting material mass: 5 mg
Final product mass: 0.8 mg

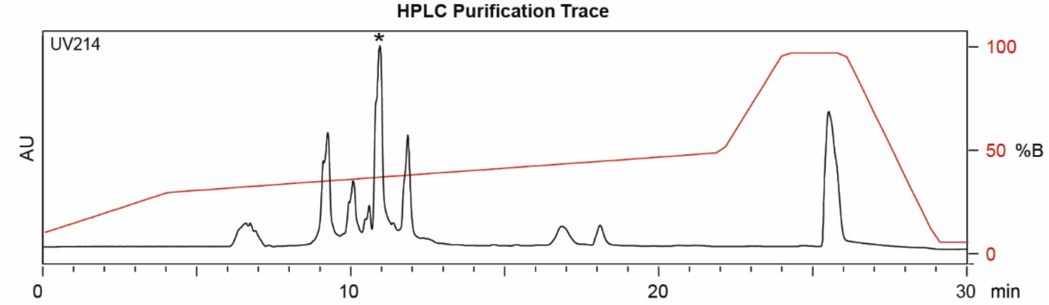

Time (min)	Solvent B (%)
0	10
2	30
21	50
23	100
26	100
29.5	5
30	5

Flow Rate: 13 mL / min
Phenomenex Kinetex EVO C18 Prep Column
(100 Å, 5 µm, 21.5 mm X 250 mm)

Solvent A: H_2O + 0.1% (v/v) TFA
Solvent B: MeCN + 0.1% (v/v) TFA

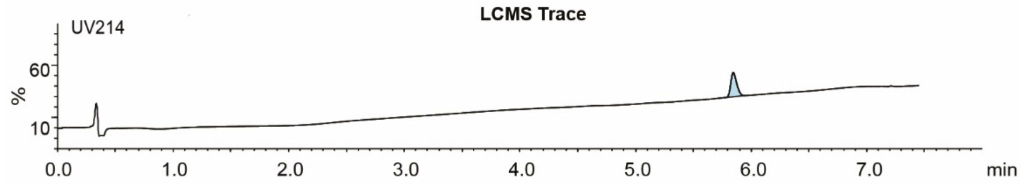

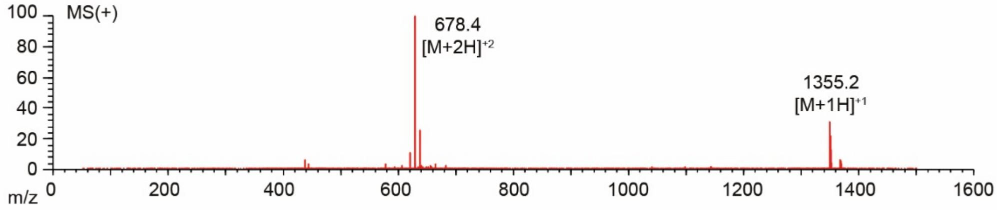

Supplementary Fig. 59. Synthesis summary of PFS-AICRFFGGG (**21c**)

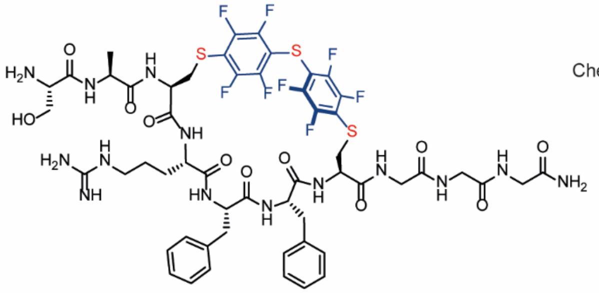

Chemical Formula: $C_{54}H_{60}F_8N_{14}O_{11}S_3$
Exact Mass: 1328.4
Molecular Weight: 1329.2

Starting material mass: 5 mg
Final product mass: 1.3 mg

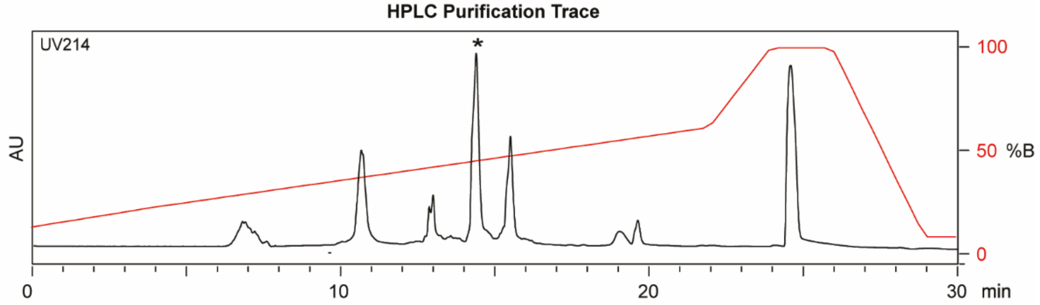

Time (min)	Solvent B (%)
0	10
2	20
21	60
23	100
26	100
29.5	5
30	5

Flow Rate: 13 mL / min
Phenomenex Kinetex EVO C18 Prep Column
(100 Å, 5 µm, 21.5 mm X 250 mm)

Solvent A: H_2O + 0.1% (v/v) TFA
Solvent B: MeCN + 0.1% (v/v) TFA

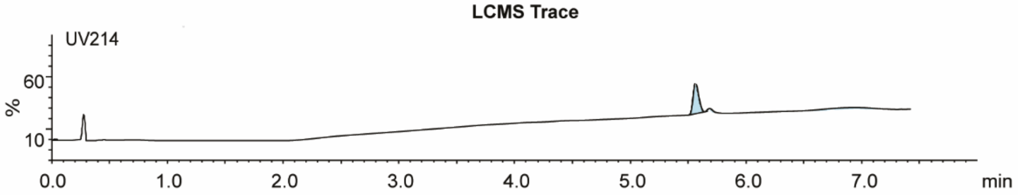

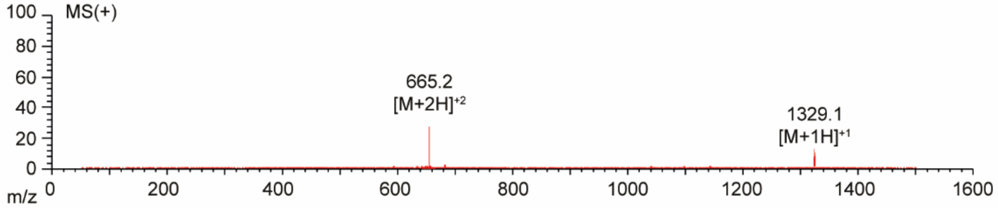

Supplementary Fig. 60. Synthesis summary of PFS-SACRFFGGG (**22c**)

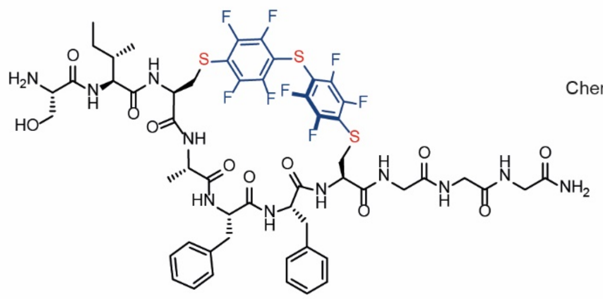

Chemical Formula: $C_{54}H_{59}F_8N_{11}O_{11}S_3$
Exact Mass: 1285.3
Molecular Weight: 1286.3

Starting material mass: 5 mg
Final product mass: 0.3 mg

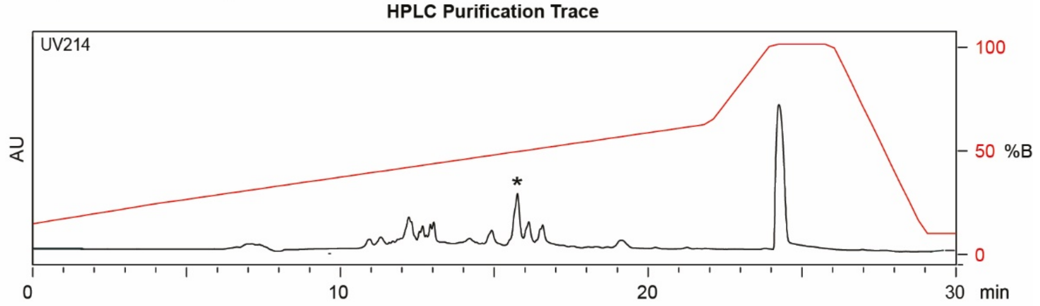

Time (min)	Solvent B (%)
0	10
2	20
21	60
23	100
26	100
29.5	5
30	5

Flow Rate: 13 mL / min
Phenomenex Kinetex EVO C18 Prep Column
(100 Å, 5 µm, 21.5 mm X 250 mm)

Solvent A: H_2O + 0.1% (v/v) TFA
Solvent B: MeCN + 0.1% (v/v) TFA

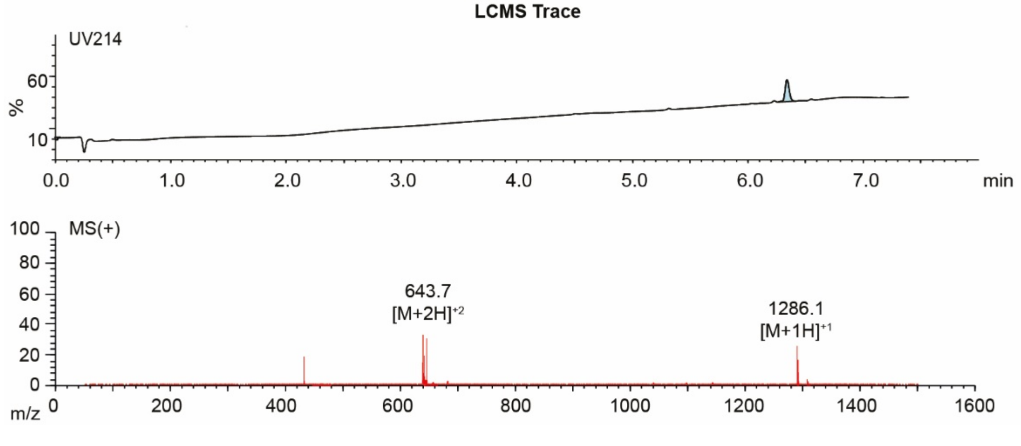

Supplementary Fig. 61. Synthesis summary of PFS-SICAFFGGG (**23c**)

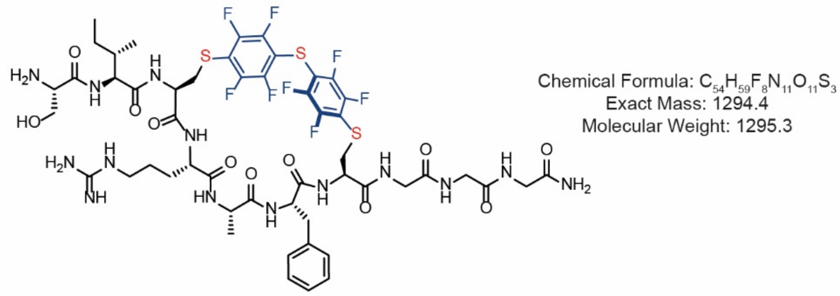

Chemical Formula: $C_{54}H_{59}F_8N_{11}O_{11}S_3$
Exact Mass: 1294.4
Molecular Weight: 1295.3

Starting material mass: 5 mg
Final product mass: 0.8 mg

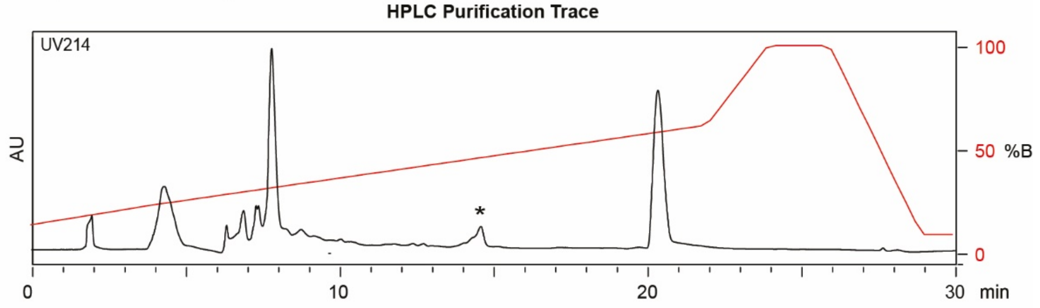

Time (min)	Solvent B (%)
0	10
2	20
21	60
23	100
26	100
29.5	5
30	5

Flow Rate: 13 mL / min
Phenomenex Kinetex EVO C18 Prep Column
(100 Å, 5 µm, 21.5 mm X 250 mm)

Solvent A: H_2O + 0.1% (v/v) TFA
Solvent B: MeCN + 0.1% (v/v) TFA

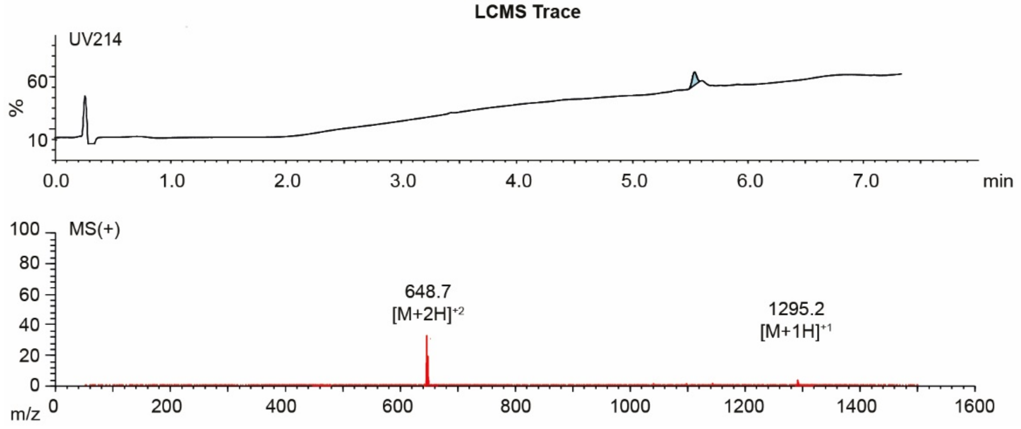

Supplementary Fig. 62. Synthesis summary of PFS-SICRAFGGG (**24c**)

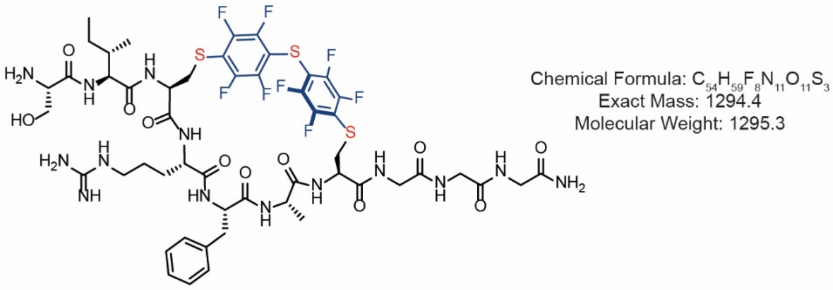

Chemical Formula: $C_{54}H_{59}F_8N_{11}O_{11}S_3$
Exact Mass: 1294.4
Molecular Weight: 1295.3

Starting material mass: 5 mg
Final product mass: 0.6 mg

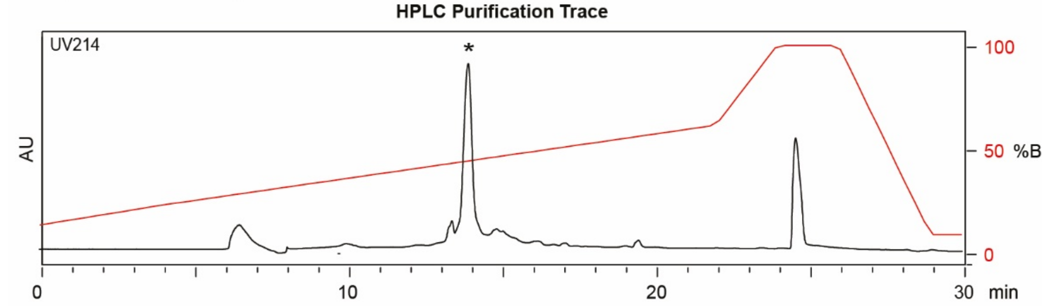

Time (min)	Solvent B (%)
0	10
2	20
21	60
23	100
26	100
29.5	5
30	5

Flow Rate: 13 mL / min
Phenomenex Kinetex EVO C18 Prep Column
(100 Å, 5 µm, 21.5 mm X 250 mm)

Solvent A: H_2O + 0.1% (v/v) TFA
Solvent B: MeCN + 0.1% (v/v) TFA

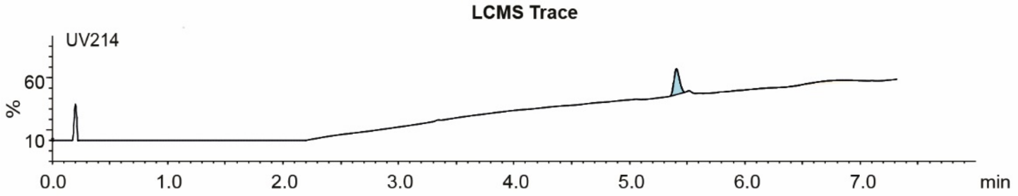

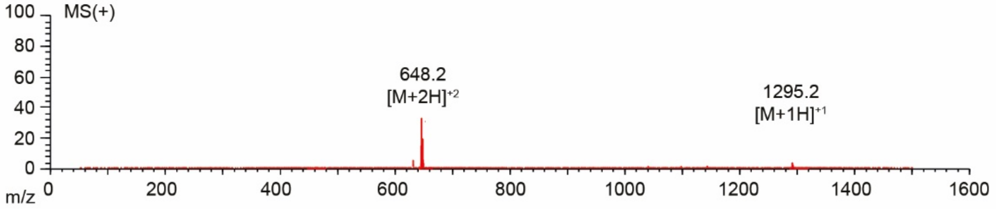

Supplementary Fig. 63. Synthesis summary of PFS-SICRFAGGG (**25c**)

Chemical Formula: $C_{50}H_{73}N_{15}O_{12}S_2$
Exact Mass: 1139.50
Molecular Weight: 1140.3

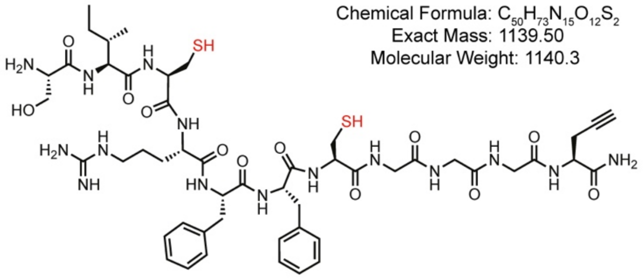

Starting material mass: 200 mg
Final product mass: 50 mg

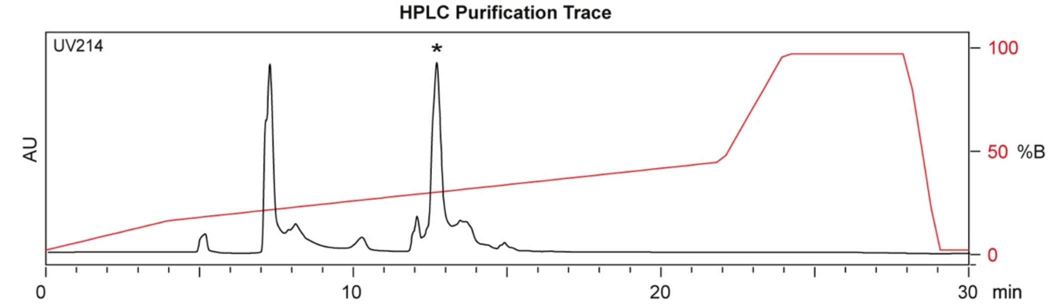

Time (min)	Solvent B (%)
0	0
2	15
22	45
24	100
28	100
29	0
30	0

Flow Rate: 13 mL / min
Phenomenex Kinetex EVO C18 Prep Column
(100 Å, 5 µm, 21.5 mm X 250 mm)

Solvent A: H_2O + 0.1% (v/v) TFA
Solvent B: MeCN + 0.1% (v/v) TFA

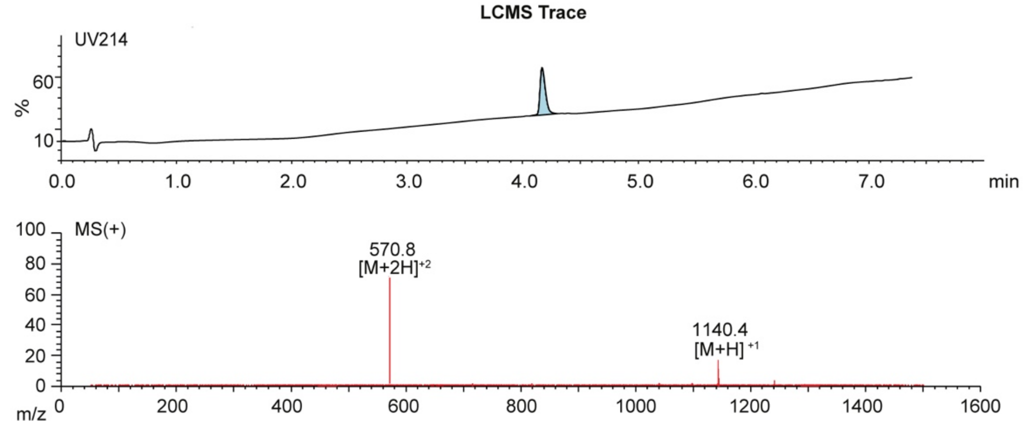

Supplementary Fig. 64. Synthesis summary of SICRFFCGGGZ (**20a**)

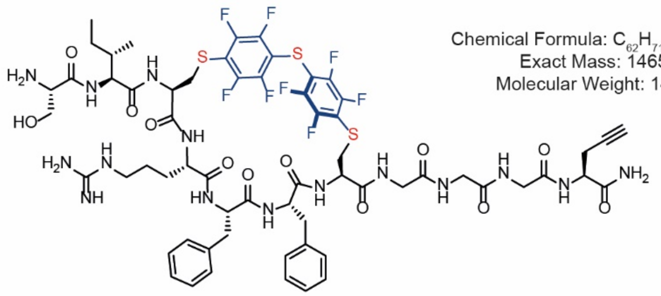

Chemical Formula: $C_{62}H_{71}F_8N_{15}O_{12}S_3$
Exact Mass: 1465.4
Molecular Weight: 1466.5

Starting material mass: 40 mg
Final product mass: 15 mg

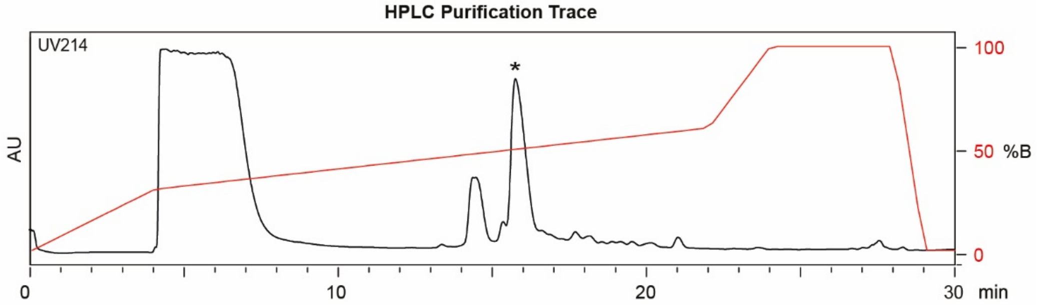

Time (min)	Solvent B (%)
0	0
2	30
21	60
23	100
26	100
29	0
30	0

Flow Rate: 13 mL / min
Phenomenex Kinetex EVO C18 Prep Column
(100 Å, 5 µm, 21.5 mm X 250 mm)

Solvent A: H_2O + 0.1% (v/v) TFA
Solvent B: MeCN + 0.1% (v/v) TFA

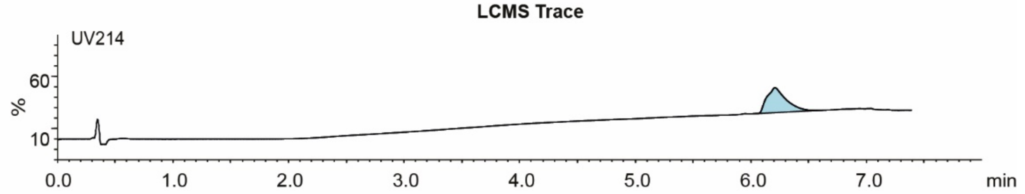

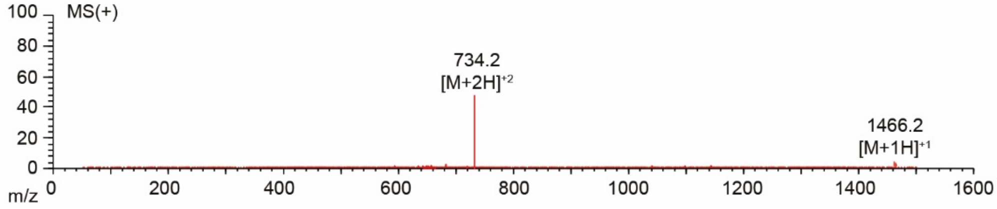

Supplementary Fig. 65. Synthesis summary of **PFS**-SICRFFGGGZ (**20c**)

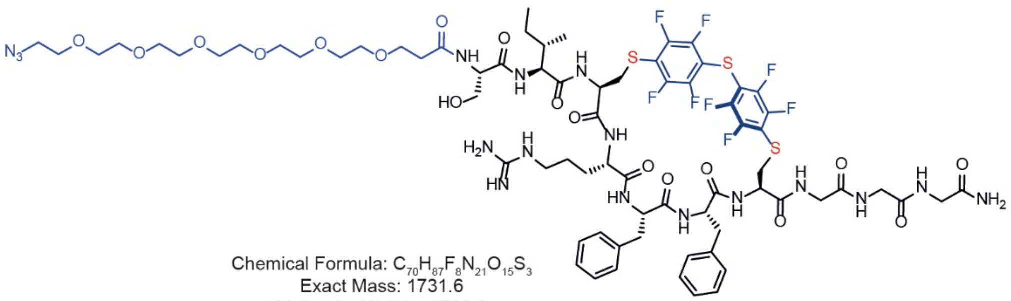

Chemical Formula: $C_{70}H_{87}F_8N_{21}O_{15}S_3$
Exact Mass: 1731.6
Molecular Weight: 1732.8

Starting material mass: 5 mg
Final product mass: 3.1 mg

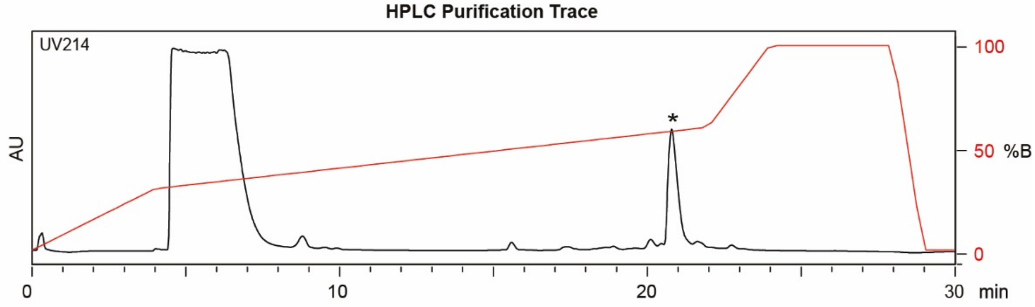

Time (min)	Solvent B (%)
0	0
2	30
21	60
23	100
26	100
29	0
30	0

Flow Rate: 13 mL / min
Phenomenex Kinetex EVO C18 Prep Column
(100 Å, 5 μm, 21.5 mm X 250 mm)

Solvent A: H_2O + 0.1% (v/v) TFA
Solvent B: MeCN + 0.1% (v/v) TFA

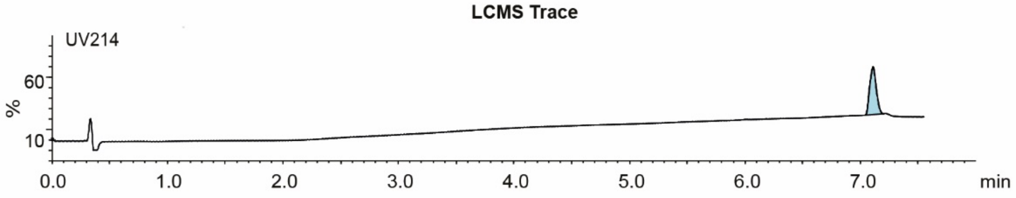

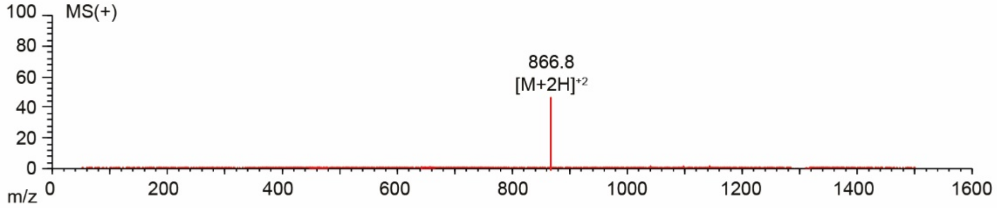

Supplementary Fig. 66. Synthesis summary of Np7-**PFS**-SICRFFGGG (**14g**)

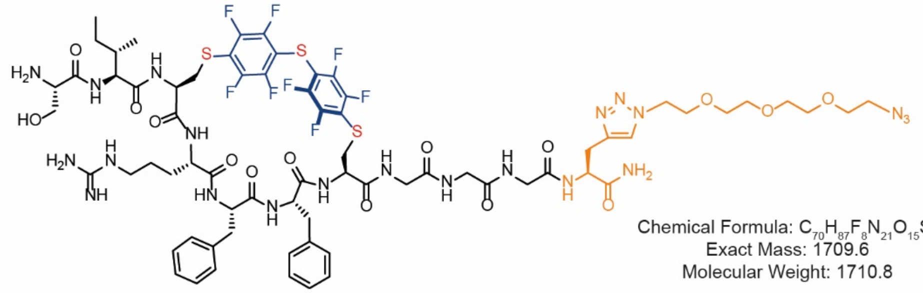

Chemical Formula: $C_{70}H_{87}F_8N_{21}O_{15}S_3$
Exact Mass: 1709.6
Molecular Weight: 1710.8

Starting material mass: 1.5 mg
Final product mass: 0.7 mg

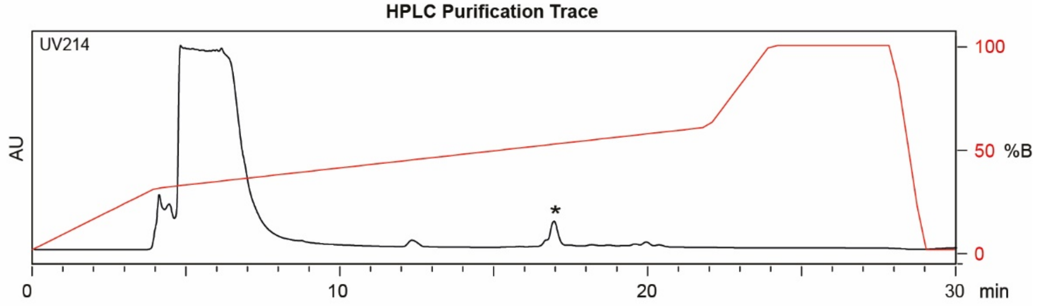

Time (min)	Solvent B (%)
0	0
2	30
21	60
23	100
26	100
29	0
30	0

Flow Rate: 13 mL / min
Phenomenex Kinetex EVO C18 Prep Column
(100 Å, 5 µm, 21.5 mm X 250 mm)

Solvent A: H_2O + 0.1% (v/v) TFA
Solvent B: MeCN + 0.1% (v/v) TFA

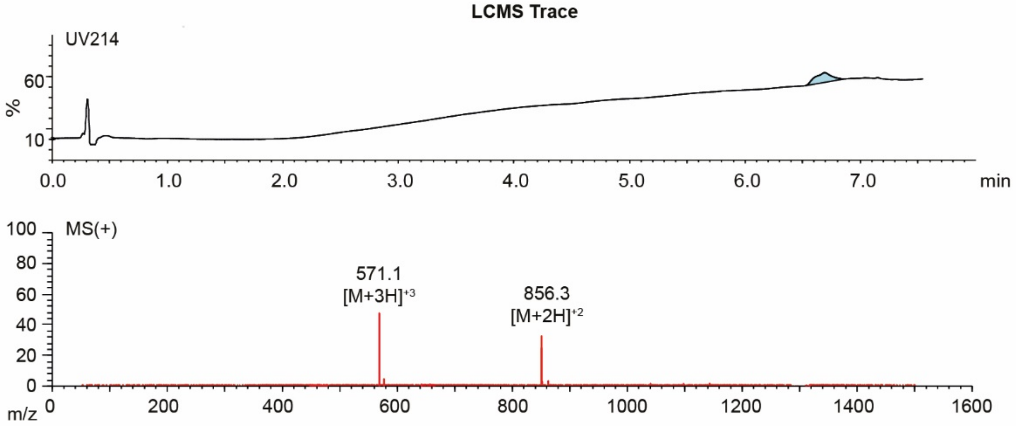

Supplementary Fig. 67. Synthesis summary of **PFS**-SICRFFGGGZp4 (**20h**)

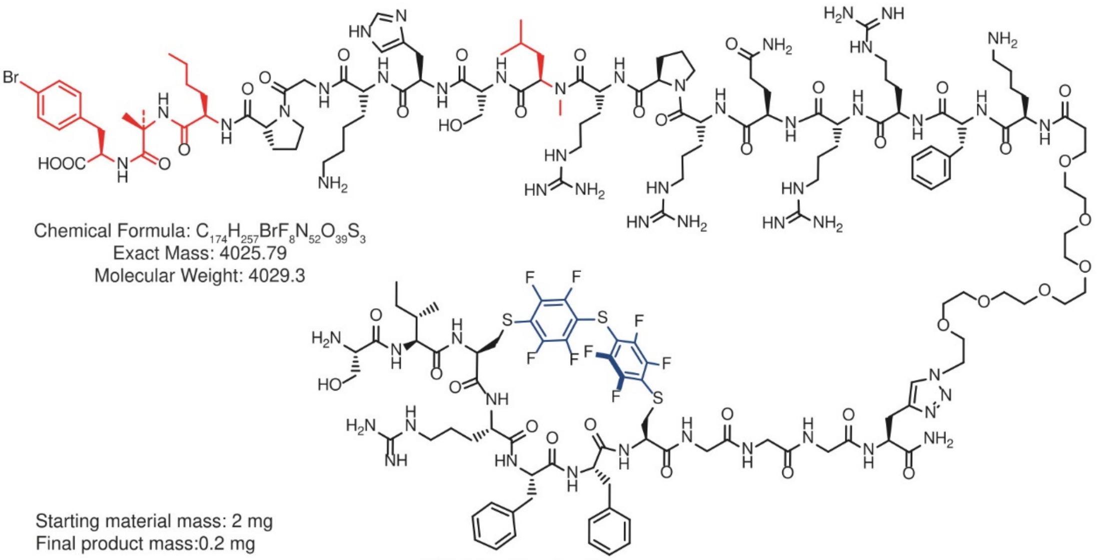

Chemical Formula: $C_{174}H_{257}BrF_8N_{52}O_{39}S_3$
Exact Mass: 4025.79
Molecular Weight: 4029.3

Starting material mass: 2 mg
Final product mass:0.2 mg

HPLC Purification Trace

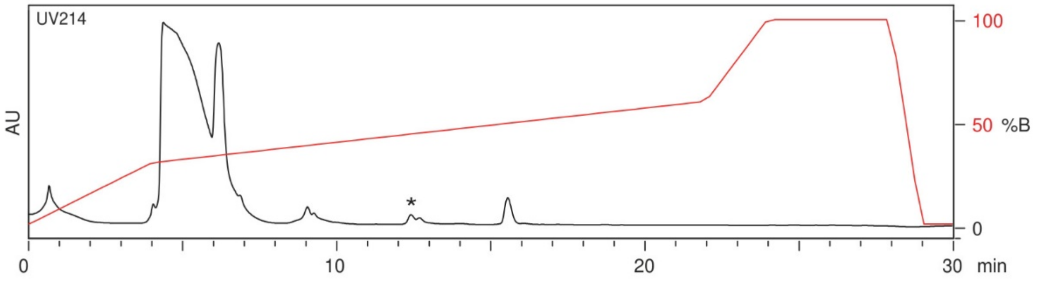

Time (min)	Solvent B (%)
0	0
2	30
21	60
23	100
26	100
29	0
30	0

Flow Rate: 13 mL / min
Phenomenex Kinetex EVO C18 Prep Column
(100 Å, 5 µm, 21.5 mm X 250 mm)

Solvent A: H_2O + 0.1% (v/v) TFA
Solvent B: MeCN + 0.1% (v/v) TFA

LCMS Trace

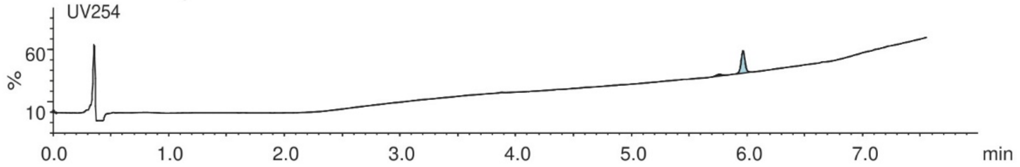

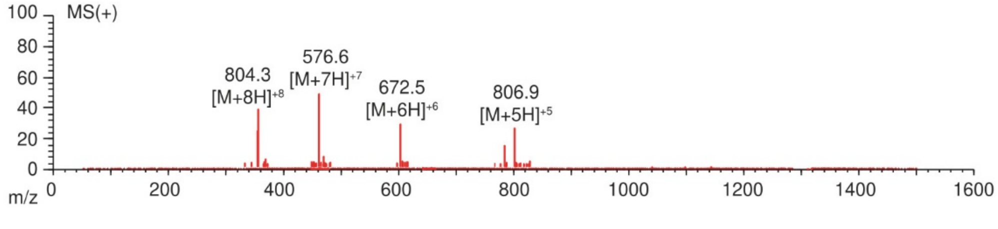

Supplementary Fig. 68. Synthesis summary of **PFS**-SICRFFCGGGZa (**20n**)

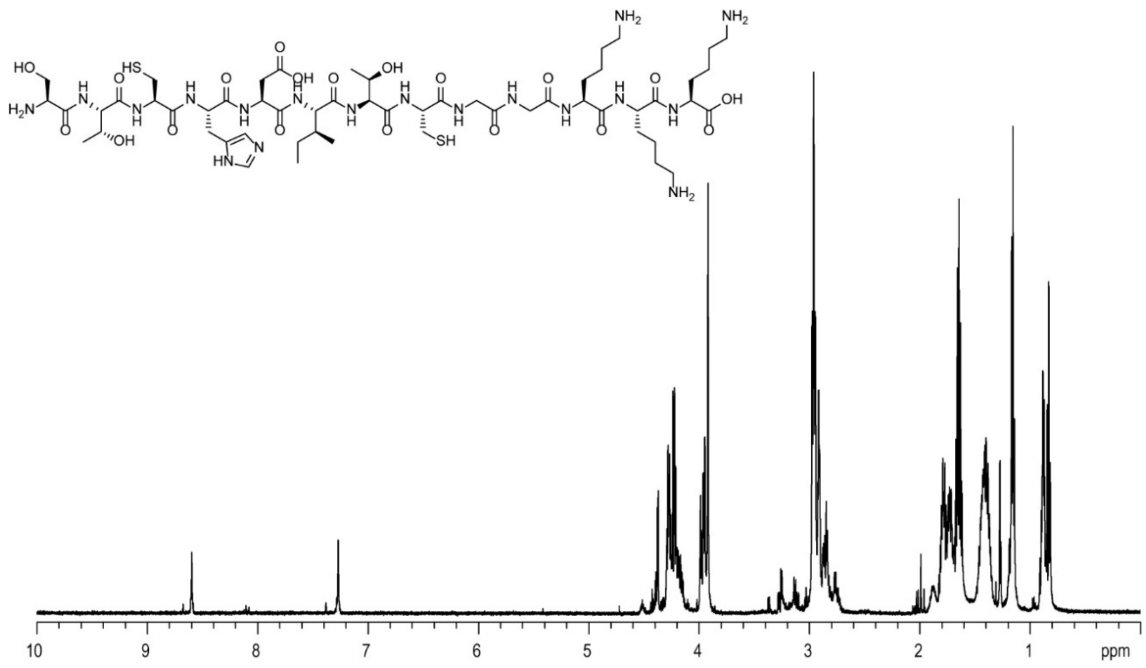

Supplementary Fig. 69. STCHDITCGGKKK (**9a**) ^{1}H NMR Spectra in D_2O, 600

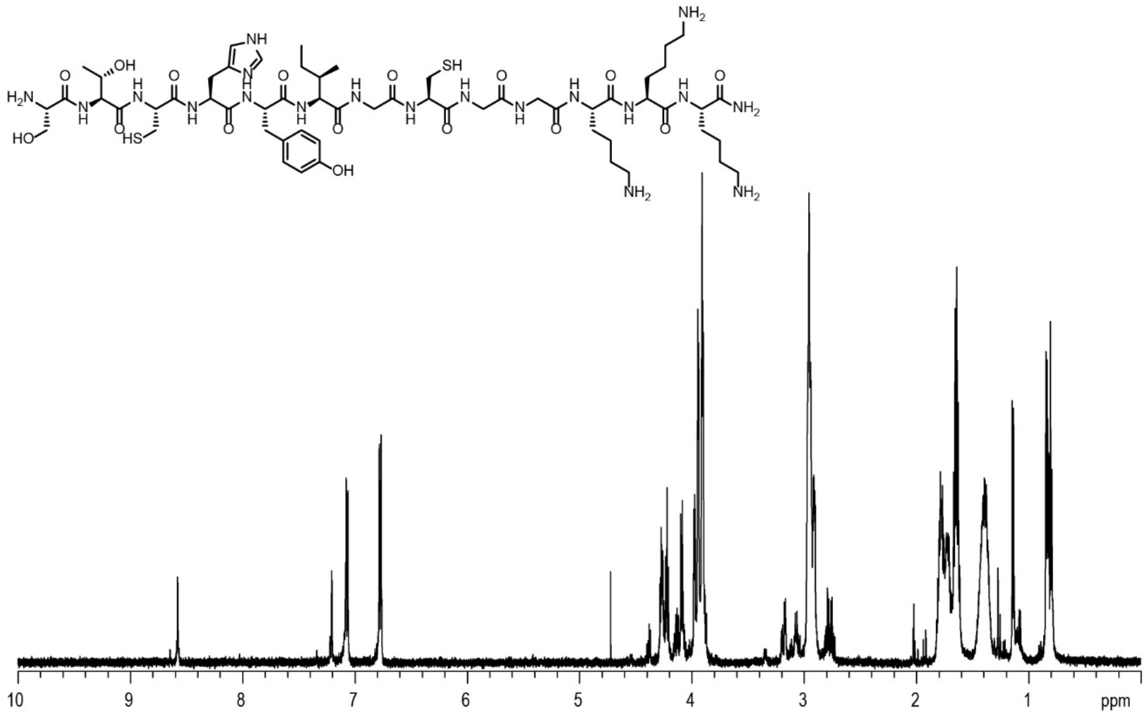

Supplementary Fig. 70. STCHYIGCGGKKK (**10a**) ^{1}H NMR Spectra in D_2O, 600

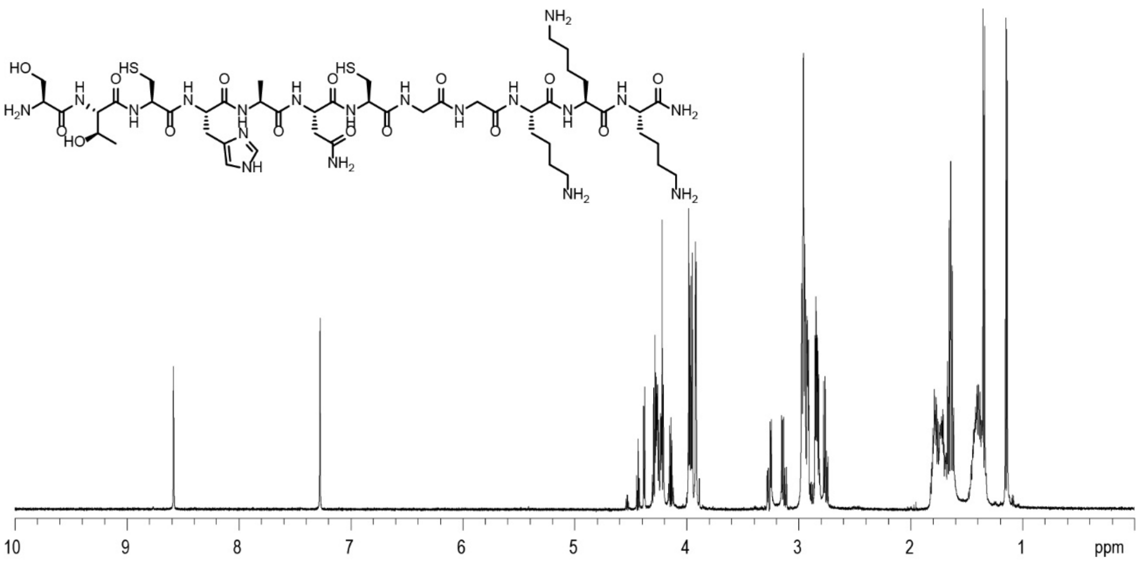

Supplementary Fig. 71. STCHANCGGKKK (**12a**) ^{1}H NMR Spectra in D_2O, 600

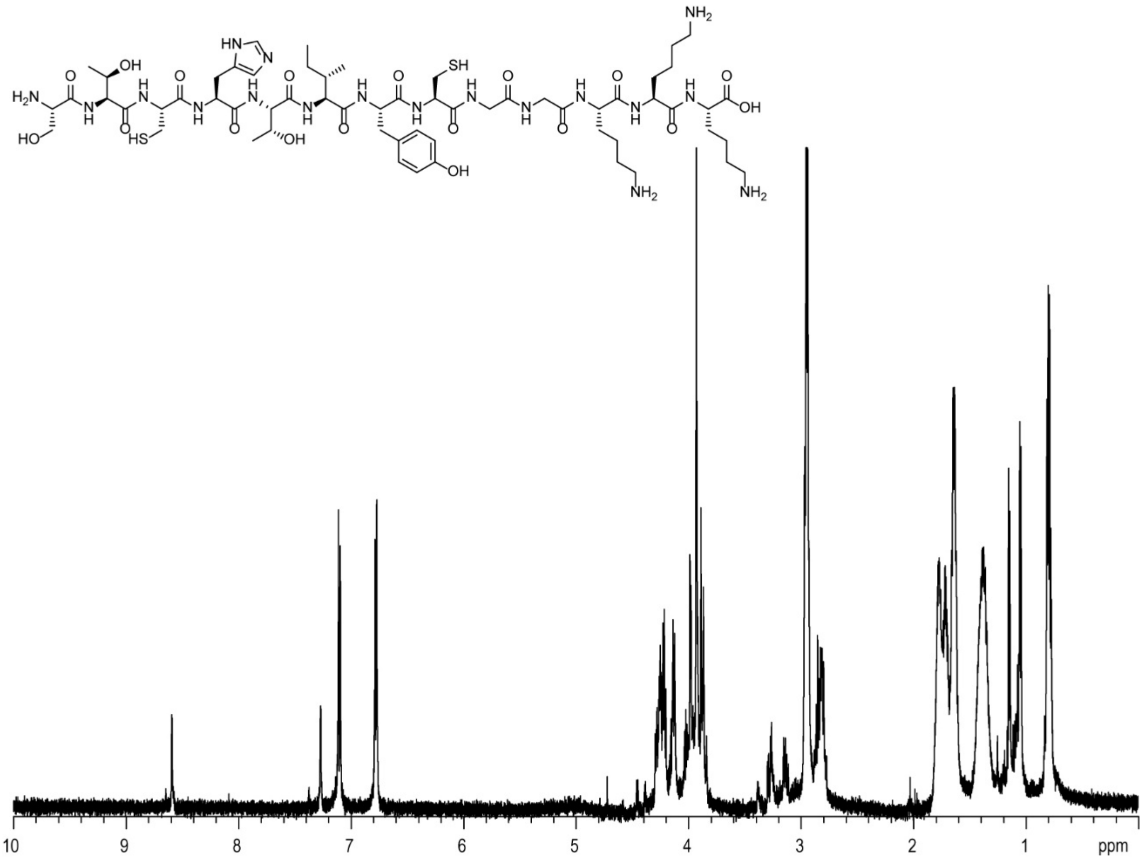

Supplementary Fig. 72. STCHTIYCGGKKK (**13a**) ^{1}H NMR Spectra in D_2O, 600

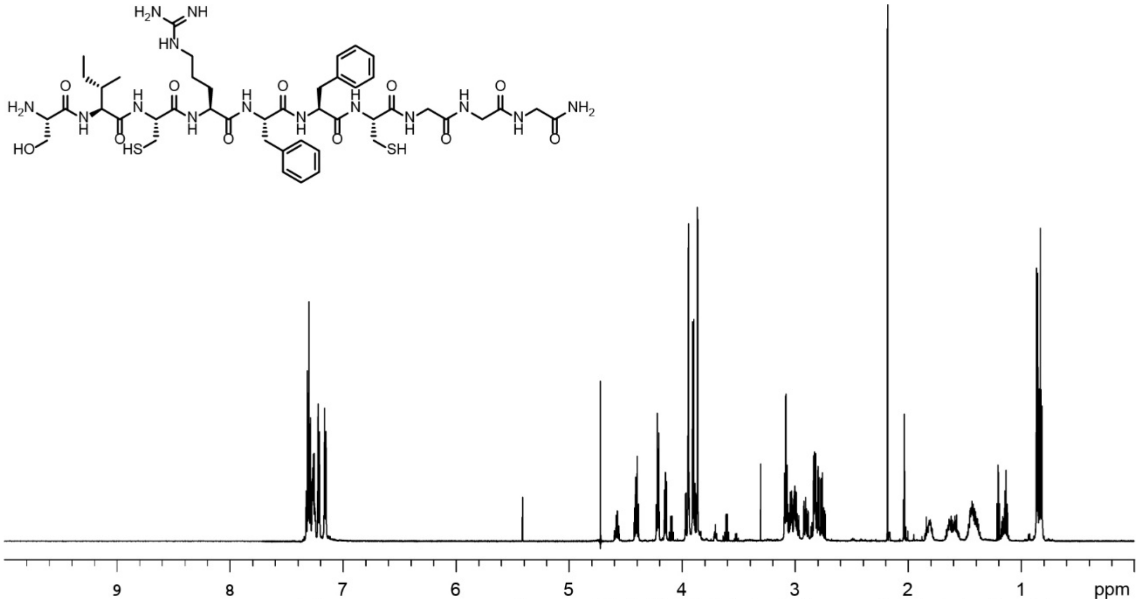

Supplementary Fig. 73. SICRFFCGGG (**14a**) ^{1}H NMR Spectra in D_2O, 600

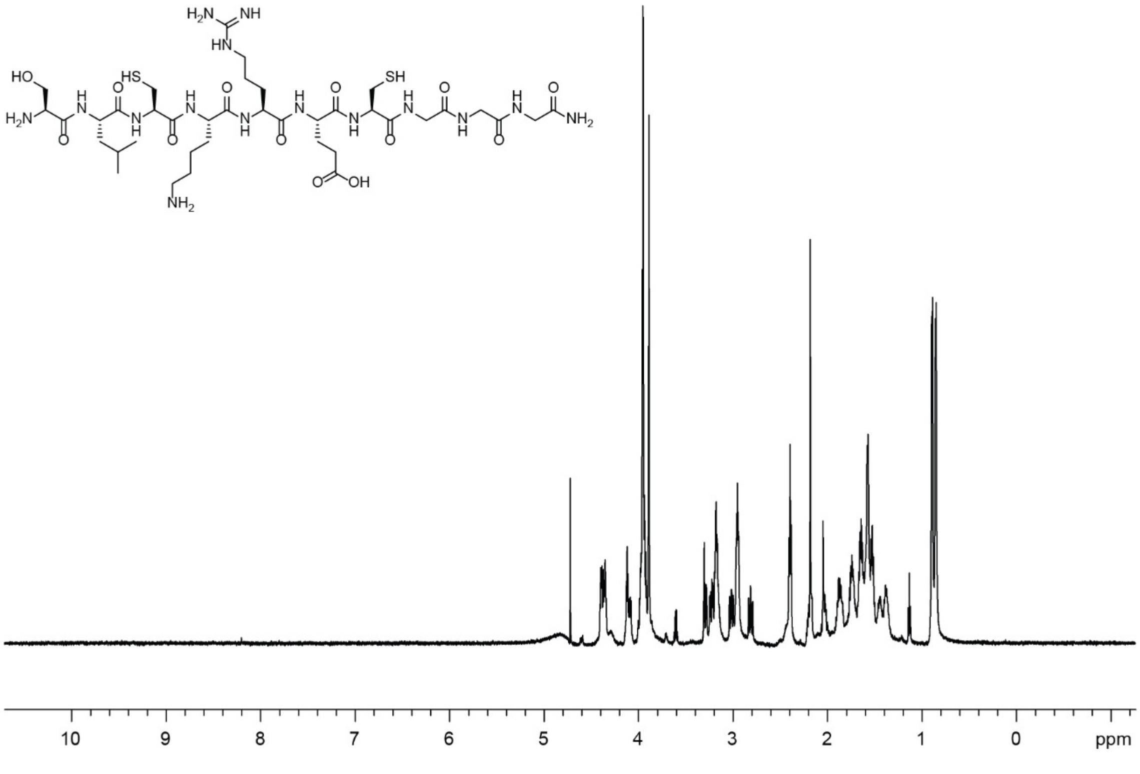

Supplementary Fig. 74. SLCKRECGGG (**16a**) ^{1}H NMR Spectra in D_2O, 600

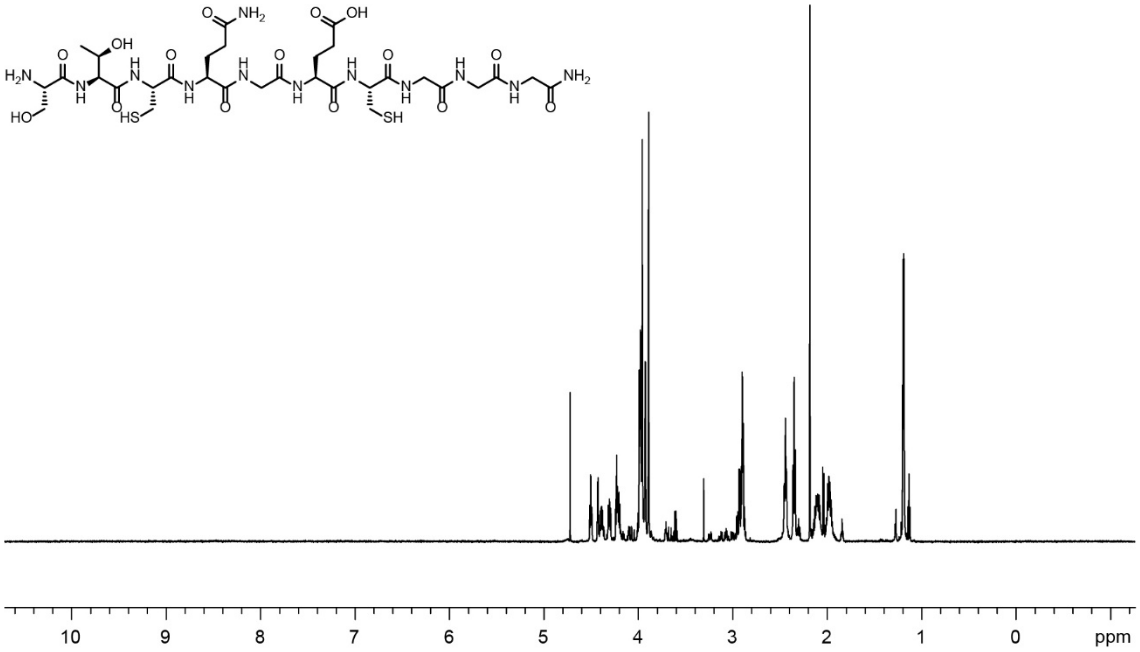

Supplementary Fig. 75. STCQGECGGG (**17a**) ^{1}H NMR Spectra in D_2O, 600

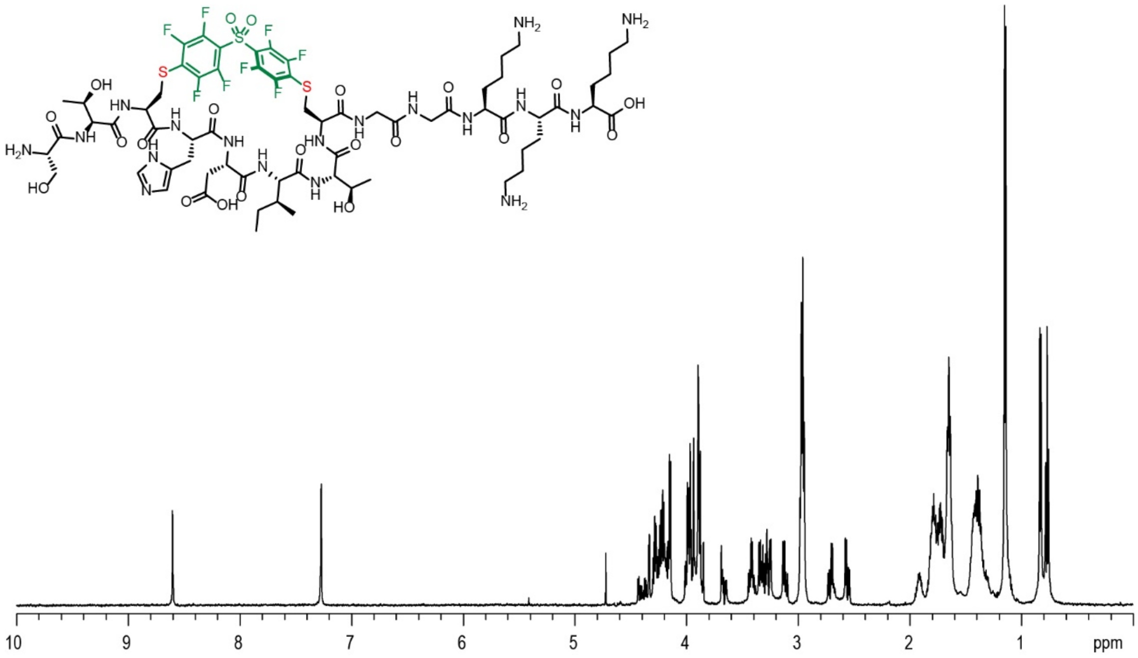

Supplementary Fig. 76. DFS-STCHDITCGGKKK (**9b**) ^{1}H NMR Spectra in D_2O, 600

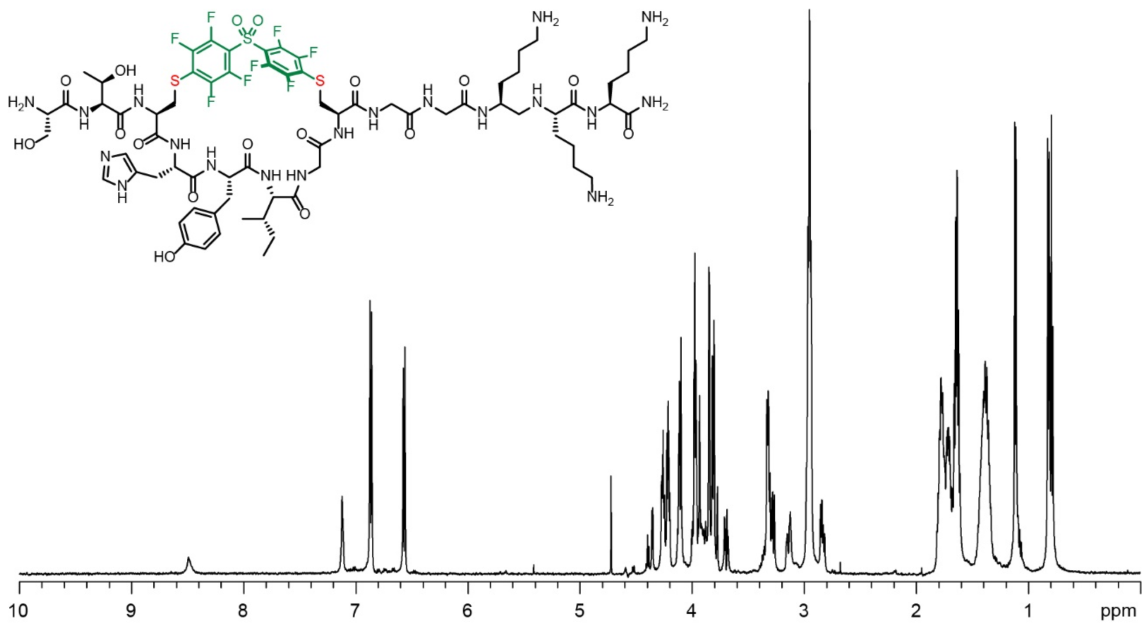

Supplementary Fig. 77. DFS-STCHYIGCGGKKK (**10b**) ^{1}H NMR Spectra in D_2O, 600

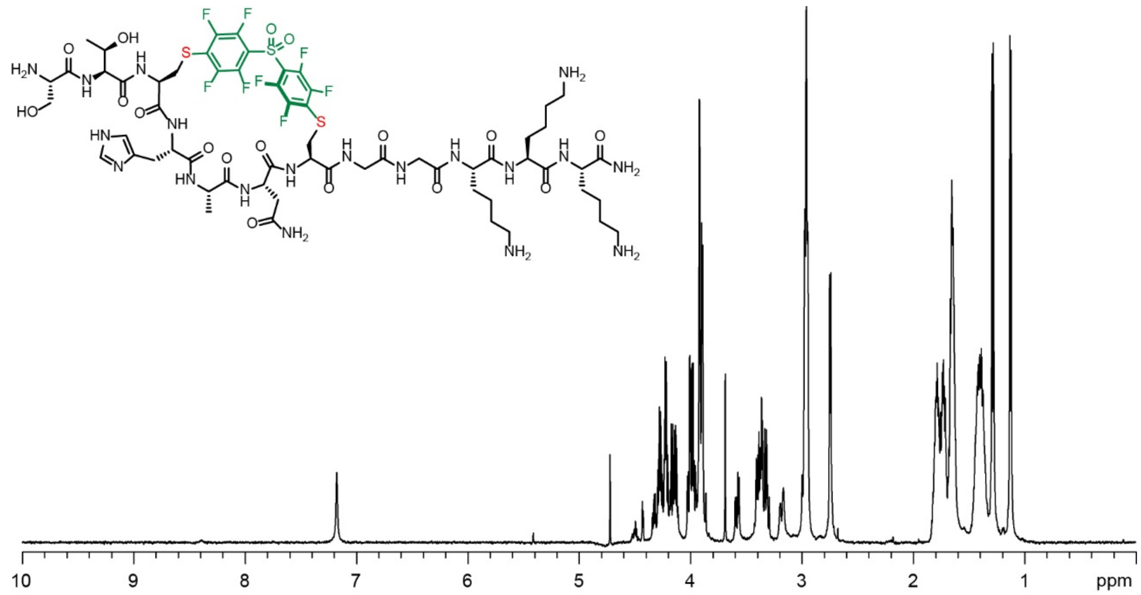

Supplementary Fig. 78. DFS-STCHANCGGKKK (**12b**) ^{1}H NMR Spectra in D_2O, 600

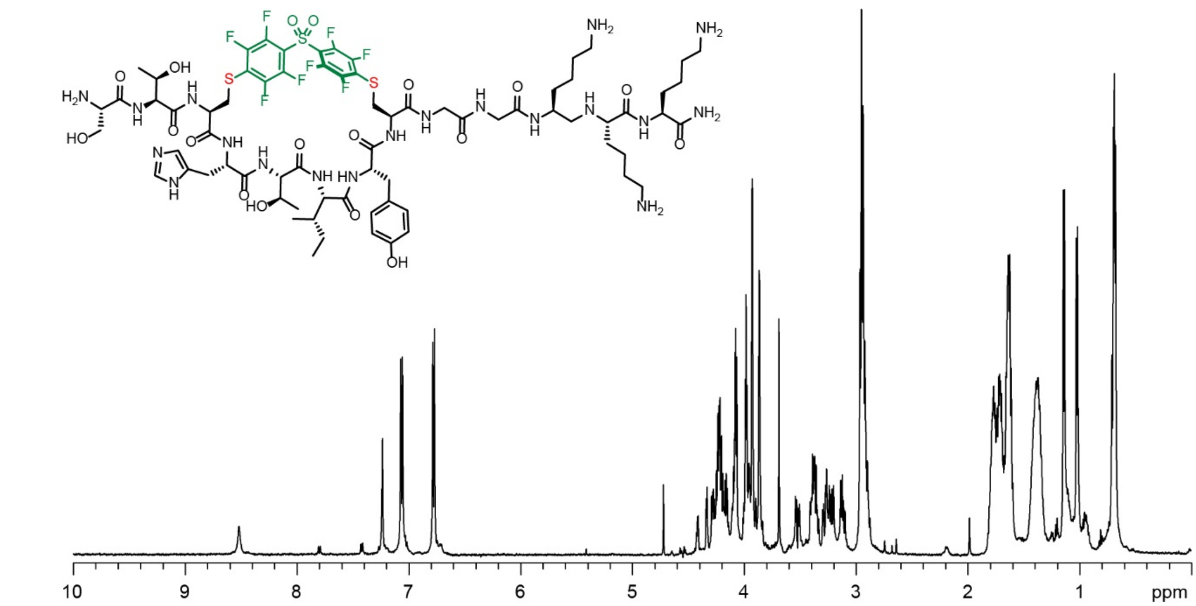

Supplementary Fig. 79. DFS-STCHTIYCGGKKK (**13b**)^{1}H NMR Spectra in D_2O, 600

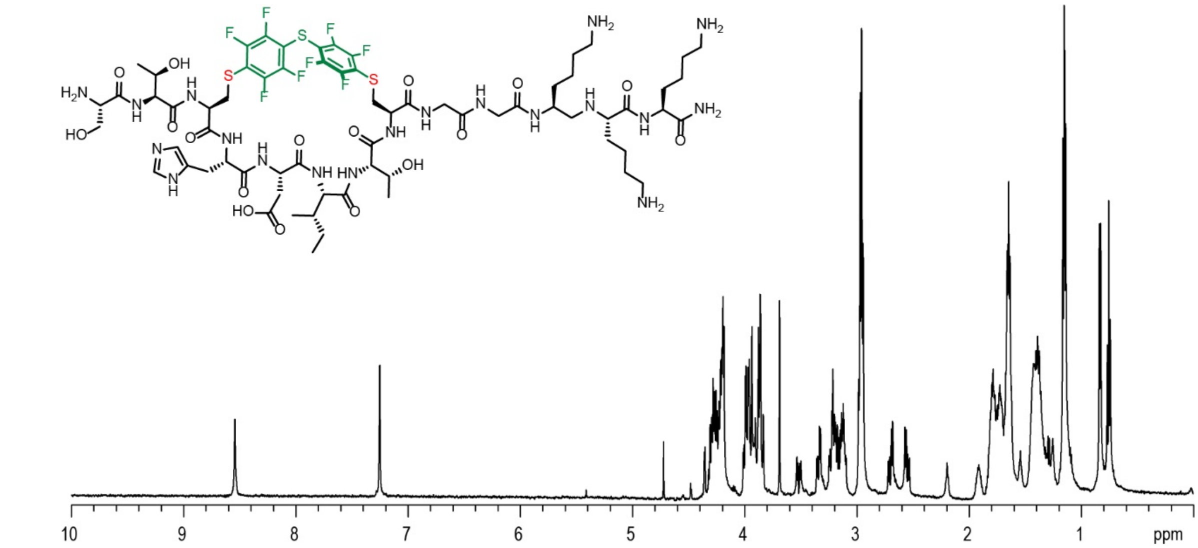

Supplementary Fig. 80. PFS-STCHDITCGGKKK (**9c**) ^{1}H NMR Spectra in D_2O, 600

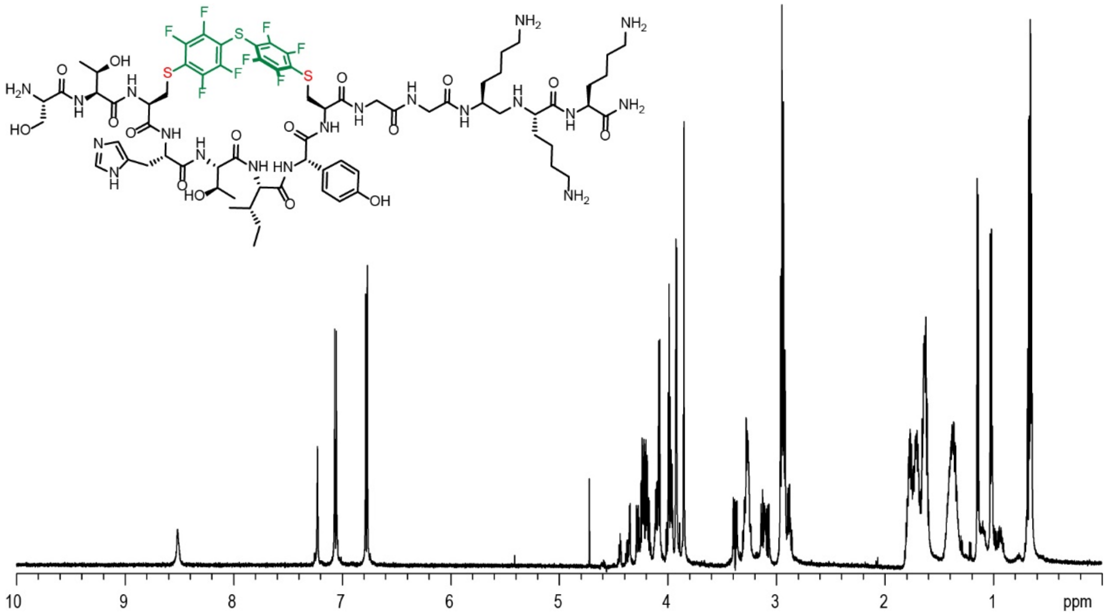

Supplementary Fig. 81. PFS-STCHTIYCGGKKK (**10c**) 1H NMR Spectra in D2O, 600

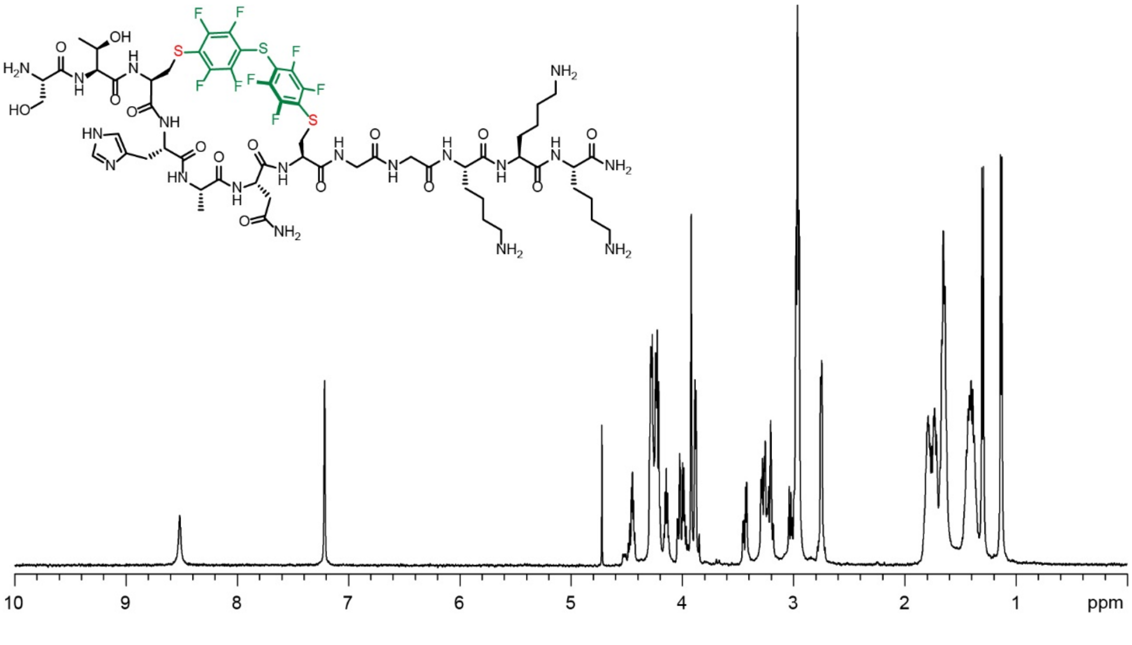

Supplementary Fig. 82. PFS-STCHANCGGKKK (**12c**) ^{1}H NMR Spectra in D_2O, 600

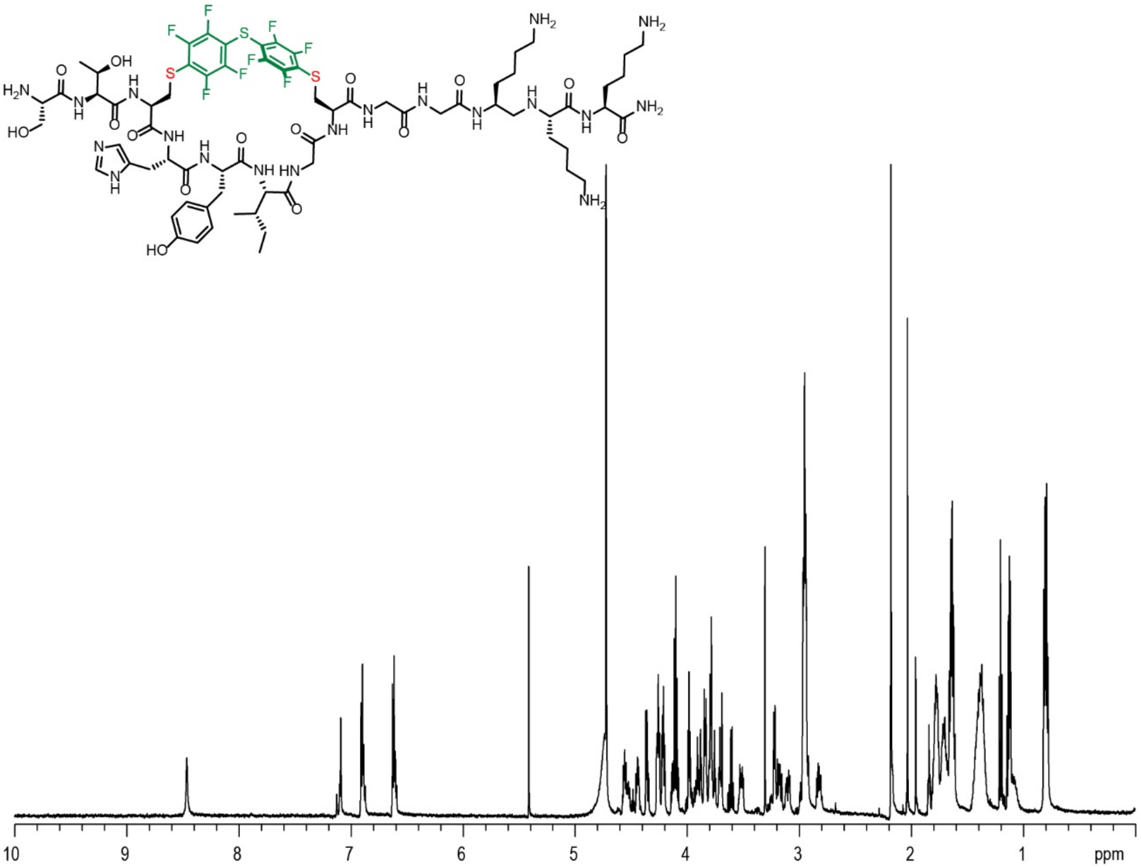

Supplementary Fig. 83. PFS-STCHYIGCGGKKK (**13c**) ^{1}H NMR Spectra in D_2O, 600

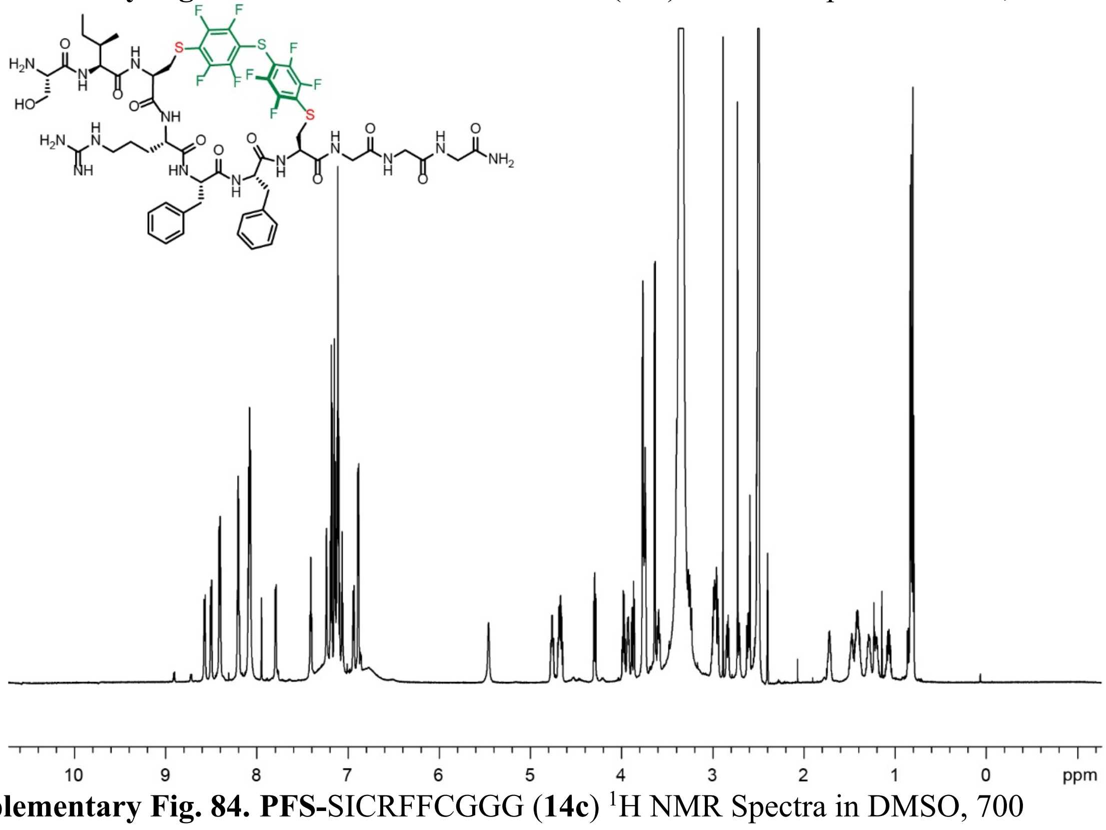

Supplementary Fig. 84. PFS-SICRFFCGGG (**14c**) ^{1}H NMR Spectra in DMSO, 700

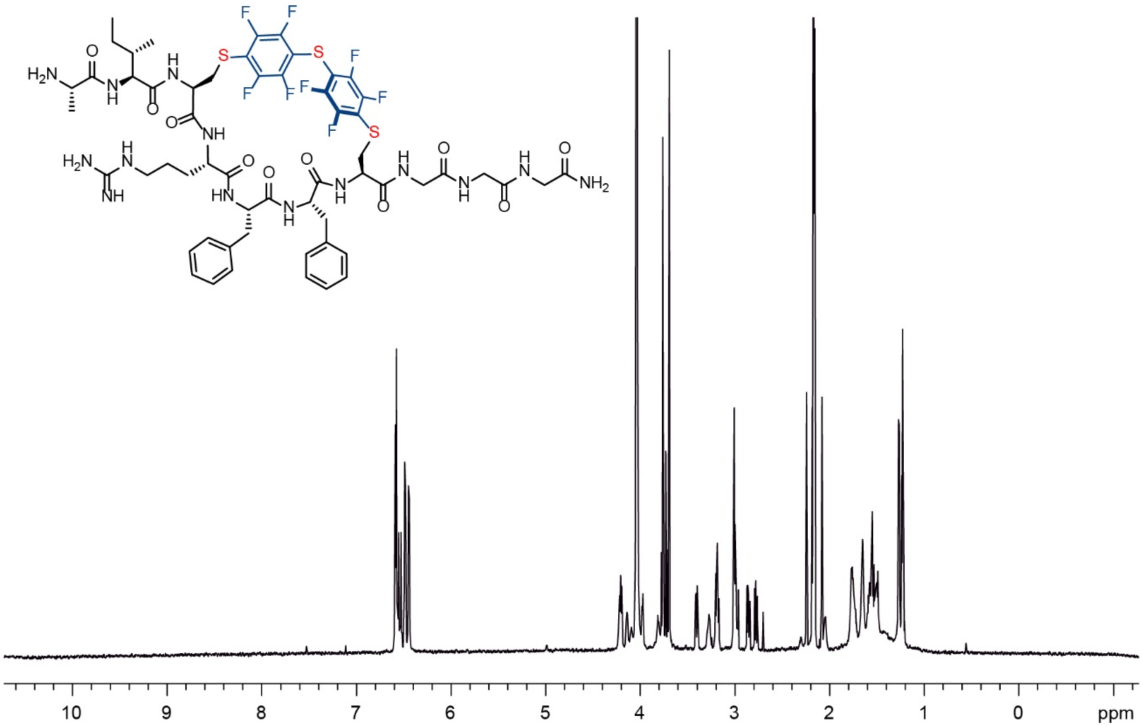

Supplementary Fig. 85. PFS-SICRFFCGGG (**14c**) ^{1}H NMR Spectra in CD_3CN:D_2O, 700

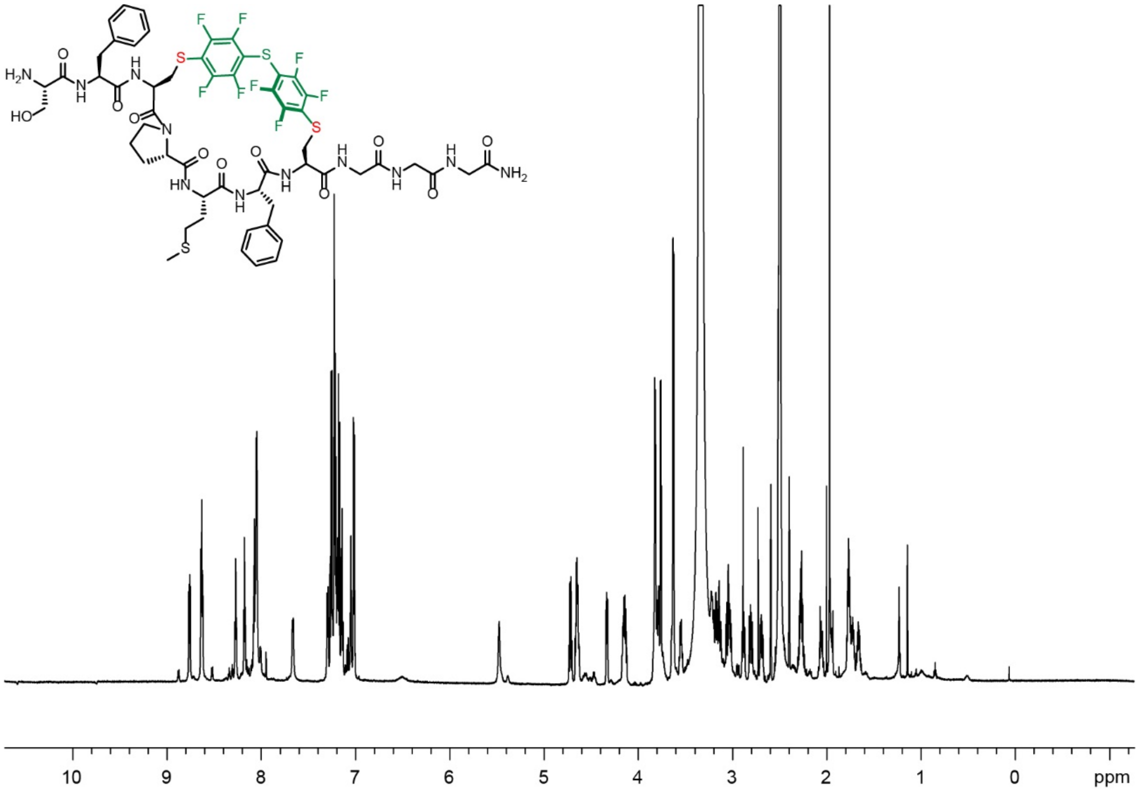

Supplementary Fig. 86. PFS-SFCPMFCGGG (**15c**) ^{1}H NMR Spectra in DMSO, 700

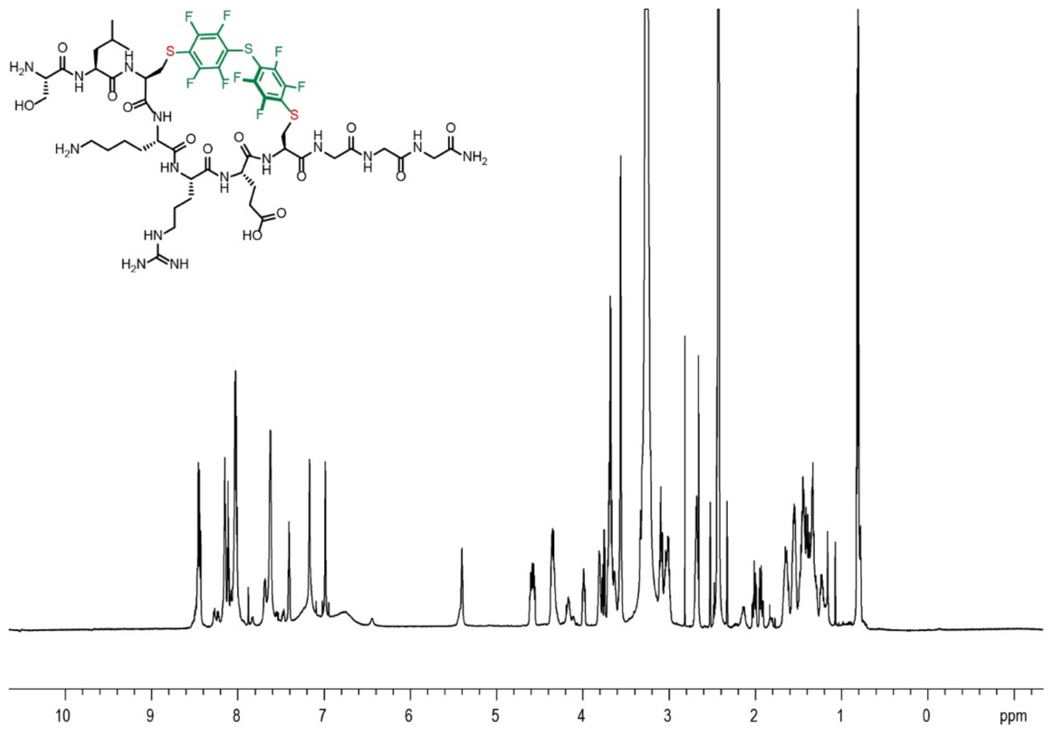

Supplementary Fig. 87. PFS-SLCKRECGGG (**16c**) 1H NMR Spectra in DMSO, 700

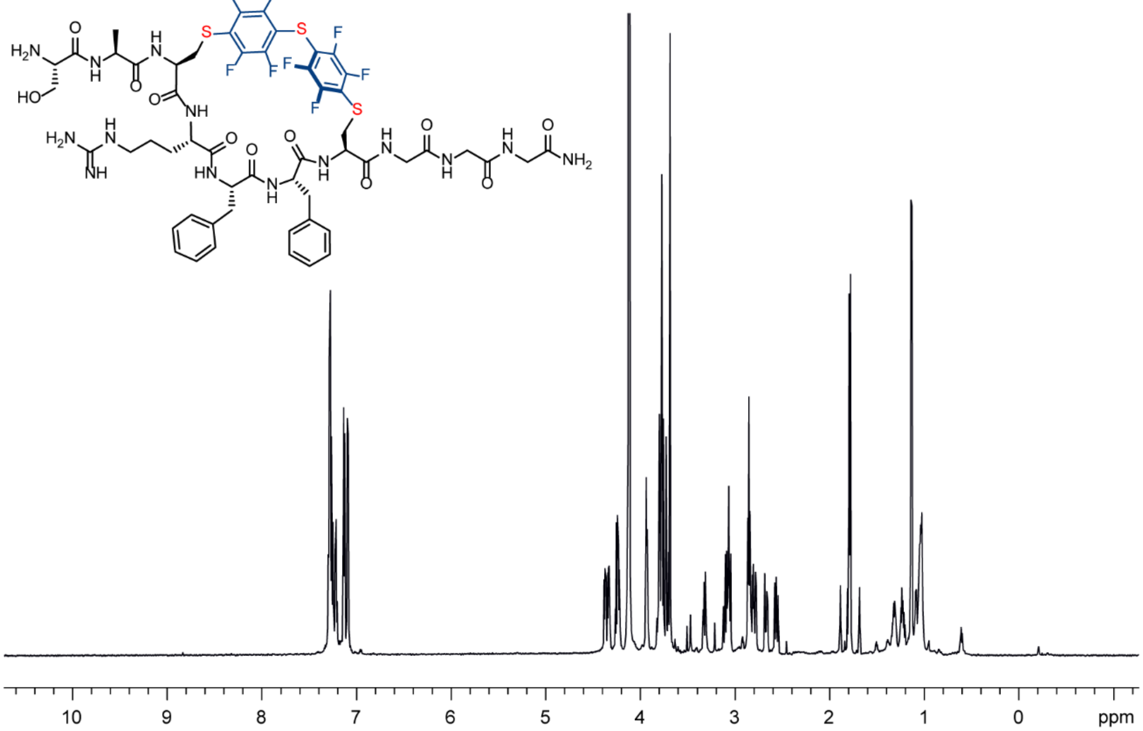

Supplementary Fig. 88. PFS-SACRFFCGGG (**21c**) ^{1}H NMR Spectra in CD_3CN:D_2O, 700

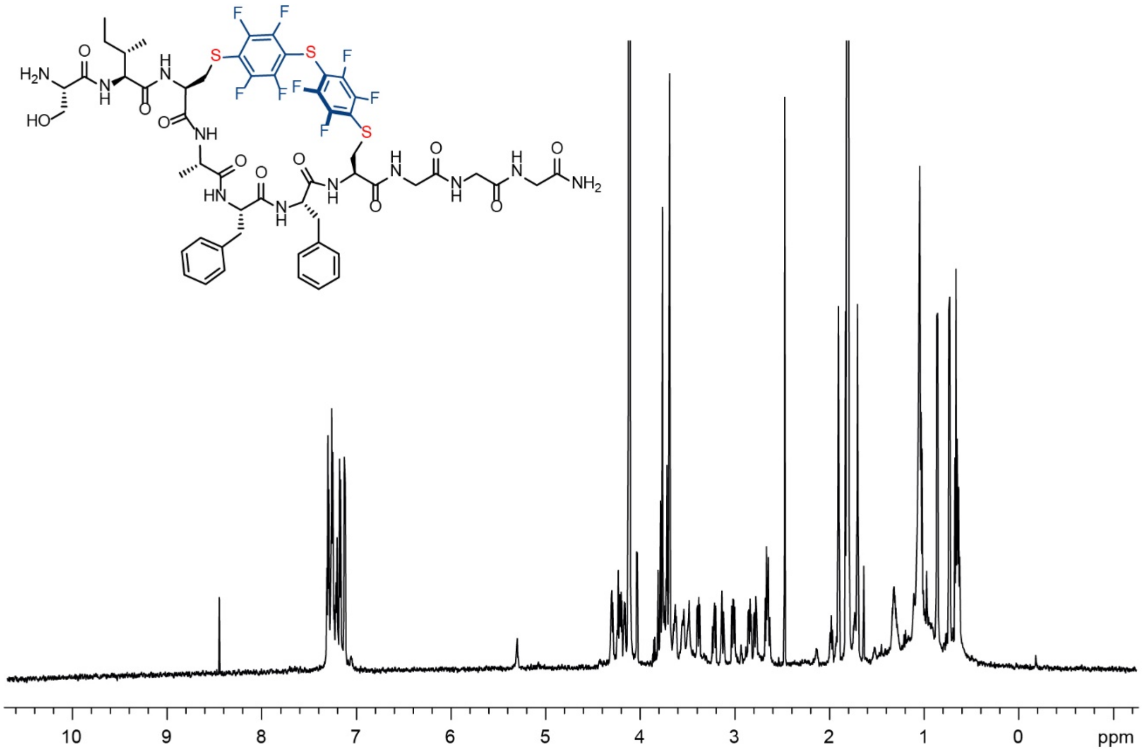

Supplementary Fig. 89. PFS-SICAFFCGGG (**22c**) ^{1}H NMR Spectra in CD_3CN:D_2O, 700

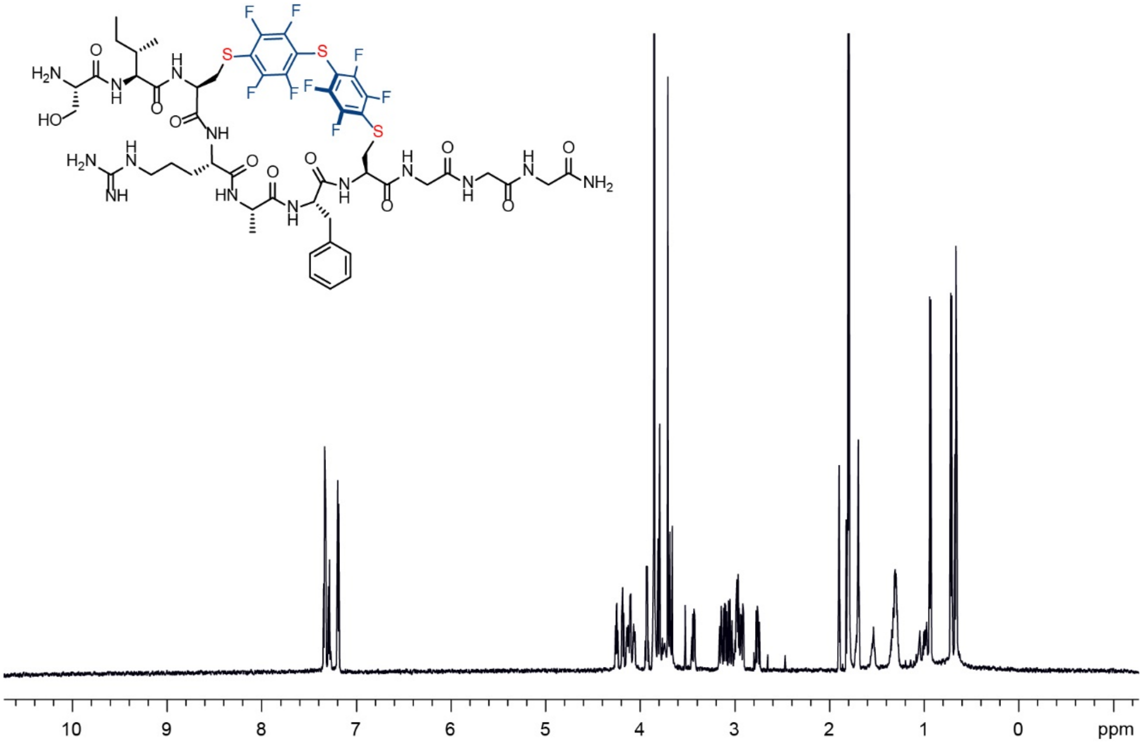

Supplementary Fig. 90. PFS-SICRAFCGGG (**23c**) ^{1}H NMR Spectra in CD_3CN:D_2O, 700

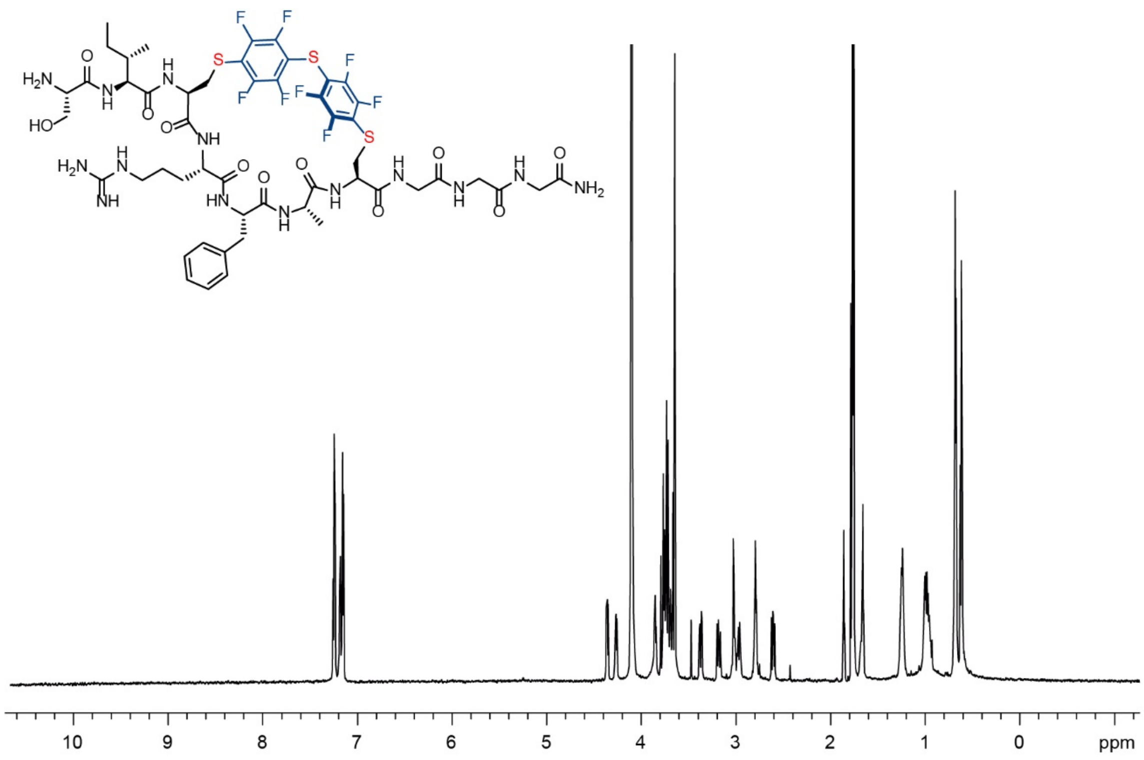

Supplementary Fig. 91. PFS-SICRFACGGG (**24c**) ^{1}H NMR Spectra in $CD_3CN:D_2O$, 700

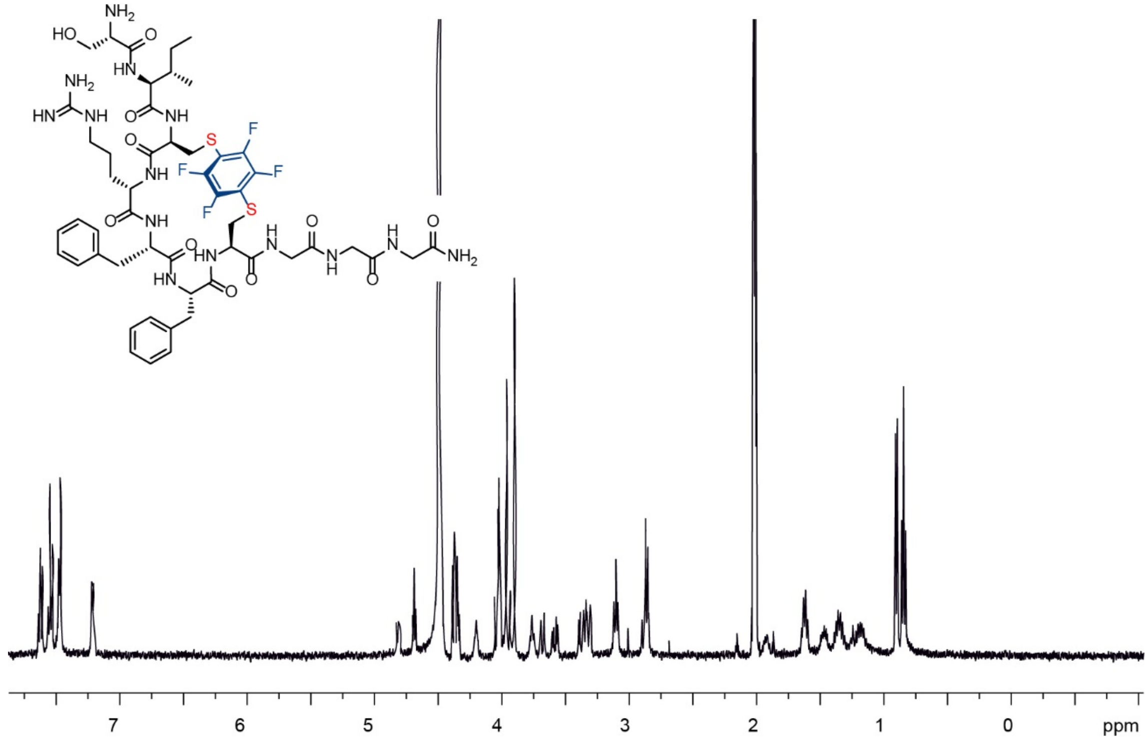

Supplementary Fig. 92. HFB-SICRFFCGGG (**12j**) ^{1}H NMR Spectra in DMSO, 700

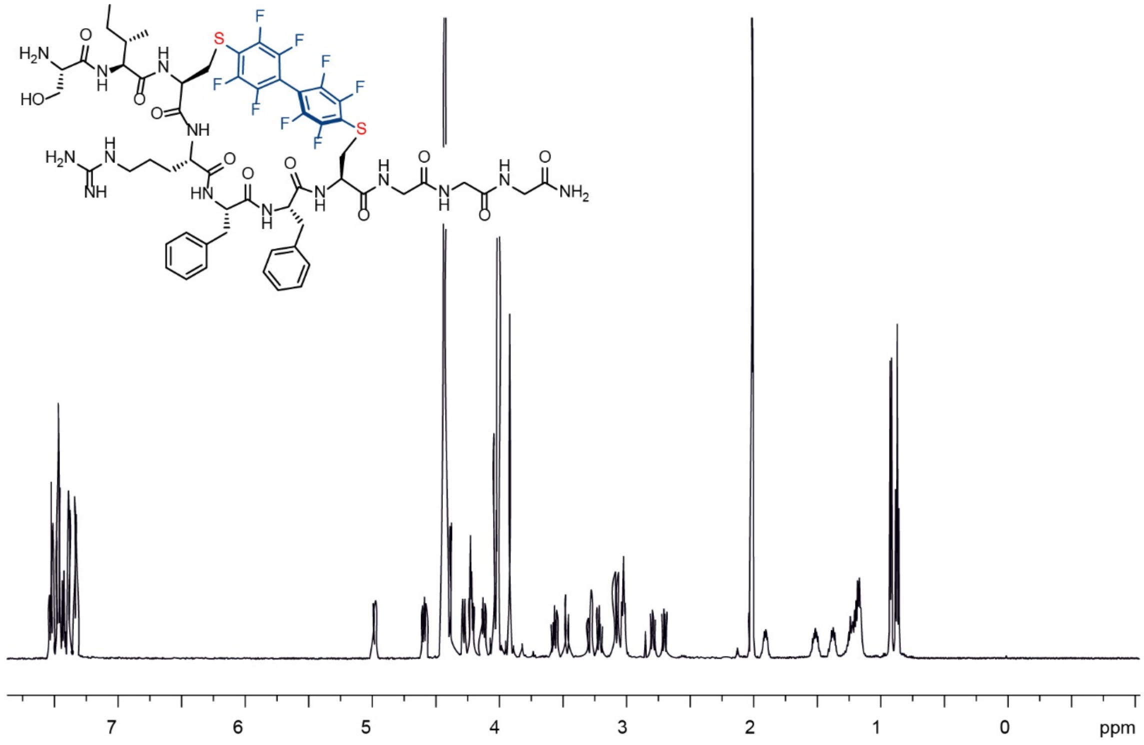

Supplementary Fig. 93. DFB-SICRFFCGGG (**12k**) 1H NMR Spectra in $CD_3CN:D_2O$, 700

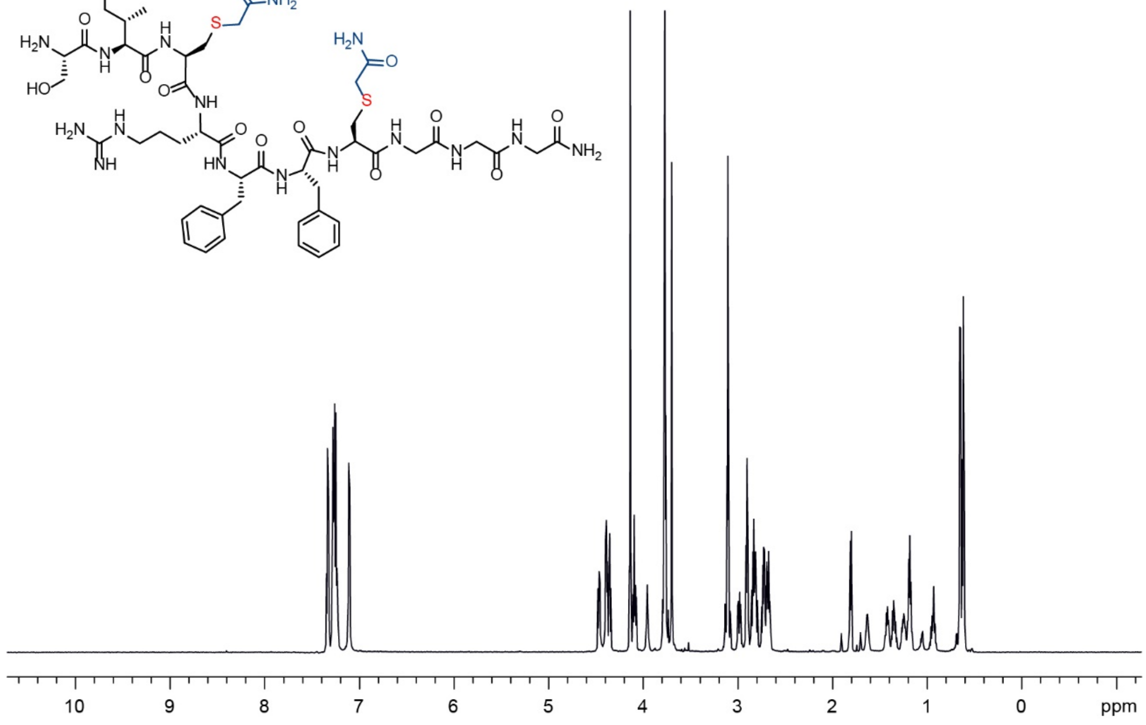

Supplementary Fig. 94. IA-SICRFFCGGG (**14l**) ^1H NMR Spectra in $CD_3CN:D_2O$, 700

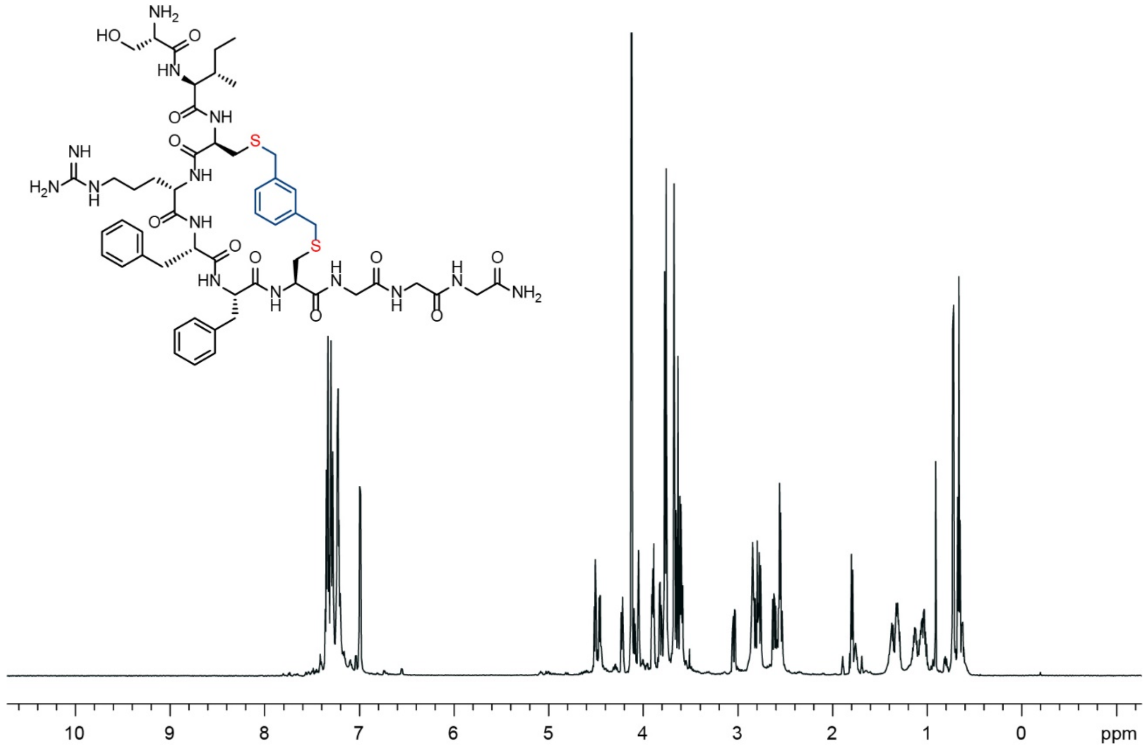

Supplementary Fig. 95. MBX-SICRFFCGGG (**14m**) ^{1}H NMR Spectra in CD_3CN:D_2O, 700

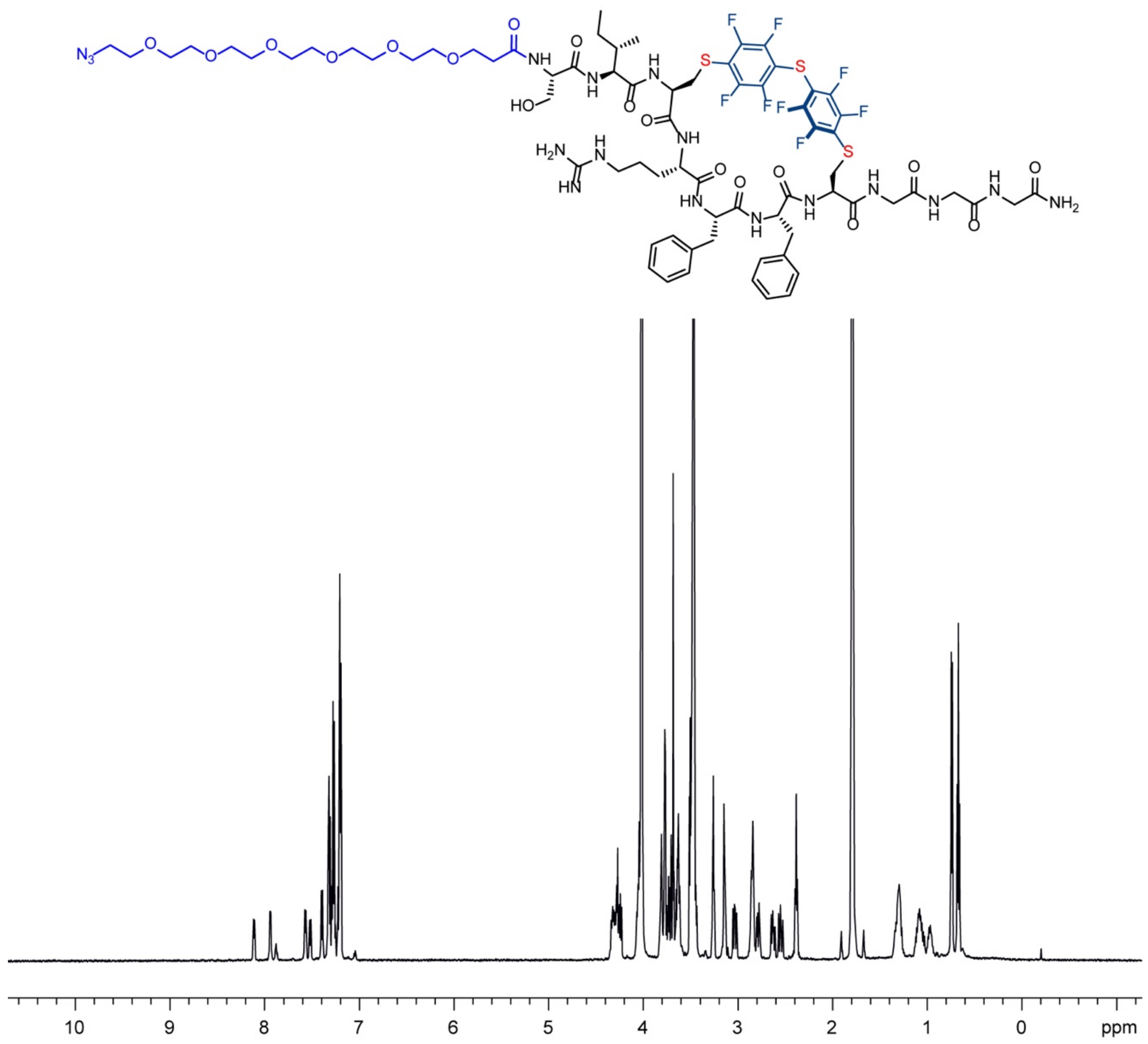

Supplementary Fig. 96. Np7-**PFS**-SICRFFCGGG (**14g**) ^{1}H NMR Spectra in CD_3CN:D_2O, 700

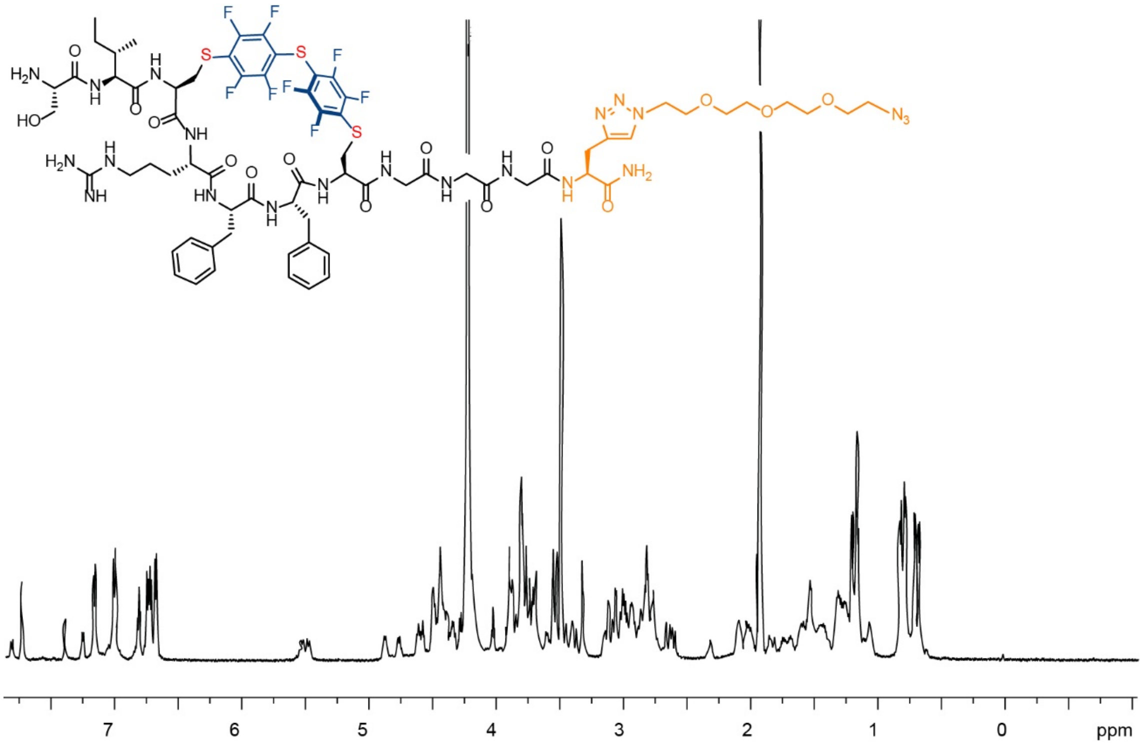

Supplementary Fig. 97. PFS-SICRFFCGGGZp4 (**20h**) 1H NMR Spectra in $CD_3CN:D_2O$, 700

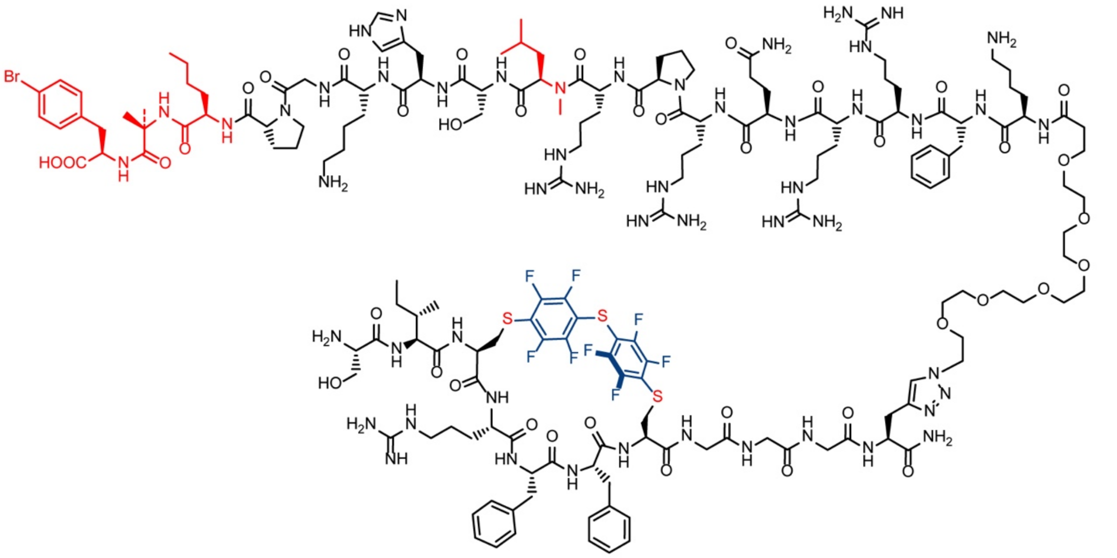

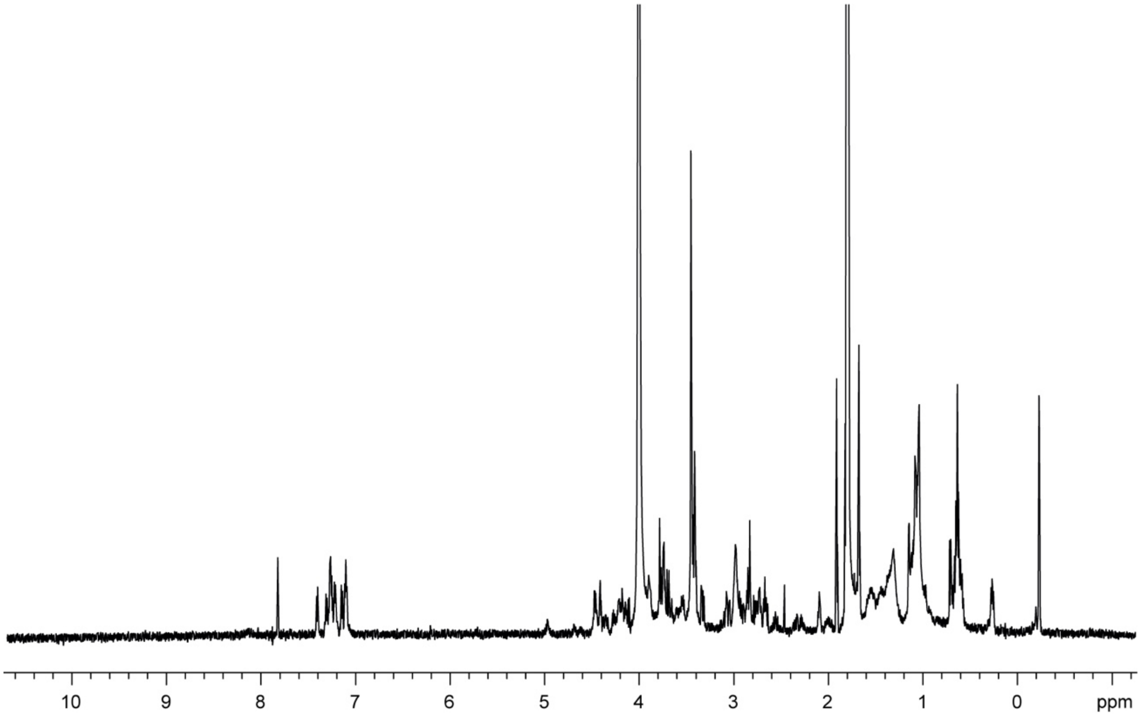

Supplementary Fig. 98. PFS-SICRFFCGGGZa (**20n**) ^1H NMR Spectra in $CD_3CN:D_2O$, 700

Supplementary References

1 He, B. *et al.* Compositional Bias in Naive and Chemically-modified Phage-Displayed

Libraries uncovered by Paired-end Deep Sequencing. *Sci. Rep.* **8**, 1214, doi:10.1038/s41598-018-19439-2 (2018).

2 Sojitra, M. *et al.* Genetically encoded multivalent liquid glycan array displayed on M13 bacteriophage. *Nat. Chem. Biol.* **17**, 806-816, doi:10.1038/s41589-021-00788-5 (2021).

3 Tjhung, K. F. *et al.* Silent Encoding of Chemical Post-Translational Modifications in Phage-Displayed Libraries. *J Am Chem Soc* **138**, 32-35, doi:10.1021/jacs.5b10390 (2016).

4 Kalhor-Monfared, S. *et al.* Rapid biocompatible macrocyclization of peptides with decafluoro-diphenylsulfone. *Chem. Sci.* **7**, 3785-3790, doi:10.1039/c5sc03856a (2016).

5 Spokoyny, A. M. *et al.* A perfluoroaryl-cysteine S(N)Ar chemistry approach to unprotected peptide stapling. *J. Am. Chem. So.c* **135**, 5946-5949, doi:10.1021/ja400119t (2013).

6 Bhattacharya, A. A., Grune, T. & Curry, S. Crystallographic analysis reveals common modes of binding of medium and long-chain fatty acids to human serum albumin. *J. Mol. Biol.* **303**, 721-732, doi:10.1006/jmbi.2000.4158 (2000).

7 Trott, O. & Olson, A. J. AutoDock Vina: improving the speed and accuracy of docking with a new scoring function, efficient optimization, and multithreading. *J. Comput. Chem.* **31**, 455-461, doi:10.1002/jcc.21334 (2010).

8 Wang, Z. M. *et al.* Structural studies of several clinically important oncology drugs in complex with human serum albumin. *Biochim. Biophys. Acta.* **1830**, 5356-5374, doi:10.1016/j.bbagen.2013.06.032 (2013).

9 Zsila, F. Subdomain IB is the third major drug binding region of human serum albumin: toward the three-sites model. *Mol. Pharm.* **10**, 1668-1682, doi:10.1021/mp400027q (2013).

10 Zunszain, P. A., Ghuman, J., Komatsu, T., Tsuchida, E. & Curry, S. Crystal structural analysis of human serum albumin complexed with hemin and fatty acid. *BMC Struct. Biol.*

3, 6, doi:10.1186/1472-6807-3-6 (2003).

11 Essmann, U. *et al.* A smooth particle mesh Ewald method. *J. Chem. Phys.* **103**, 8577-8593, doi:10.1063/1.470117 (1995).

12 Torrie, G. M. & Valleau, J. P. Nonphysical sampling distributions in Monte Carlo free-energy estimation: Umbrella sampling. *J. Comput. Phys.* **23**, 187-199, doi:10.1016/0021-9991(77)90121-8 (1977).

13 Kumar, S., Rosenberg, J. M., Bouzida, D., Swendsen, R. H. & Kollman, P. A. THE weighted histogram analysis method for free-energy calculations on biomolecules. I. The method. *J. Comput. Chem.* **13**, 1011-1021, doi:10.1002/jcc.540130812 (1992).

14 Berendsen, H. J. C., van der Spoel, D. & van Drunen, R. GROMACS: A message-passing parallel molecular dynamics implementation *Comput. Phys. Commun.* **91**, 43 - 56 (1995).

15 Zwanzig, R. W. High-Temperature Equation of State by a Perturbation Method. I. Nonpolar Gases. *J. Chem. Phys.* **22**, 1420-1426, doi:10.1063/1.1740409 (1954).

16 Shirts, M. R. & Mobley, D. L. in *Biomolecular Simulations: Methods and Protocols* (eds Luca Monticelli & Emppu Salonen) 271-311 (Humana Press, 2013).

17 Boresch, S., Tettinger, F., Leitgeb, M. & Karplus, M. Absolute Binding Free Energies: A Quantitative Approach for Their Calculation. *J. Phys. Chem. B* **107**, 9535-9551, doi:10.1021/jp0217839 (2003).

18 Clark, A. J. *et al.* Relative Binding Affinity Prediction of Charge-Changing Sequence Mutations with FEP in Protein-Protein Interfaces. *J. Mol. Biol.* **431**, 1481-1493, doi:10.1016/j.jmb.2019.02.003 (2019).

19 Huang, J. & MacKerell, A. D., Jr. CHARMM36 all-atom additive protein force field: validation based on comparison to NMR data. *J. Comput. Chem.* **34**, 2135-2145,

doi:10.1002/jcc.23354 (2013).
