# Supplementary material for: Genetically encoded discovery of perfluoroaryl macrocycles that bind to albumin and exhibit extended circulation in vivo
Source: Nat Commun. 2023 Sep 13;14:5654. doi: 10.1038/s41467-023-41427-y (PMC10499988; doi:10.1038/s41467-023-41427-y)
Supplement: Supplementary file 4 — Description of Additional Supplementary files [file 41467_2023_41427_MOESM4_ESM.pdf]

Description of additional Supplementary files

File name: Supplementary Data 1
Description: Contains script for sequencing analysis, and pymol structures for supplementary figures 20 and 2
