# Supplementary figures and images for: Genetically encoded discovery of perfluoroaryl macrocycles that bind to albumin and exhibit extended circulation in vivo
Source: Nat Commun. 2023 Sep 13;14:5654. doi: 10.1038/s41467-023-41427-y (PMC10499988; doi:10.1038/s41467-023-41427-y)

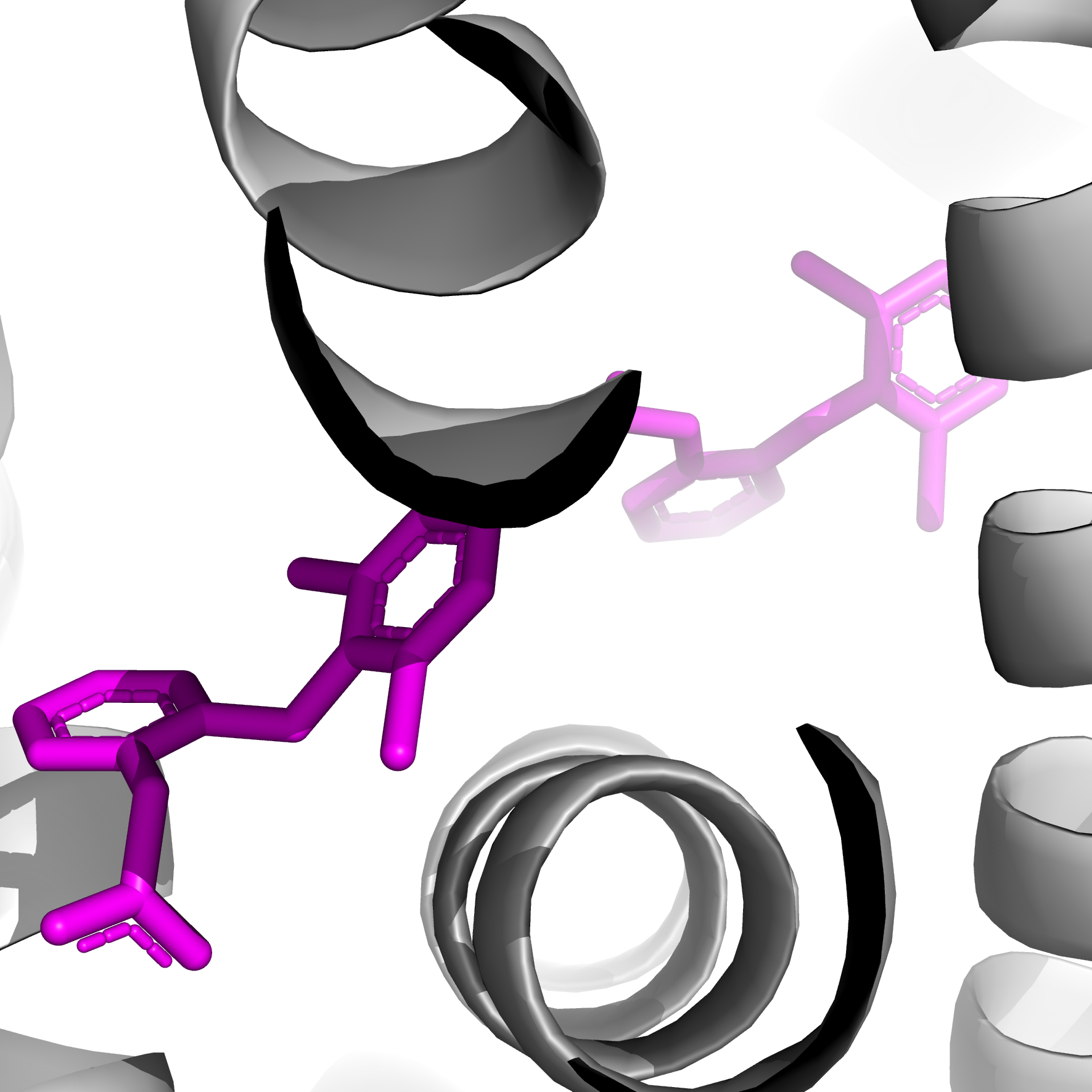

Supplement: Supplementary file 5 — Supplementary Data [file 41467_2023_41427_MOESM5_ESM.zip › Supplementary_Data/Pymol/DIF_zzom.png]

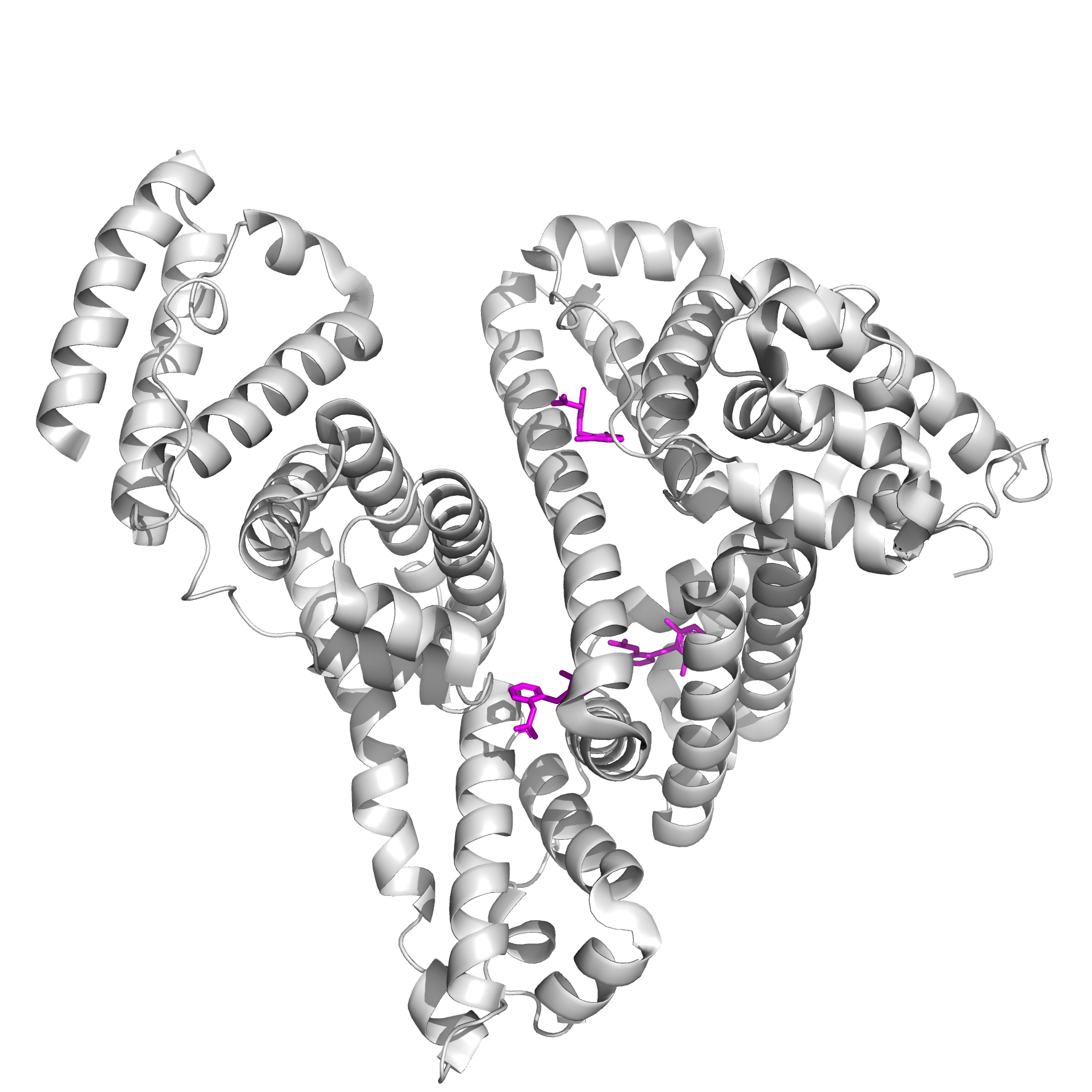

Supplement: Supplementary file 5 — Supplementary Data [file 41467_2023_41427_MOESM5_ESM.zip › Supplementary_Data/Pymol/DIF.png]

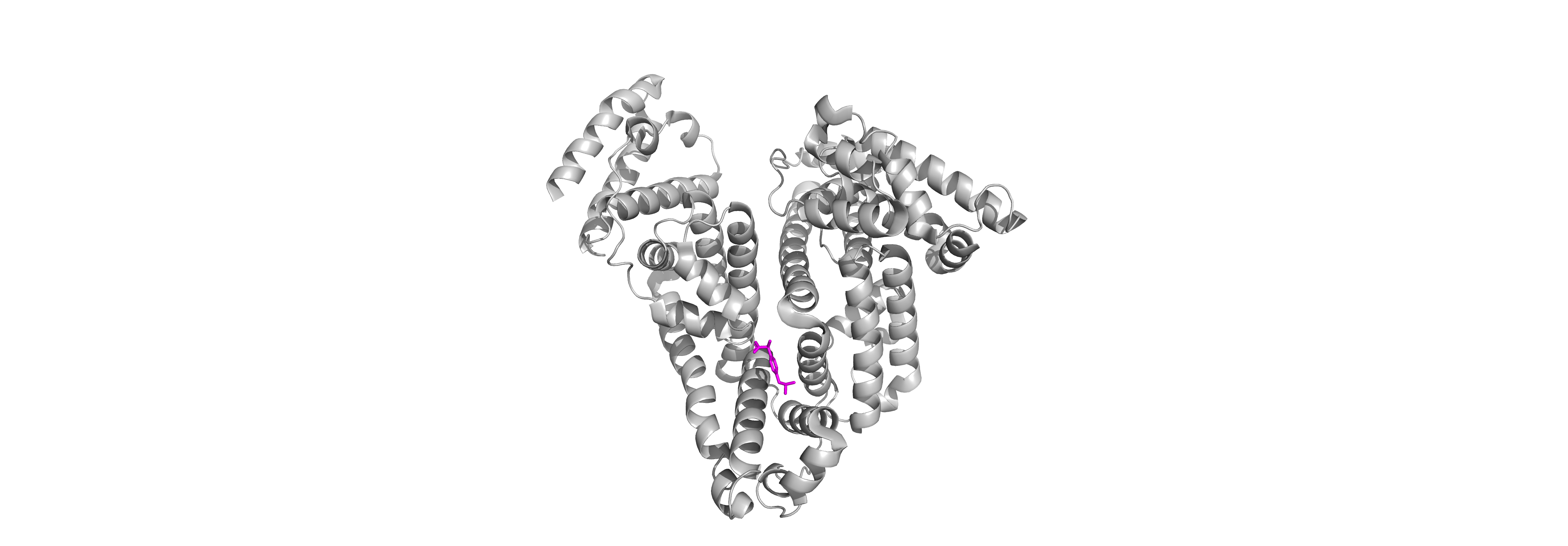

Supplement: Supplementary file 5 — Supplementary Data [file 41467_2023_41427_MOESM5_ESM.zip › Supplementary_Data/Pymol/Ibuprofen.png]

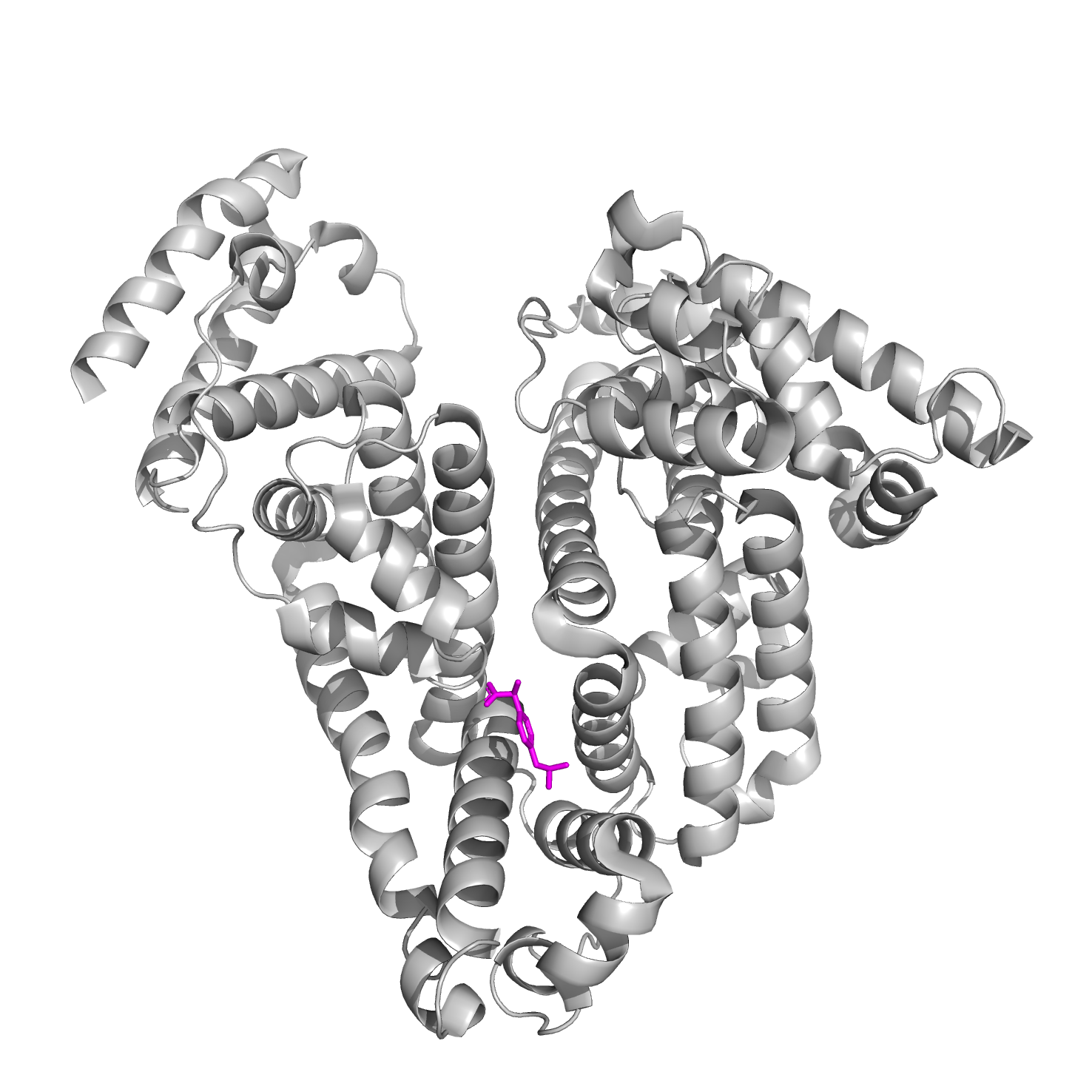

Supplement: Supplementary file 5 — Supplementary Data [file 41467_2023_41427_MOESM5_ESM.zip › Supplementary_Data/Pymol/Ibuprofen_2.png]

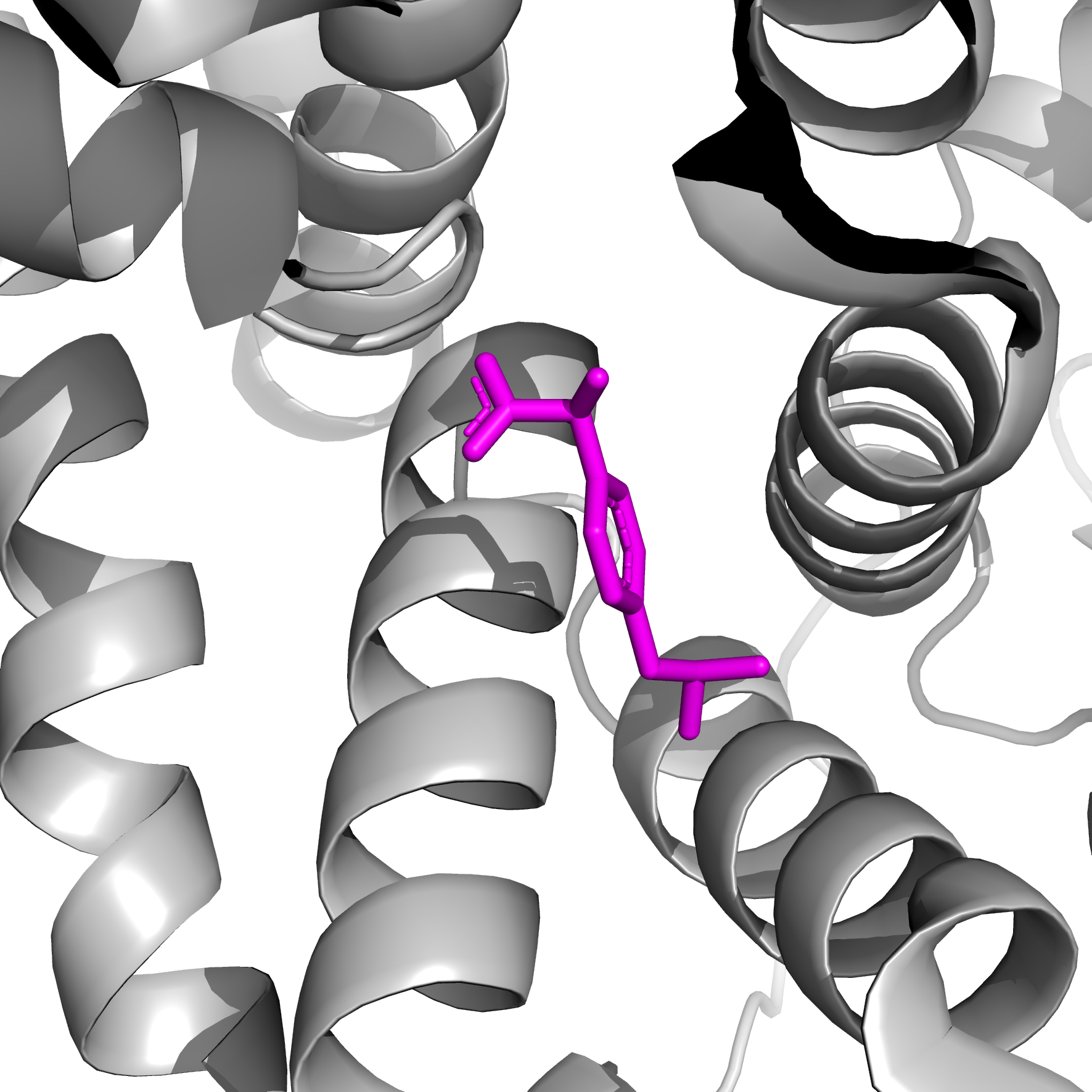

Supplement: Supplementary file 5 — Supplementary Data [file 41467_2023_41427_MOESM5_ESM.zip › Supplementary_Data/Pymol/Ibuprofen_2_zoom.png]
